# Characterization and evolutionary diversification of the phospholipase D gene family in mosses

**DOI:** 10.3389/fgene.2022.1015393

**Published:** 2022-10-13

**Authors:** Jinjie Zhao, Xinyuan Pu, Wenfei Li, Meng Li

**Affiliations:** ^1^ State Key Laboratory for Conservation and Utilization of Bio-Resources in Yunnan, Research Center for Perennial Rice Engineering and Technology of Yunnan, School of Agriculture, Yunnan University, Kunming, Yunnan, China; ^2^ Yunnan Academy of Tobacco Science, Kunming, Yunnan, China

**Keywords:** bryophytes, gene structure, lineage-specific gene duplication, molecular evolution, three-dimensional structure

## Abstract

Plant phospholipase D (PLD) exerts important roles in various biological processes, such as intracellular signaling and morphological development. Our knowledge about early land plant *PLDs* is still underdeveloped. In this study, we identified 84 *PLD* genes in six mosses, i.e., *Physcomitrella patens*, *Ceratodon purpureus*, *Fontinalis antipyretica*, *Pleurozium schreberi*, *Sphagnum magellanicum*, and *Sphagnum fallax*. These *PLDs* were classified into four clades (I–IV). We showed that *PLD* underwent rapid expansion in mosses*.* A total of six conserved domains and two core HKD motifs were detected. Structure analysis uncovered that the moss *PLDs* from within a clade generally exhibited similar exon-intron organization. *Cis*-elements prediction and expression analyses indicated that *P. patens PLDs* had key roles in stress responsiveness and plant development. Particularly, about half of the *P. patens PLDs* (e.g., *PpPLD1*, *PpPLD2*, and *PpPLD5*) were differentially expressed under biotic and abiotic stresses. We also determined the expression pattern of *P. patens PLD* genes in various tissues and at different stages of development. Although the moss, clubmoss, liverwort, and fern *PLDs* evolved largely under functional constraints, we found episodic positive selection in the moss *PLDs*, e.g., *C. purpureus PLD2* and *P. patens PLD11*. We infer that the evolutionary force acting on the *PLDs* may have facilitated moss colonization of land. Our work provides valuable insights into the diversification of moss *PLD* genes, and can be used for future studies of their functions.

## Introduction

Phospholipase D (PLD) is a class of enzymes belonging to the phospholipase superfamily (Wang, 2000; Jang et al., 2012). The hydrolysis of phospholipids into phosphatidic acid has a wide impact on biological processes, such as, intracellular signaling, lipid remodeling, cytoskeletal reorganization, and vesicular trafficking (Zhang et al., 2010). In plants, PLDs are implicated in resistance to abiotic and biotic stresses (Pinosa et al., 2013; Ufer et al., 2017), growth (Hong et al., 2009), and seed development (Ryu et al., 1996). Based on the presence of either the calcium/lipid-binding (C2) or the phox/pleckstrin homology (PX/PH) domains, PLDs can be divided into C2 and PX/PH PLDs. The C2 domain regulates Ca^2+^-dependent activity (Kopka et al., 1998), while the PX/PH domains target phosphoinositide-rich membrane compartments (Hong et al., 2017). In addition to C2 and PX/PH PLDs, there is another type of phospholipase referred to as signal peptide (SP) PLD, which lacks the C2 and PX/PH domains but carrying an N-terminal signal peptide (Selvy et al., 2011). With the help of a signal peptide, SP *PLD* is secreted into the extracellular spaces to hydrolyze its substrates (Qu et al., 2021). In rice, SP *PLD* expression was downregulated during the entire reproductive stage (Singh et al., 2012).

The *Arabidopsis thaliana* PLDs include six subfamilies, i.e., α, β, γ, δ, ε, and ζ (Wang, 2005). The calcium/lipid-binding C2 domain is common in PLDα, β, γ, δ, and ε, and the PX/PH domains are prevalent in PLDζ. The α subfamily has the most redundant PLDs in both *A. thaliana* (three PLDαs) and *Oryza sativa* (eight PLDαs), and this may also be true in other seed plants (Bourtsala et al., 2017). Previous studies indicate that PLDα is involved in response to diverse stresses, including drought, freezing, physical injury, and high salinity (Mane et al., 2007; Hong et al., 2008; Kargiotidou et al., 2010; Bourtsala et al., 2017; Ufer et al., 2017). It is also suggested that *PLDs* from distinct subfamilies are active at different steps of a single biological process. For instance, PLDα and PLDδ are activated at different time points in cotton (*Gossypium hirsutum*) wound signaling (Bourtsala et al., 2017). In addition to those of *A. thaliana* and *O. sativa*, *PLDs* in various angiosperms have been investigated*.* For example, 10 and 16 *PLDs* were found in the genome of pineapple (*Ananas comosus*) and potato (*Solanum tuberosum*), respectively (Hong et al., 2017; Li et al., 2021). When treated with hexaldehyde, the expression of *PLD2* in pineapple fruit was upregulated (Hong et al., 2017). A total of 17 and 11 *PLDs* were identified in poplar (*Populus trichocarpa*), and grape (*Vitis vinifera*), respectively. In poplar, a fast expansion constituted by five species-specific *PLD* gene duplications was reported (Liu et al., 2010).

Mosses are different from angiosperms in many aspects, such as morphology (Beerling et al., 2001), secondary metabolism (Pichersky and Gang, 2000), and life cycle (Boyce, 2008). Knowledge about PLDs in bryophytes is still limited, although several moss genomes have been sequenced (https://www.plabipd.de/plant_genomes_pn.ep). As such, in this study we performed a genome-wide identification of *PLDs* in six moss genomes, i.e., *Physcomitrella patens*, *Ceratodon purpureus*, *Fontinalis antipyretica*, *Pleurozium schreberi*, *Sphagnum magellanicum*, and *Sphagnum fallax*. The phylogenetic and molecular evolution of *PLDs* in mosses were thoroughly explored to elucidate the evolutionary divergence of the moss *PLD* gene family. We also considered conserved sequence characteristics and expression patterns. This provides foundational knowledge for understanding the diversification of moss PLD genes.

## Materials and methods

### Data sources and identification of *PLD* homologs

The proteome and genome files of *P. patens* (v3.3), *C. purpureus* (GG1, v1.1), *Ceratopteris richardii* (v2.1), *Marchantia polymorpha* (v3.1), and *Selaginella moellendorffii* (v1.0) were downloaded from the Joint Genome Institute (DOE-JGI, https://phytozome-next.jgi.doe.gov/). The sequence data of *Azolla filiculoides* and *Salvinia cucullate* were obtained from Fernbase (https://www.fernbase.org/) (Li et al., 2018). The genome data of *F. antipyretica* was acquired from GigaDB (http://gigadb.org/dataset/100748) (Yu et al., 2020). The proteome of *P. schreberi* was retrieved from GitHub project webpage (https://github.com/PycnopodiaD/Pleurozium_schreberi_annotated_genome_files) (Pederson et al., 2019). Using *A. thaliana* and *O. sativa* PLD sequences as queries, BLASTP (Camacho et al., 2009) and hmmsearch (http://hmmer.org/) were employed to search against the collected protein sequence datasets. For *S. magellanicum* and *S. fallax*, online BLASTP searches were performed at the phytozome website (DOE-JGI, https://phytozome-next.jgi.doe.gov/blast-search). Additional BLASTP searches were performed on the NCBI nonredundant (nr) database to obtain as many PLD candidates as possible. A candidate was considered a PLD when either the phospholipase D domain or the phospholipase D C terminal domain was detected.

### Sequence alignment and phylogenetic tree reconstruction

The PLD protein sequences were aligned in MAFFT (v7.450), using the strategy determined by '--auto’ option (Katoh and Standley, 2013). Poorly aligned positions were eliminated using trimAl (v1.4), by allowing a maximum of 30% gaps per sequence (Capella-Gutierrez et al., 2009). IQ-TREE (v1.5.4) with options “-nt AUTO -m TEST -bb 1000 -alrt 1000” was used to identify the best-fit amino acid substitution model for the PLD sequence alignment (LG + I + G was selected according to the BIC score), and then to reconstruct the maximum likelihood phylogenetic tree (Nguyen et al., 2015). Bootstrap values were estimated by 1000 ultrafast bootstrap and SH-like approximate likelihood ratio tests. Bayesian analysis was performed using MrBayes (v3.2.7) (Ronquist et al., 2012). Two independent runs with eight chains each were calculated simultaneously and iteratively until the average standard deviation of the split frequencies was below 0.05 (The Markov chain Monte Carlo chain was run for more than 400 million generations). Trees were sampled every 100 generations. After discarding the first 25% of sampled trees, the posterior probability values were produced. The final tree was visualized in Figtree (v1.4.4, http://tree.bio.ed.ac.uk/software/figtree/).

### Detection of domains, motifs, and signal peptide sequences

Identification of conserved domains was performed by searching the Pfam (Finn et al., 2013) and SMART (Letunic et al., 2020) databases. Conserved motifs were determined using the MEME/MAST software (Bailey and Gribskov, 1998). SignalP (http://www.cbs.dtu.dk/services/SignalP-4.1/) (Nielsen, 2017) was used to predict the signal peptide sequences.

### Gene structure analyses

Gene structure information was retrieved from genome annotation files or GenBank. For genes with two or more transcripts, the exon number referred to the average number of exons. Group differences of exon number were tested by ANOVA followed by a pairwise *t* test with Bonferroni correction (Fisher, 1992). All statistical analyses were performed using R (v3.6.2, https://www.r-project.org/) software.

### Analysis of *cis*-regulatory elements in the promoter region of *PpPLDs*

The 2-kb promoter sequences upstream of the *PpPLDs* extracted from the *P. patens* genome were submitted to the PlantCARE database (http://bioinformatics.psb.ugent.be/webtools/plantcare/html/) to perform the *cis*-acting element analysis. The identified *cis*-elements were displayed using a custom script written in the R programming language (v3.6.2, https://www.r-project.org/).

### Expression analyses

RNA-seq data used for expression analyses were retrieved from the NCBI Sequence Read Archive (SRA) database: *P. patens*: PRJDB6633; *C. purpureus*: PRJNA622159—PRJNA622174, and PRJNA622207; *F. antipyretica*: PRJEB21674; *C. richardii*: PRJNA681601; *A. filiculoide*: PRJNA264391 and PRJEB25913; *S. cucullate*: PRJNA430459; *M. polymorpha*: PRJNA554398, PRJDB6783, and PRJNA350270. Reads were mapped to the reference coding sequences by using kallisto (v0.46.1) (Bray et al., 2016). Expression levels were evaluated by transcripts per million (TPM). If more than one transcript were present, the average TPM would be calculated for following analyses. Expression data for *S. moellendorffii* were extracted from the eFP Browser (http://bar.utoronto.ca/) of Selaginella. No RNA-seq data were publicly available for *S. fallax*, *S. magellanicum*, and *P. schreberi*. For *P. patens PLDs*, expression profiles of different tissues, including spores, caulonema, chloronema, protoplast, rhizoids, gametophore, archegonia, and sporophytes from the S1, S2, S3, and M stages, were collected from the Physcomitrella eFP Browser (Ortiz-Ramirez et al., 2016). The *P. patens* RNA-seq data (PRJNA611083) generated after inoculation of fungal pathogen (*Botrytis cinerea*) were downloaded to analyze biotic stress-induced expression of *PpPLDs*. The RNA-seq data (PRJNA596891) of *P. patens* plants grown under a salinity stress condition (200 mM NaCl) were used to examine abiotic stress-induced expression pattern.

### Molecular evolutionary analyses

The *PLD* coding sequences were aligned using the program “reportGapsAA2NT” implemented in MACSE (v2.05) (Ranwez et al., 2018). To avoid bias introduced by short sequences, 11 out of the identified 132 *PLDs* whose coding sequence length <900 bp (The average coding sequence length of the identified moss, clubmoss, fern, and liverwort *PLDs* is 2400 bp), e.g., *M. polymorpha PLDs* 8–10, were excluded from evolutionary pressure analyses. Molecular evolutionary analyses were applied to the SP *PLDs* separately, because they shared low sequence similarities to C2 and PX/PH *PLDs*. Positive and purifying selection were determined by the ratio of non-synonymous to synonymous nucleotide substitutions (*ω*, also known as *d*_*N*_ */d*_*S*_). The Fast, Unconstrained Bayesian AppRoximation (FUBAR) (Murrell et al., 2013), Mixed Effects Model of Evolution (MEME) (Murrell et al., 2012), Branch-Site Unrestricted Statistical Test for Episodic Diversification (BUSTED) (Murrell et al., 2015), and adaptive Branch-Site Random Effects Likelihood (aBSREL) (Smith et al., 2015) methods were run using the HyPhy software package (Kosakovsky Pond et al., 2020). Changes in selection intensity were estimated using the RELAX method (Wertheim et al., 2015). The branch-site model analysis was conducted using the CODEML program from the PAML package (v4.9j) (Yang, 2007).

### Three-dimensional structure prediction

AlphaFold v2.0 (Jumper et al., 2021) was used to predict the 3D structures for PpPLD11, with the resources and default parameters provided at the ColabFold website (https://colab.research.google.com/github/sokrypton/ColabFold/blob/main/AlphaFold2.ipynb). To perform homology modeling, the protein sequence of PpPLD11 was analyzed by searching the SWISS-MODEL database (http://swissmodel.expasy.org/) (Waterhouse et al., 2018). The quality of the predicted 3D structure was assessed using SAVES (https://saves.mbi.ucla.edu/). Pymol software (https://pymol.org/) was applied to visualize the 3D structure.

## Results

### Identification and annotation of *PLDs* in available moss genomes

**FIGURE 1:**
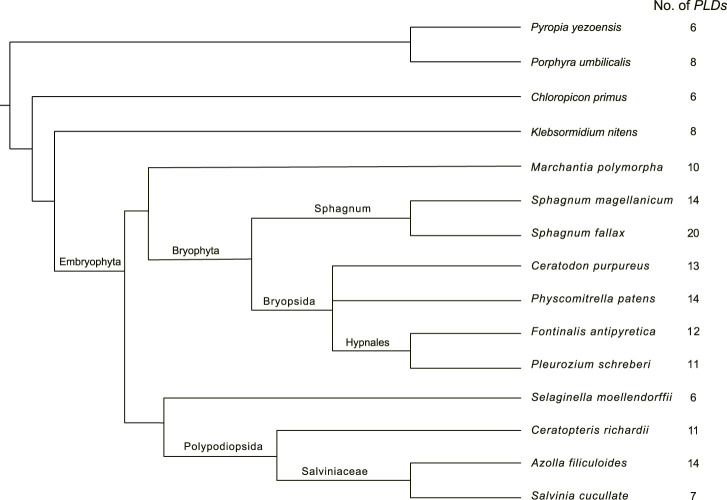
Species tree of the studied mosses, clubmosses, ferns, liverworts, and algae. This tree was drawn according to Harris et al. (2020). The column on the right indicates the number of *PLD* genes.

To gain insights into the distribution of *PLD* in mosses, we mined homologous *PLD* genes from the genomes and proteomes of six bryophytes, *P. patens*, *C. purpureus*, *F. antipyretica*. *P. schreberi*, *S. magellanicum*, and *S. fallax*. A total of 14, 13, 12, 11, 14, and 20 *PLDs* were identified in these species, respectively (Supplementary Table S1). Further, we investigated the presence of *PLDs* in another five early land plant species, categorized as clubmosses (*S. moellendorffii*), ferns (*C. richardii*, *A. filiculoides*, and *S. cucullate*), and liverworts (*M. polymorpha*). In total, 132 *PLD* homologous sequences were identified (Supplementary Table S1). As shown in Figure 1, the number of *PLD* genes varies widely between species, ranging from six (*S. moellendorffii*) to 20 (*S. fallax*). These *PLD* homologs were named according to a common nomenclature consisting of the first letters of both the genus (upper case) and the species (lower case), followed by the *PLD* identifier and a number arranged by its order of domain conservation.

**FIGURE 2:**
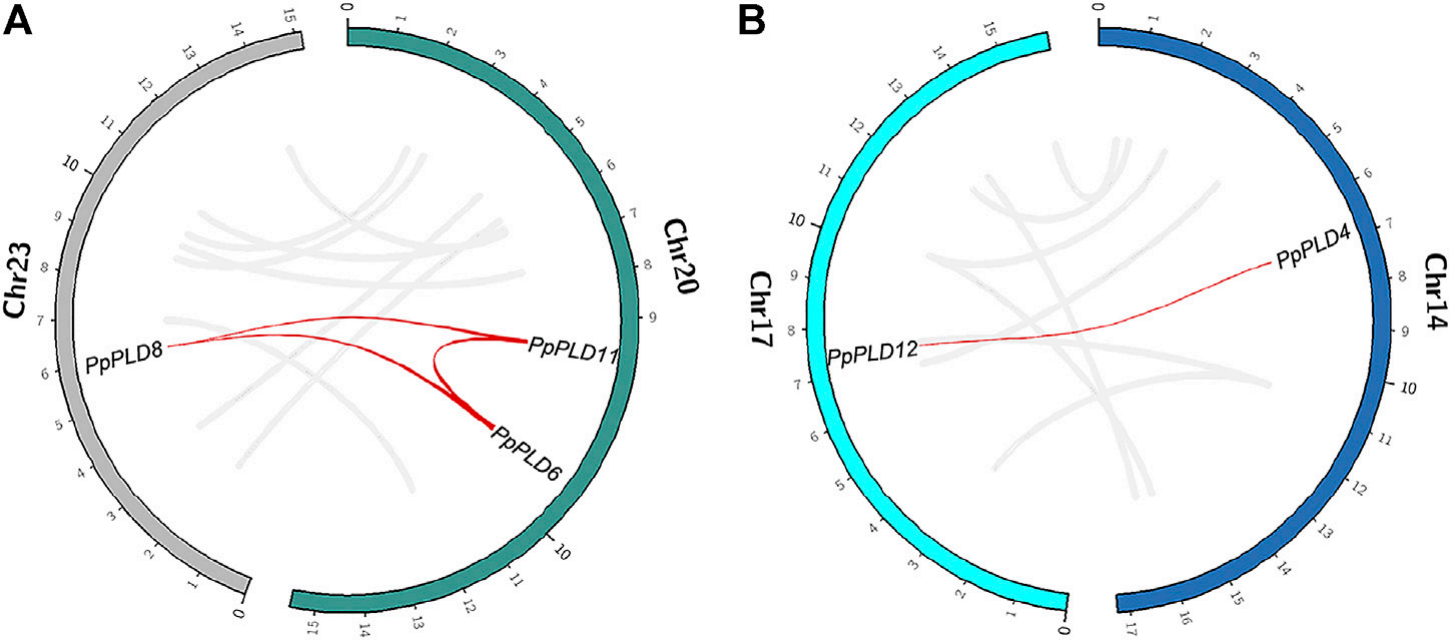
Genomic location of species-specific duplicated *PLDs* in *P. patens*. **(A)**. *PLDs 6*, *8*, and *11*. **(B)**. *PLDs 4* and *12*. Gray lines indicate synteny blocks, and red lines indicate species-specific *PLD* duplications.

We further analyzed the genomic location of the identified *PLDs*. In the *P. patens* genome, 12 of 14 *PLDs* were mapped separately onto 12 chromosomes (chromosomes 2, 3, 7, 8, 10, 12–14, 17, 18, 22, and 23. Figures 2A,B, and Supplementary Figure S1). On chromosome 20, *PpPLD6* was located nearly adjacent to *PpPLD11*. Similar patterns were also observed in *S. magellanicum*, *S. fallax*, *C. richardii*, and *C. purpureus* (Supplementary Figure S1). Such co-locations were not found for *PLDs* in *F. antipyretica*, *P. schreberi*, *S. moellendorffii*, *A. filiculoides*, *S. cucullate*, and *M. polymorpha*, partly because genomes of these species were not fully assembled.

### Origin and diversification of the moss *PLDs*

**FIGURE 3:**
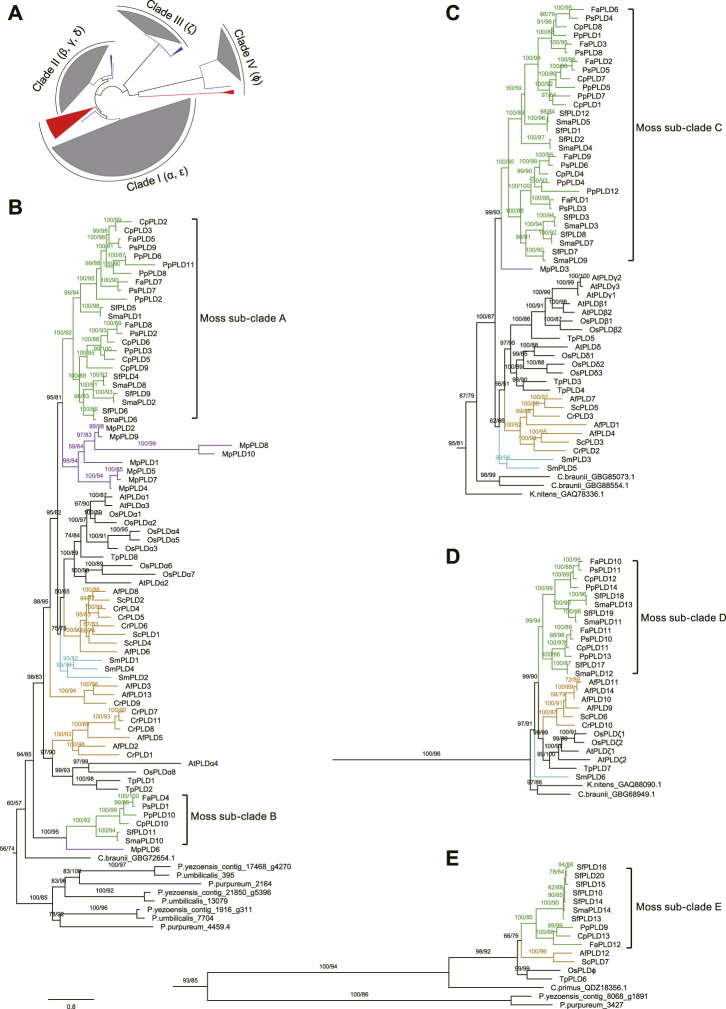
Phylogenetic unrooted tree of PLDs from mosses, clubmosses, liverworts, ferns, angiosperms, and algae. Because it was not clear when the C2, PX/PH, and SP PLD subfamilies diverged (perhaps long before the emergence of red algae), no outgroup was used for this tree. **(A)**: A simplified overview of the entire phylogenetic tree with clades I, II, III, and IV collapsed. Blocks colored in grey, red, and blue represent PLDs of land plants (mosses, clubmosses, liverworts, ferns, and angiosperms) and red and green algae, respectively. **(B–E)**: Phylogenetic topologies within clades I, II, III, and IV, respectively. The original phylogenetic tree is displayed in Supplementary Figure S2. The sequence alignment used was provided in supplementary data sheet 1. Above branches are bootstrap supports from maximum likelihood and Bayesian analyses, respectively. The bootstrap value below 50% is not shown. The green, purple, orange, and cyan color on the branches refer to mosses, liverworts, ferns, and clubmosses, respectively. Scale bar represents substitution numbers per amino-acid site for **(B–E)**. The abbreviations used are as follows: Pp, *P. patens*; Cp, *C. purpureus*; Fa, *F. antipyretica*; Ps, *P. schreberi*; Sma, *S. magellanicum*; Sf, *S. fallax*; Sm, *S. moellendorffii*; Cr, *C. richardii*; Af, *A. filiculoides*; Sc, *S. cucullate*; Mp, *M. polymorpha*; At, *A. thaliana*; Os, *O. sativa*; Tp, *T. plicata*.

Phylogenetic analyses were carried out on the above-mentioned moss, clubmoss, fern, and liverwort *PLDs*, together with another 55 from *A. thaliana*, *O. sativa*, *Thuja plicata*, and red and green algae to explore the origin of the moss *PLDs*. The phylogenetic trees reconstructed using the maximum likelihood and Bayesian methods shared a similar topology. As displayed in Figure 3; Supplementary Figure S2, the green plant *PLDs* are classified into four clades (I–IV). The C2 *PLDs* clustered within clades I and II, and PX/PH *PLDs* clustered within clade III. The SP *PLDs* were located in clade IV. For *A. thaliana* and *O. sativa*, the α and ε *PLDs* were grouped into clade I. The β, γ, and δ *PLDs* were grouped into clade II. The ζ and ϕ *PLDs* were clustered in clades III and IV, respectively.

According to the phylogeny, the most ancient *PLDs* in clade I (*PLDαs* and *PLDεs*) could be found in three red algae species *Pyropia yezoensis*, *Porphyridium purpureum*, and *Porphyra umbilicalis*. For clade II (β, γ, and δ) *PLDs*, orthologs from *Klebsormidium nitens* and *Chara braunii*, two charophytic algae very closely related to land plants, were placed on the root. Similarly, the most ancient orthologs of clade III *PLDs* (*PLDζs*) came from *K. nitens* and *C. braunii*. For clade IV *PLDs* (*PLDϕs*), we identified one homolog from *Chloropicon primus*, a tiny marine green alga, and two homologs from *P. yezoensis* and *P. purpureum*.

Within each *PLD* clade, the *PLDs* of peatmosses and true mosses (Bryopsida) were clustered tightly together, forming five separate sub-clades (A–E) (Figure 3), consistent with their taxonomic classification. It was noteworthy that three of these five moss sub-clades (i.e., sub-clades A, C, and D) could be further divided into two groups, each comprising *PLDs* from *S. magellanicum*, *S. fallax*, *C. purpureus*, *P. patens*, *F. antipyretica*, and *P. schreberi*. Furthermore, phylogenetic analysis suggested that the *PLD* family underwent four species-specific gene duplications in mosses, that is, *CpPLDs 2* and *3*; *PpPLDs 6*, *8* and *11*; *PpPLDs 4* and *12*; *SfPLDs 10*, *13*–*16*, and *20* (Figure 3).

### Conserved domains and motifs in the moss PLDs

**FIGURE 4:**
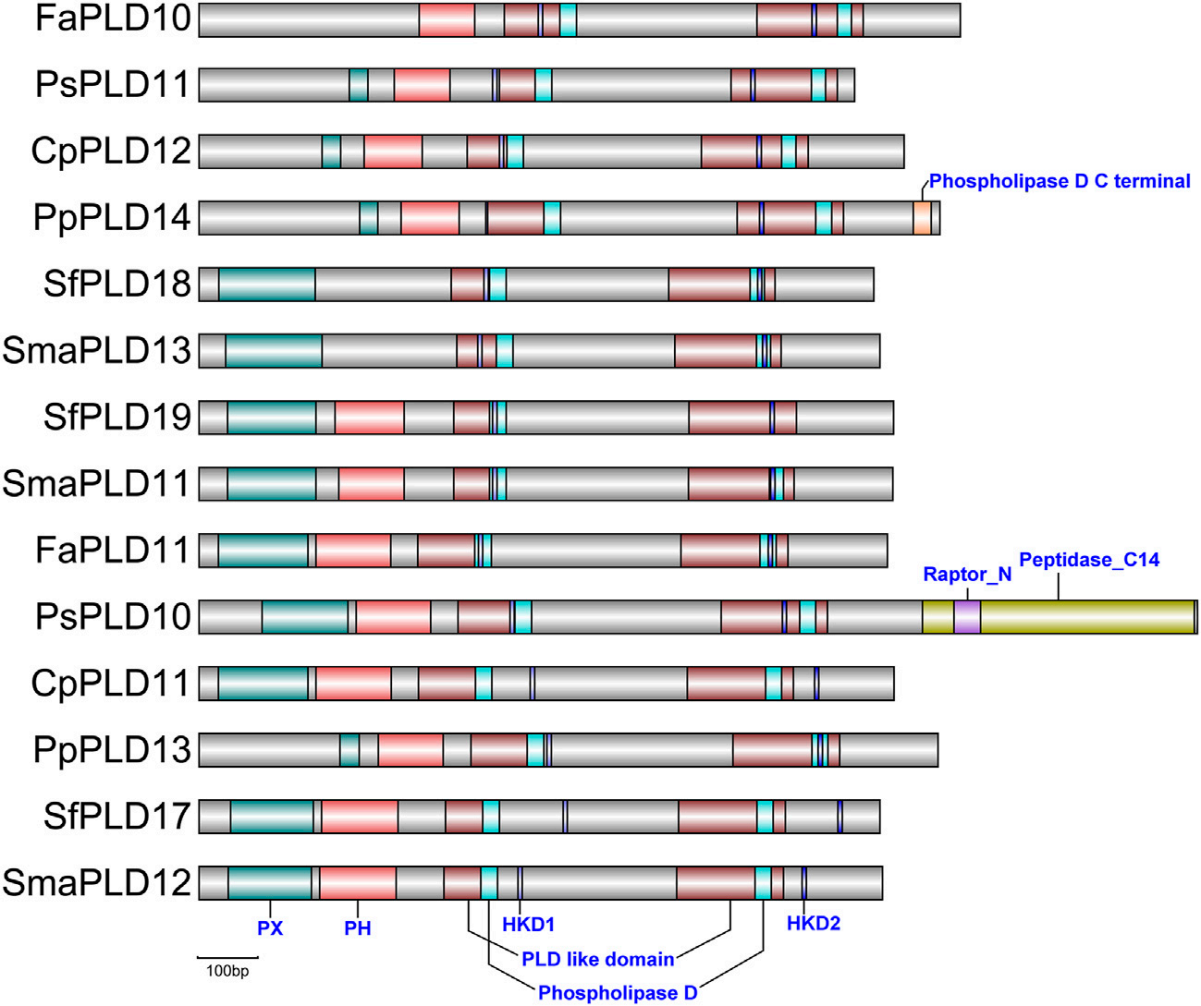
Conserved domains and motifs observed in moss clade III (PX/PH) *PLDs.* The full conserved domains and motifs detected in all PLDs were displayed in Supplementary Table S2 due to page limitation. The PLDs are ordered according to the phylogenetic tree displayed in Figure 3. The grey bars indicate the PLD protein sequences. The phospholipase D (Pfam accession number: PF00614) and PLD like domains (Pfam accession number: PF13091/PF13918) are indispensable for hydrolyzing phosphatidylcholine into phosphatidic acid. The phospholipase D C terminal (Pfam accession number: PF12357) can bind to calcium. The PX domain (Pfam accession number: PF00787) contains a pbox consensus sequence. The PH (Pfam accession number: PF00169) is a pleckstrin homology domain approximately 120 amino acids in length. The HKD1 and HKD2 motifs contain the core sequence “H×K××××D”.

The phospholipase D, phospholipase D C terminal, C2, and PX/PH domains commonly reported in angiosperm PLDs were identified in moss PLDs (Supplementary Table S2; Supplementary Figure S3). In mosses, clubmosses, ferns, and liverworts, most of the obtained PLDs contained the phospholipase D domain, except for MpPLDs 8–10 and AfPLD14 (Supplementary Table S2). The phospholipase D C terminal and C2 domain were found in moss PLDs within clades I and II (α, β, γ, ε, and δ PLDs), with a few exceptions. For example, the C2 domain was missing in PpPLD11, PpPLD12, PsPLDs 1–4 and 8, FaPLDs 4 and 6, and CpPLD10. The PX/PH domains were specific to clade III PLDs (PLDζs) (Figure 4). However, in moss clade III PLDs (PLDζs), FaPLD10 did not contain the PX domain, and SfPLD18 and SmaPLD13 did not have the PH domain. In addition, signal peptide sequences were observed in the moss clade IV (ϕ) PLDs (SfPLDs 10, 14, and 20, SmaPLD14, FaPLD12, and PpPLD9).

The HKD (H×K××××D) motifs are also frequently noted characteristics in angiosperm PLDs (Elias et al., 2002). Using the MEME/MAST software (Bailey and Gribskov, 1998), we found that HKD1 and HKD2 motifs were present in most of the moss PLDs, with a few exceptions (Figure 4; Supplementary Figure S3; Supplementary Table S2)*.* For instance, the HKD1 motif was absent in PpPLD12, PsPLDs 1 and 2, FaPLD12, and SfPLDs 14, 15, and 20. A total of 18 moss PLDs lacked the HKD2 motif, e.g., CpPLD13, PpPLD9, and SmaPLD14.

### Gene structure analyses in the moss *PLDs*

Given that the diversity of gene structure is important for the evolution of a gene family (Liu et al., 2009), the exon-intron structures of moss *PLDs* were analyzed. As shown in Supplementary Figure S4, *PLDs* within a clade generally share similar exon-intron structures, in a manner roughly consistent with their phylogenetic relationships. For instance, both *SfPLD4* and *SmaPLD8* contained five exons, with almost identical exon-intron structures. In these two genes, the first and second exons were about 600 and 150 bp, respectively, with the first intron measuring about 170 bp. Another representative case was the pair of *SfPLD11* and *SmaPLD10*, both of which possessed three exons, and shared highly similar gene structures. When the *PLDs* of clubmosses, ferns, and liverworts were taken into consideration, the clade II (β, γ, and δ) *PLDs* had the largest average number of exons, approximately 9.6 per gene (Supplementary Figure S5), significantly more than those of clades I and IV (*α*, *ε*, and *ϕ PLDs*, 3.8 and 6 average exons per gene, respectively). Interestingly, PX/PH *PLDs* (ζ, clade III) of peatmosses and true mosses had an average of 2.6 exons per gene, significantly fewer than those of ferns, clubmosses and *K. nitens* (on average 16.9, 20, and 16 exons per gene, respectively), suggesting intron loss in the moss PX/PH (ζ) *PLDs*.

### Examination of *cis*-elements in promoters of *P. patens PLD* genes

**FIGURE 5:**
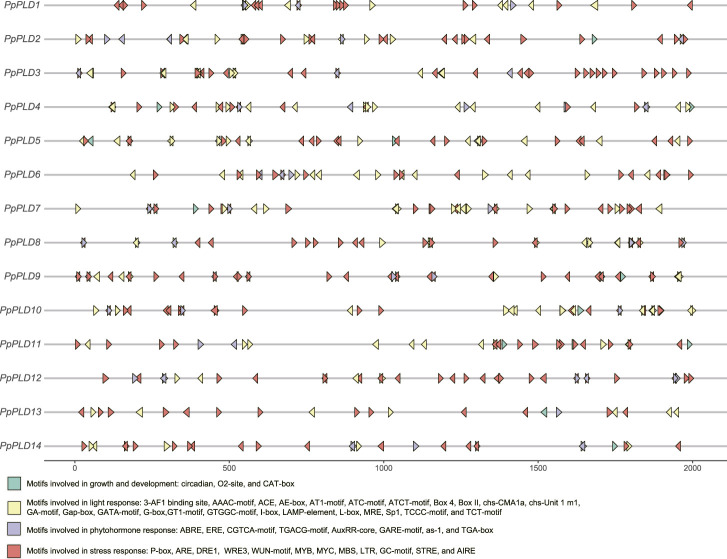
Predicted *cis*-elements in the promoter regions of the *PpPLD* genes.

*Cis*-elements of the promoter region can regulate gene transcription and function. As shown in Figure 5, the *cis*-acting elements observed in *PpPLD* gene promoters can be divided into four general groups, i.e., stress-, light-, phytohormone-, and growth and development-correlated motifs. Five stress associated (drought, salt, low-temperature, wound, and anaerobic) responsive elements consisting of P-box, ARE, DRE1, WRE3, WUN-motif, MYB, MYC, MBS, LTR, GC-motif, STRE, and AIRE, constituted the most redundant *cis*-elements in *PpPLD* genes. Specifically, several stress related elements were extensively dispersed in *PpPLDs 3* and *9*. The light response involved motifs were the second-most enriched *cis*-elements in the *PpPLD* gene promoters. The identified light responsive elements included 3-AF1 binding site, AAAC-motif, ACE, AE-box, AT1-motif, ATC-motif, ATCT-motif, Box 4, Box II, chs-CMA1a, chs-Unit 1 m1, GA-motif, Gap-box, GATA-motif, G-box, GT1-motif, GTGGC-motif, I-box, LAMP-element, L-box, MRE, Sp1, TCCC-motif, and TCT-motif. Additionally, we found phytohormone-correlated motifs, including ABRE, ERE, CGTCA-motif, TGACG-motif, AuxRR-core, GARE-motif, as-1, and TGA-box. The plant growth and development related motifs were comprised of circadian (involved in circadian regulatory), O2-site (associated with zein metabolism regulation), and CAT-box (related to meristem expression).

### Expression profiles of the moss *PLD* genes

**FIGURE 6:**
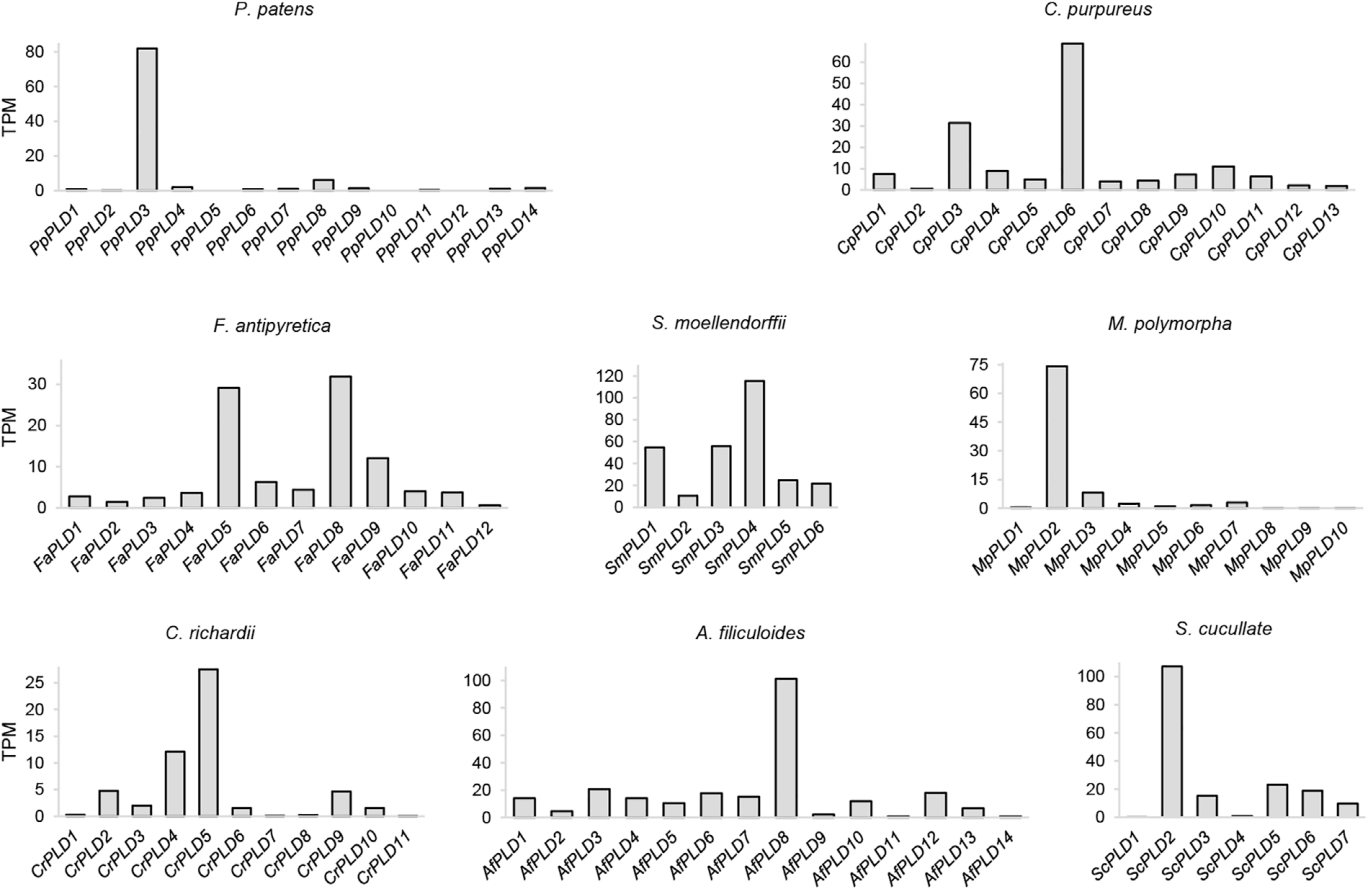
Expression profiles of the moss, clubmoss, liverwort, and fern *PLDs*. This figure is based on data collected from different RNA-seq experiments and demonstrates the expression of *PLDs* for each species regardless of the tested conditions. The expression value is not intended for comparison among species. TPM: transcripts per million. The BioProject IDs of the transcriptome experiments are listed in Supplementary Table S3.

The expression profile of a gene can provide useful information about its molecular function (Brown et al., 2005; Hansen et al., 2014). To analyze the transcriptional activity of the *PLDs*, we performed expression analyses. Publicly available RNA-seq data were found for *P. patens*, *C. purpureus*, *F. antipyretica*, *C. richardii*, *A. filiculoide*, *S. cucullate*, and *M. polymorpha* (Supplementary Table S3). For each of these species, an average of 165.25 GB of transcriptome data were collected. The expression level was estimated by TPM. As a result, most of the investigated moss, clubmoss, fern, and liverwort *PLDs* were expressed under certain conditions (Figure 6 and Supplementary Table S4). For instance, 11 of 14 *P. patens PLDs* were expressed; among these, *PpPLD3* was the most highly expressed (TPM = 81.88), suggesting it had a critical function in these experimental studies. Additionally, of the tested plants, in species other than *F. antipyretica* and *A. filiculoides*, both the highest and lowest expressed *PLDs* were found in clade I (*PLDαs* and *PLDεs*). These results suggested that clade I *PLDs* maintained differentiated functions in the tested conditions.

### Analysis of *PLD* expression profiles in different tissues of *P. patens*

**FIGURE 7:**
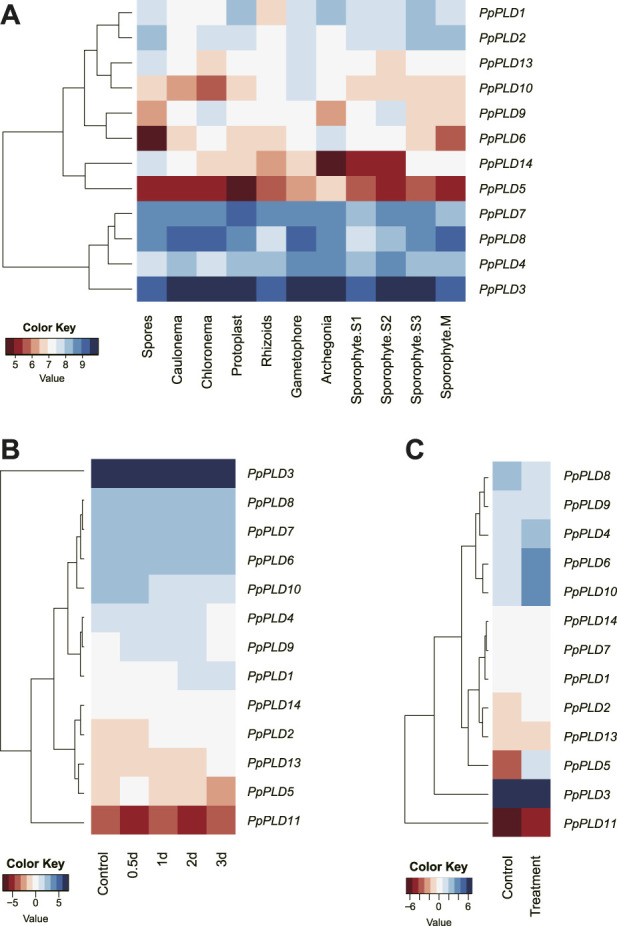
Hierarchical clustering of the *P. patens PLD* expression profiles. **(A)**. Expression data in different tissues including caulonema, chloronema, protoplast, rhizoids, gametophore, archegonia, spores, and four sporophytic stages. The sporophyte S1, S2, S3, and M stages referred to the development stages 7, 15, 20, and 33 days after fertilization (Ortiz-Ramirez et al., 2016). **(B)** Expression data obtained from infected and uninfected *P. patens* plants. Total RNA from infected plants were extracted 0.5, 1, 2, and 3 days after inoculation with *Botrytis cinerea*. **(C)** Transcriptional expression of *PpPLDs* under salt stress and control conditions. *P. patens* plants were grown under salinity (200 mM NaCl) and control (0 mM NaCl) conditions for 7 days. Genomic RNA was extracted from protonema tissue. The log-transformed values of the expression levels of *PLD* genes were used (original data are shown in Supplementary Table S4).

Tissue-specific expression profiles of *PpPLD* genes were examined in 11 various tissues, including caulonema, chloronema, protoplast, rhizoids, gametophore, archegonia, spores, and four developmental sporophytic stages, i.e., sporophyte S1, S2, S3, and M, using microarray expression data obtained from the Physcomitrella eFP Browser. In line with the RNA-seq results, *PpPLD3* was the top expressed in all tested tissues. For most *P. patens* tissues, the second highest expression levels were detected in *PpPLDs 4*, *7*, and *8*, and the lowest detectable expression level was found in *PpPLD5* (Figure 7A). The results also showed that *PpPLD* expression varied among tissues. For instance, *PpPLDs 1* and *7* were preferentially expressed in protoplast; *PpPLDs 2*, *13*, and *14* were most highly expressed in spores; Expression of *PpPLDs 3*, *4*, and *10* were strongest in gametophore; expression of *PpPLDs 5* and *6* were highest in archegonia; and *PpPLDs 8* and *9* were most abundantly expressed in sporophyte M and chloronema, respectively. Additionally, in chloronema, expression of *PpPLDs 2*, *4*, and *9* were moderate (the expression values ranged from 2000 to 3000). While in gametophore, moderate expression was observed in *PpPLDs 1*, *2*, *10*, and *13*. In closely related tissues, such as caulonema and chloronema, several *PLD* genes showed similar expression levels, e.g., *PpPLDs 1*, *3*, *5*, *7*, and *8*. During the sporophyte development, *PpPLD6* was highly expressed at the S1 stage and decreased after that, whereas expression of *PpPLD8* continued to increase after the S1 stage.

### Expression analyses of *PpPLDs* under biotic and abiotic stress conditions

The *cis*-element analysis suggested that *PpPLDs* played an important role in stress-responsive behavior. Therefore, we analyzed expression patterns of *PpPLD* genes under different biotic and abiotic stress conditions, including fungal pathogens and salt. For biotic stress, the RNA-seq data were generated at 0.5, 1, 2, and 3 days after infection of *Botrytis cinerea*. Expression of seven *PpPLDs* (i.e., *PpPLD1*, *PpPLD*2, *PpPLD5*, *PpPLD6*, *PpPLD10*, *PpPLD11*, and *PpPLD13*) were found to be significantly induced at least in one time point (≥1.5-fold change, Figure 7B). For instance, *PpPLD2* showed higher expression at 1 dpi (days after inoculation) (1.52 fold), 2 dpi (1.90 fold), and 3 dpi (1.71 fold). *PpPLD11* was upregulated at 0.5 dpi (2.67 fold), and downregulated at 2 dpi (2.00 fold). Under salinity stress, eight *PpPLDs* showed differential expression (≥1.5-fold change, Figure 7C). Upregulated genes included *PpPLD*2 (1.71 fold), *PpPLD4* (2.38 fold), *PpPLD*5 (27.55 fold), *PpPLD6* (3.47 fold), *PpPLD10* (5.53 fold), and *PpPLD11* (2.29 fold). *PpPLD7* and *PpPLD8* exhibited decreased expression (1.51 and 2.15-fold, respectively) when treated with NaCl.

### Selection pressures on the evolution of *PLD* genes of mosses

To unravel the molecular evolutionary mechanisms underlying the *PLDs* of mosses, clubmosses, ferns, and liverworts, the FUBAR method which can detect pervasive positive and negative selection at specific sites, alignment-wide (Murrell et al., 2013), was applied. We found no sites under positive selection, 193 sites from clades I–III (*α*, *β*, *γ*, *δ*, *ε*, and *ζ PLDs*), and 282 sites from clade IV (*PLDϕs*), with evidence of purifying selection at a posterior probability >0.9 (Supplementary Table S5). Because selection is often transient rather than pervasive, we therefore restricted the analysis to moss *PLDs*, and adopted the MEME method (Murrell et al., 2012), which can identify individual sites that have experienced episodic positive selection under a proportion of branches. We found evidence of positive selection at 11 sites of the moss clades I–III (*α*, *β*, *γ*, *δ*, *ε*, and *ζ*) *PLDs*, and at 16 sites of the moss clade IV *PLDs* (*PLDϕs*) (*p* < 0.05, Supplementary Table S5). The MEME method can detect selection at site-level, but cannot detect gene-wide selection. To detect gene-wide positive selection acting on the moss *PLDs*, we used the BUSTED method which can test for positive selection on a subset of branches and at a proportion of sites (Murrell et al., 2015). As a result, about 0.90% sites of the moss *PLDs* from clades I–III (*α*, *β*, *γ*, *δ*, *ε*, and *ζ PLDs*) were found to be evolving with ω >1 [likelihood ratio test statistic (LRT) = 34.56, *p* = 1.56 × 10^–8^, Table 1]. For the moss *PLDs* from clade IV (*PLDϕs*), 3.74% sites showed evidence of episodic positive selection (*ω* = 8.48, LRT = 19.20, *p* = 3.38 × 10^–5^, Table 1). In addition to site-level and gene-wide selections, we also examined the branch-level episodic diversifying selection acting on the moss *PLDs*, using the aBSREL method (Smith et al., 2015). Episodic positive selections were identified on 3% sites of *CpPLD*2 (ω2 = 455.26, LRT = 21.57, *p* = 8.87 × 10^–4^) and 22% sites of *PpPLD11* (ω2 = 9.62, LRT = 17.07, *p* = 0.01, Supplementary Table S5). Since the aBSREL method does not report exactly which sites are under positive selection, we additionally performed the branch-site model analysis (by setting the *CpPLD2* and *PpPLD11* as foreground branches) using the CODEML program implemented in the PAML package. The likelihood ratio test comparing the modified model A (alternative model, fix_omega = 0) with the corresponding null model (fix_omega = 1) reached a significant level of *p* = 3.46 × 10^–7^. The alternative model reported that 24% sites of *CpPLD2* and *PpPLD11* had an estimate of ω2 = 7.44, among which 12 were statistically significant (*p* > 95%; 188T, 225K, 227P, 234E, 238L, 242C, 262K, 263F, 265Y, 334K, 366R, and 375Q of *PpPLD11*; Table 2).

**TABLE 1:** The branch-site unrestricted statistical test for episodic diversification (BUSTED) results for moss *PLDs*.

	Model	log L	LRT	Branch set	ω1 (proportion)	ω2 (proportion)	ω3 (proportion)
clades I–III (α, β, γ, δ, ε, and ζ)	Constrained	−30023.40	34.56 *p* = 1.56 × 10^–8^	Background	0.01 (54.66%)	0.11 (43.43%)	104.02 (1.92%)
				Test	0.00 (65.04%)	0.08 (29.09%)	1.00 (5.87%)
	Unconstrained	−30006.20		Background	0.01 (56.13%)	0.11 (41.97%)	98.24 (1.90%)
				Test	0.00 (73.12%)	0.20 (25.98%)	11.94 (0.90%)
clade IV (ϕ)	Constrained	−7920.12	19.20 *p* = 3.38 × 10^–5^	Background	0.03 (72.64%)	0.11 (15.36%)	1.00 (12.00%)
				Test	0.00 (32.16%)	0.08 (54.19%)	1.00 (13.65%)
	Unconstrained	−7910.52		Background	0.02 (68.71%)	0.13 (20.72%)	1.00 (10.57%)
				Test	0.00 (20.70%)	0.10 (75.56%)	8.48 (3.74%)

log L, log likelihood; LRT, likelihood ratio test statistic. Test branches: the moss *PLDs*; Background branches: the clubmoss, liverwort, and fern *PLDs*.

**TABLE 2:** The branch-site model test result for *CpPLD2* and *PpPLD11*.

Model	lnL	2ΔlnL	Estimates of parameters	Positively selected sites
Model A	-29282.01	25.97 *p* = 3.46 × 10^–7^	*p0* = 0.75 *p1* = 4.84 × 10^–3^ (*p2*+*p3* = 0.24)	188T (0.98) 225K (1.00)
			ω0 = 0.06 ω1 = 1.00 ω2 = 7.44	227P (1.00) 234E (1.00)
				238L (1.00) 242C (0.99)
Model A Null	-29295.00		*p0* = 0.62 *p1* = 3.77 × 10^–3^ (*p2*+*p3* = 0.38)	262K (1.00) 263F (1.00)
			ω0 = 0.06 ω1 = 1.00 ω2 = 1.00	265Y (0.99) 334K (1.00)
				366R (1.00) 375Q (1.00)

lnL, log likelihood; 2ΔlnL, twice the log-likelihood difference between the two models. Positively selected sites were produced by Bayes Empirical Bayes analysis. Amino acids referred to *PpPLD11*.

To ascertain if the selection pressures (both positive and purifying) observed on the moss *PLDs* were relaxed or intensified, we adopted the RELAX method which introduces an intensity parameter (k). k >1 and k <1 indicate selective relaxation and intensification, respectively (Wertheim et al., 2015). Intensified selection was found in the moss sub-clade B *PLDs* comprising *FaPLD4*, *PsPLD1*, *PpPLD10*, *CpPLD10*, *SfPLD11*, and *SmaPLD10* (k = 4.21, *p* = 1.36 × 10^–6^, LRT = 23.34), and in the moss sub-clade D (*ζ*) *PLDs* (k = 1.23, *p* = 0.01, LRT = 6.62). Additionally, we found evidence of relaxed selection in the moss sub-clade C *PLDs* (*β*, *γ* and *δ*) (k = 0.75, *p* < 0.05, LRT = 3.96).

### Three-dimensional structure prediction of PpPLD11

**FIGURE 8:**
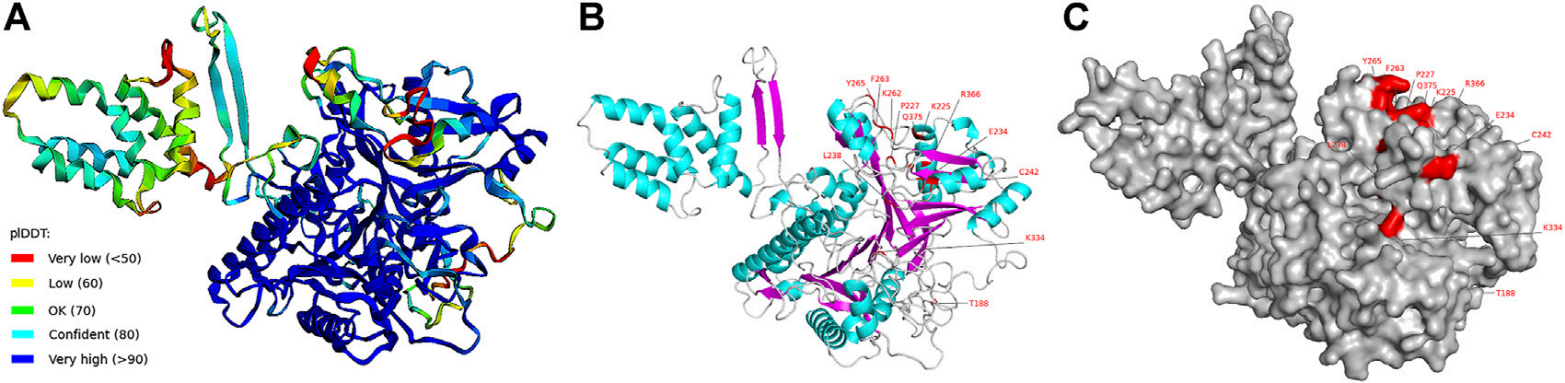
Three-dimensional structure of PLD11 predicted by AlphaFold. **(A)** Schematic diagram of 3D structure of PpPLD11 colored by the pLDDT score. **(B)** View of positively selected amino acid sites on the 3D structure of PpPLD11. Helix, sheet, and loop are represented by cyan, magenta, and light grey, respectively. **(C)** View of positively selected amino acid sites on the surface of PpPLD11. The positively selected sites are colored in red.

To gain insights into the function of the positively selected sites of *PpPLD11*, we predicted its crystal structure using AlphaFold (Figure 8). The per-residue confidence score (pLDDT, a value between 0 and 100) indicated that the PpPLD11 protein tertiary structure model had a high confidence. For homology modeling method, the protein sequence of *Arabidopsis* PLDα1 (6 kz8.1A, which shared an identity of 56.9% with PpPLD11) was used as the template. The 3D structure of the PpPLD11 protein constructed by homology modeling had a global model quality estimate (GMQE, a value between 0 and 1) of 0.77 (Supplementary Figure S6). On these two models, nine positively selected sites (188T, 225K, 227P, 234E, 238L, 262K, 263F, 265Y, and 334K) were present in the loop region, and three positively selected sites (242C, 366R, and 375Q) were distributed in the helix. Interestingly, these 12 positively selected sites were located on the surface of the 3D structures (Figure 8C and Supplementary Figure S6).

## Discussion

Despite that the *PLD* gene members have been widely documented in various angiosperms, such as *A. thaliana*, *Gossypium arboretum*, and *Camellia sinensis*, (Qin and Wang, 2002; Tang et al., 2016; Roshan et al., 2021), little is known about the diversification of moss *PLDs*. To fill this gap in our knowledge, we performed a genome-wide comparative analysis of *PLD* genes among six mosses, one clubmoss, one liverwort, and three ferns. The 132 *PLDs* identified were not considered the result of sequencing contamination, because they were mapped to well-assembled scaffolds or chromosomes, and most of them were found to be expressed. However, it is still possible that several *PLD* genes (e.g., *M. polymorpha PLDs 8–10*) may suffer from genome assembly errors considering that their protein sequences are unusually short.

**FIGURE 9:**
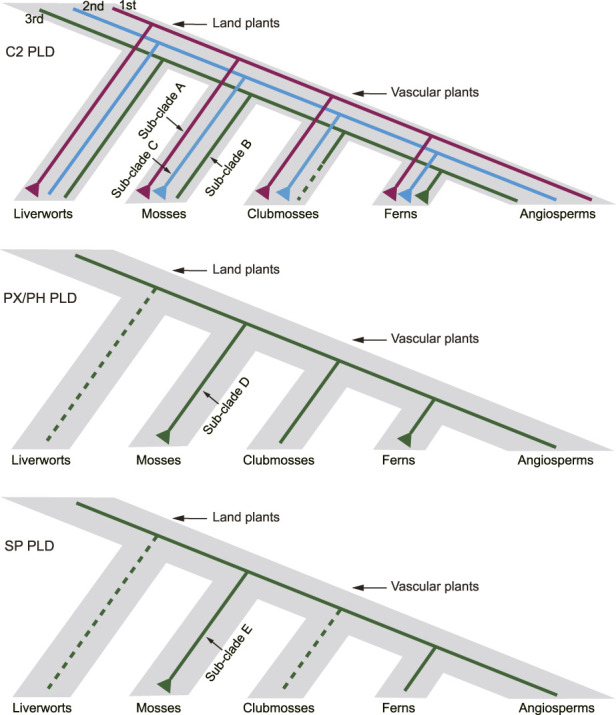
Hypothetical scheme of the expansion history of *PLDs* in mosses, clubmosses, liverworts, and ferns. Dashed lines indicate putative gene loss events.

The phylogenetic analysis divided the plant *PLDs* into four distinct clades (I–IV), mostly consistent with the categorization of C2, PX/PH, and SP *PLDs*. Our results suggested that the origin of land plant C2, PX/PH, and SP *PLDs* could be traced back at least to the emergence of green algae. Furthermore, we reconstructed a hypothetical evolutionary pattern for moss *PLDs* (Figure 9). Three ancestral C2 *PLDs* (clades I and II, i.e., *α*, *β*, *γ*, *ε*, and *δ PLDs*) were predicted to have diverged before the most recent common ancestor (MRCA) of land plants. The first and second ancestral C2 *PLD* copies (e.g., the homologs of the moss sub-clades A and C, respectively) were expanded in mosses. The rapid expansion of the first ancestral copy was also observed in all investigated liverworts, clubmosses, and ferns. The third ancestral C2 *PLD* copy (e.g., the homologs of the moss sub-clade B) was highly conserved in mosses, with exactly one homolog per species. For both PX/PH and SP *PLDs*, only one ancestral *PLD* was present in the MRCA of land plants. The primary expansion of moss PX/PH *PLD* had occurred before the splitting of the bryophyte lineage. Of note, duplication of SP *PLD* was observed in *S. fallax* but not in other investigated mosses. The *S. fallax* SP *PLDs* are believed to emerge from one segmental duplication and four tandem duplications.

In *P. patens*, it was reported that two separate whole genome duplication (WGD) events occurred 27–35 and 40–48 million years ago (Lang et al., 2018). Here, we demonstrated that WGD greatly contributed to the recent *P. patens*-specific *PLD* duplications. This expansion pattern differed from the small-scale gene duplications (e.g., tandem and proximal gene duplications) that mainly occurred to *PLDs* of *S. fallax* and *C. purpureus*. For instance, *PpPLD2* and *PpPLD8* were located on chromosomes 8 and 23, respectively, and both *PpPLD6* and *PpPLD11* were anchored to chromosome 20. Together with the fact that chromosomes 8, 20, and 23 are derived from a common ancestor (Lang et al., 2018), it is likely that the primary WGD generated *PpPLD2* and the ancestor of *PpPLDs 6*, *8*, and *11*, and the second WGD yielded *PpPLD8* and the ancestor of *PpPLD6* and *PpPLD11*, with a subsequent tandem duplication on chromosome 20 producing the current *PpPLD6* and *PpPLD11*.

In the same PLD clade, the domains, motifs, and exons had similar arrangements, lending further support to the aforementioned classification of the four clades. The N-terminal C2 domain prevalent in clade I and II (i.e., α, β, γ, ε, and δ) PLDs is a Ca^2+^-dependent phospholipid binding region critical for the affinity to substrates (Wang, 2005). The PX/PH domains specific to the clade III PLDs (PLDζs) are crucial to PLD phosphoinositide binding activity, subcellular distribution and intracellular trafficking (Morris, 2007). The HKD1 and HKD2 motifs can be packed against each other, forming the core structure essential for PLD catalysis (Li et al., 2020). Our work revealed that more than 82% of the PLDs identified in mosses, liverworts, clubmosses, and ferns contained certain clade-specific sequence similarities (the C2, PX/PH domains, or the signal peptide sequences), and over 95% shared the HKD1 or HKD2 motifs, implying high inter- and intra-clade genetic conservation. In addition, we found moss-specific intron removal in PX/PH (ζ) *PLDs*. Such intron losses may provide evolutionary advantages through expression regulation and functional differentiation (Park et al., 2010; Wang H. et al., 2014).

Identification of the drought, salt, low-temperature, wound, and anaerobic related *cis*-elements in the *PpPLD* promoter regions suggested that these genes are associated with *P. patens* response to various stresses. For example, the LTR *cis*-acting element was involved in low-temperature responsiveness (Brown et al., 2001). The wheat cytosolic glyceraldehyde-3-phosphate dehydrogenase 1 gene, which contained the LTR and MBS *cis*-acting elements in its promoters, showed an upregulated expression under salt stress (Fei et al., 2017). Our results also suggested that *PpPLDs* were likely related to light and phytohormone responses, and body development, through *cis*-elements such as Gap-box, ABRE, and CAT-box (Park et al., 1996; Choi et al., 2000).

RNA-seq based gene expression analysis revealed that most *PLDs* of mosses, clubmosses, ferns, and liverworts were expressed in transcriptome experiments. Among the *PLDs* of *P. patens* and *C. purpureus*, *PpPLD3* and *CpPLD6* (both from moss sub-clade A, *PLDαs*) exhibited the most abundant expression, suggesting that in the experimental studies, the *α PLDs* might provide important functions to these two closely related mosses. For *P. patens*, 12 of the 14 *PLDs* were expressed in different life cycle phases, implying a crucial role in plant growth and development. We also found that about half of the *P. patens PLD* genes responded to environmental stresses. In particular, four α *PLDs*, i.e., *PpPLD2*, *PpPLD6*, *PpPLD10,* and *PpPLD11*, were involved in biotic and abiotic stress responses. These findings are expected given that *PLDαs* are known to function in stress-induced signaling in angiosperms (Kargiotidou et al., 2010; Bourtsala et al., 2017; Ufer et al., 2017).

A previous adaptive evolution analysis suggested that positive selection drove the evolution of PX/PH and SP *PLDs* of *Arabidopsis*, rice, poplar, and grape (Liu et al., 2010). In the present study, we found evidence that most of the C2, PX/PH, and SP *PLDs* of mosses, clubmosses, liverworts, and ferns were subject to ongoing purifying selection, indicating the dominance of non-synonymous mutations during the evolution of early land plant PLD gene family. In addition, both relaxed and intensified selections were found in moss C2 *PLDs*. These results suggested that the C2 *PLDs* were particularly diversified and might be a potential source of new biological functions during moss evolution. In support of this conclusion, the moss C2 *PLDs* were found to display highly diverse gene structures and were the most differentially expressed.

Mosses are considered one of the earliest colonizers of land. During their transition from aquatic to terrestrial habitat, mosses had to tolerate biotic and abiotic stresses such as viruses, drought and salinity (Yue et al., 2012). Intriguingly, our results obtained through multiple methods suggested that several sites and/or branches of clade I–IV *PLDs* (C2, PX/PH, and SP *PLDs*) experienced an episode of positive selection in the moss lineage. Among them, the strongest positive selection signals were detected in the moss C2 *PLDs*, e.g., *P. patens PLD11* and *C. purpureus PLD2*. The predicted three-dimensional model of *PpPLD11* revealed that the 12 positively selected sites existed on the enzyme’s surface, suggesting that they may affect substrate binding ability. In angiosperms, C2 PLDs are implicated in response to diverse environmental stressors (Wang, 2005; Zhao, 2015). For example, overexpression of the heterologous C2 *PLDs* (*PLDαs*) in *Arabidopsis* and tobacco (*Nicotiana spp.*) improved the tolerance of these plants to drought and osmotic stress (Wang J. et al., 2014; Ji et al., 2017). We reason that the evolution of *PpPLDs* might confer beneficial traits particularly associated with stress regulatory networks, and thereby facilitated *P. patens* adaptation to terrestrial environments.

In summary, we revealed 84 *PLD* genes in six mosses, and investigated their evolutionary history. Conserved sequence characteristics (e.g., domain, motif, and gene structure), and expression of these *PLDs*, especially *P. patens PLDs* were examined. Although purifying selection largely drove the evolution of *PLDs* of mosses, liverworts, clubmosses, and ferns, episodic positive selection left footprints in the genetic diversity of moss *PLDs*. Our results will be informative for designing experiments to better understand the biological functions of *PLDs* in mosses. Future exploration of *PLDs* from more different plant groups and deciphering their functions may help dissect the evolution of PLD-mediated signaling in plants.

## Data Availability

The datasets presented in this study can be found in online repositories. The names of the repository/repositories and accession number(s) can be found in the article/Supplementary Material.
