# Supplementary figures and images for: Characterization and evolutionary diversification of the phospholipase D gene family in mosses
Source: Front Genet. 2022 Oct 13;13:1015393. doi: 10.3389/fgene.2022.1015393 (PMC9607936; doi:10.3389/fgene.2022.1015393)

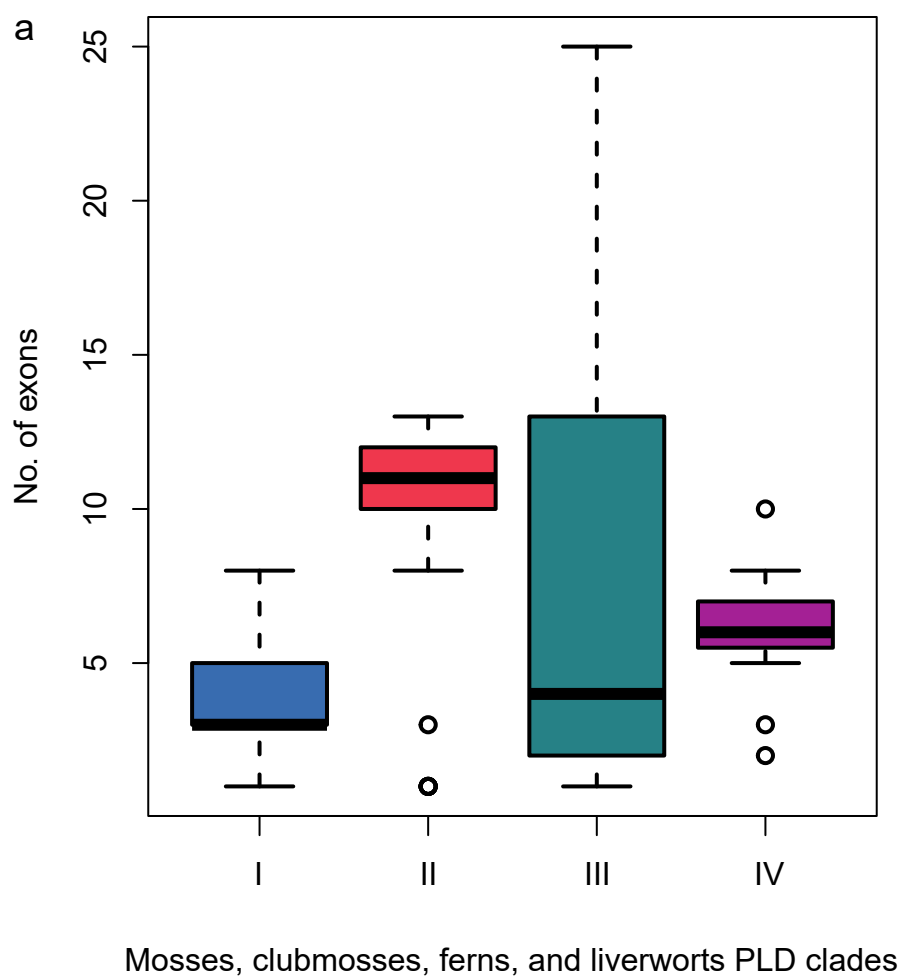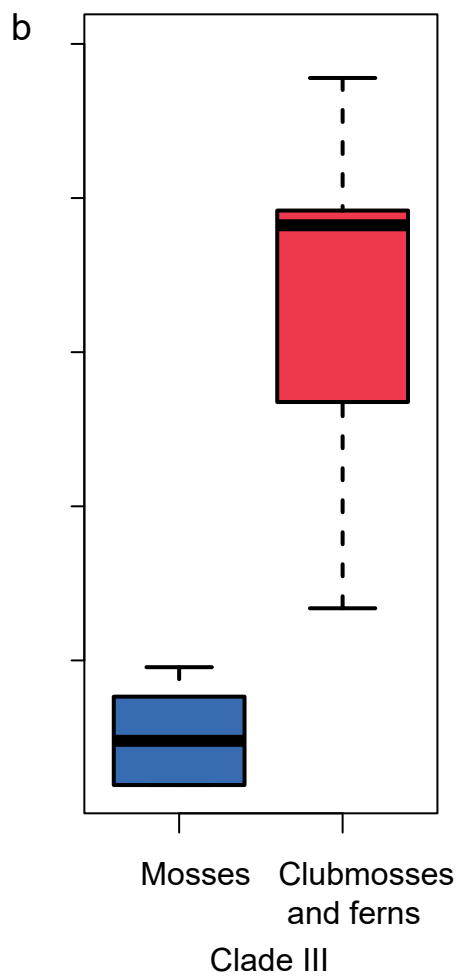

Supplement: Supplementary file 3 [file Image5.PDF]

**a**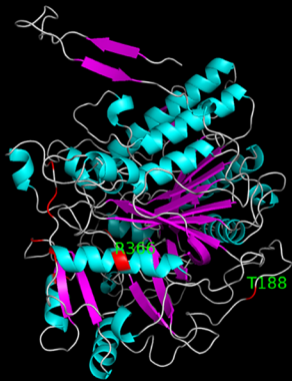**b**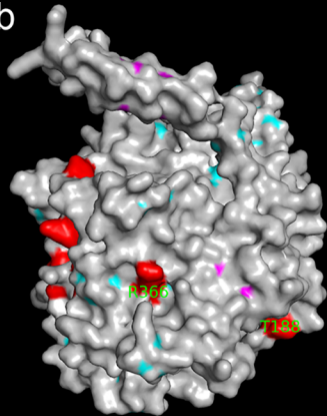**c**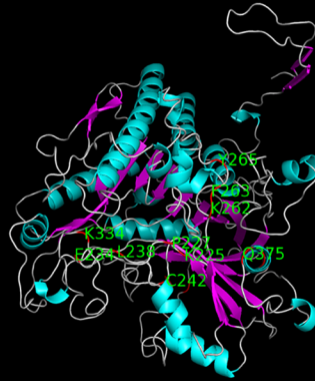**d**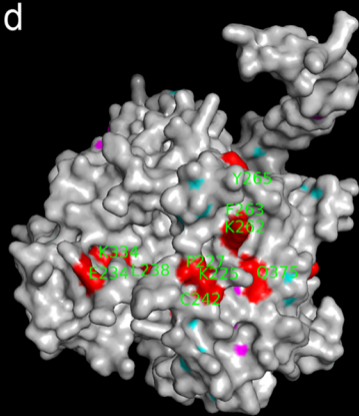

Supplement: Supplementary file 4 [file Image6.PDF]

# Clade I

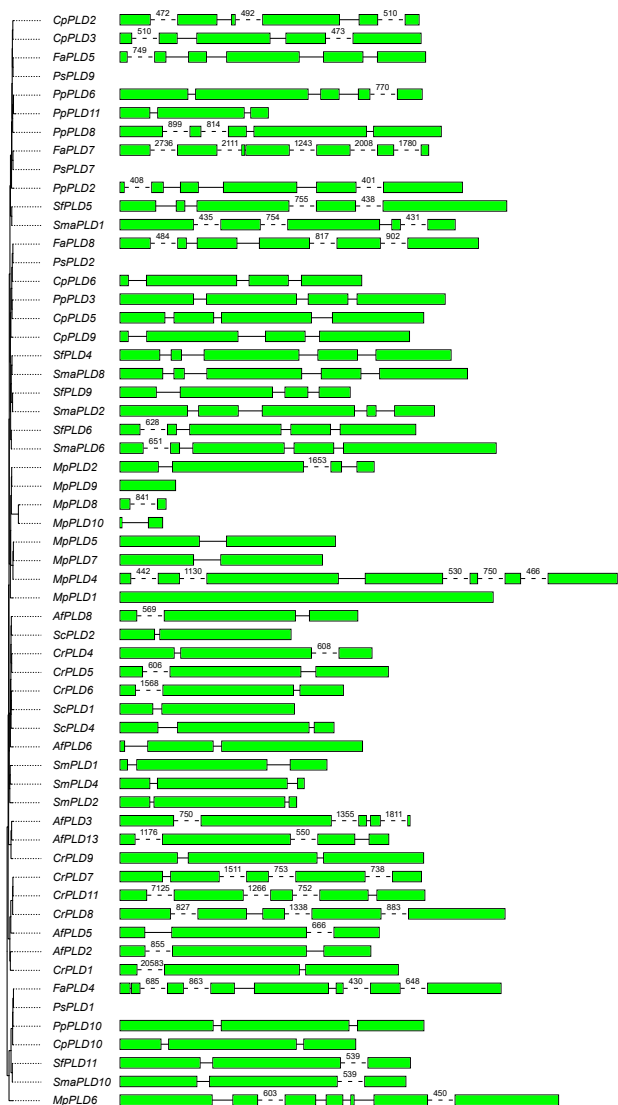

# Clade IV

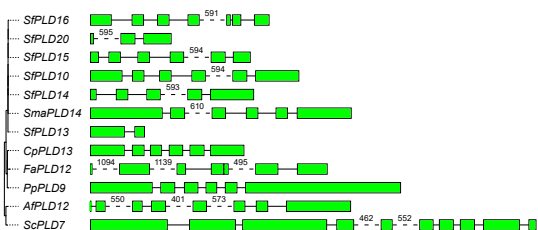

# Clade II

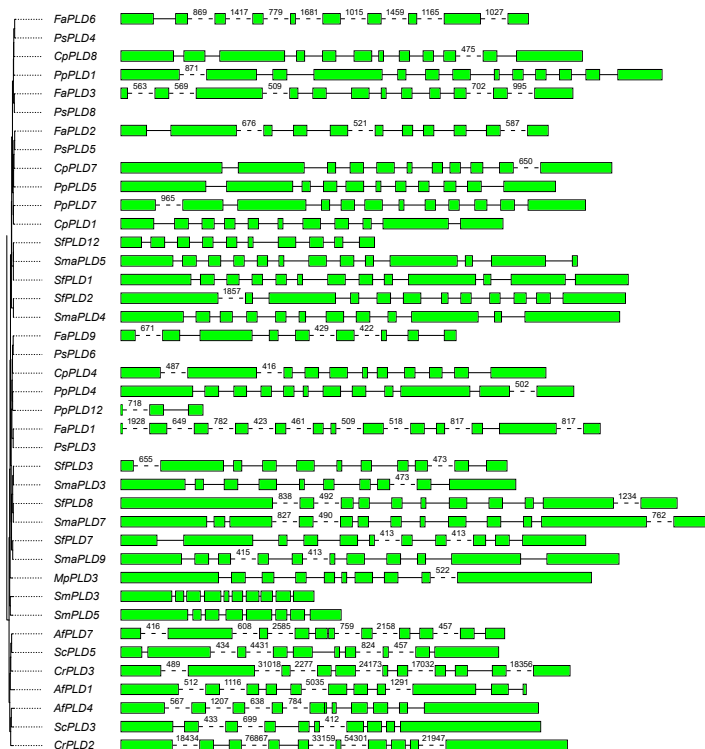

# Clade III

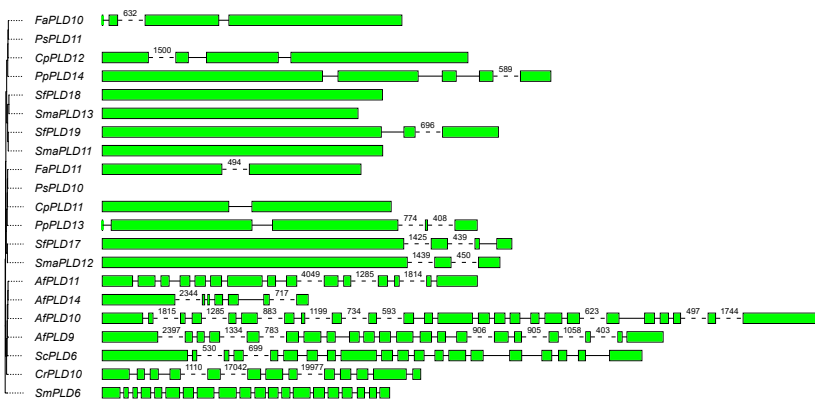

500 bp

Supplement: Supplementary file 5 [file Image4.PDF]

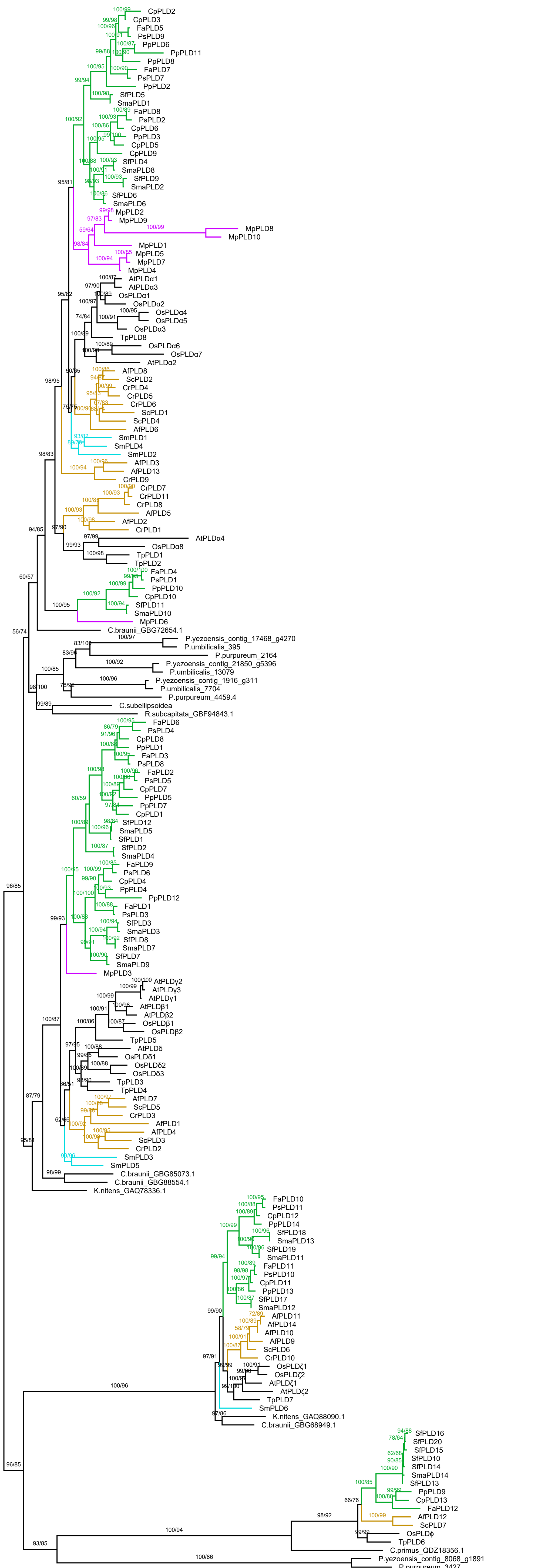

Supplement: Supplementary file 6 [file Image2.PDF]

*P. patens*

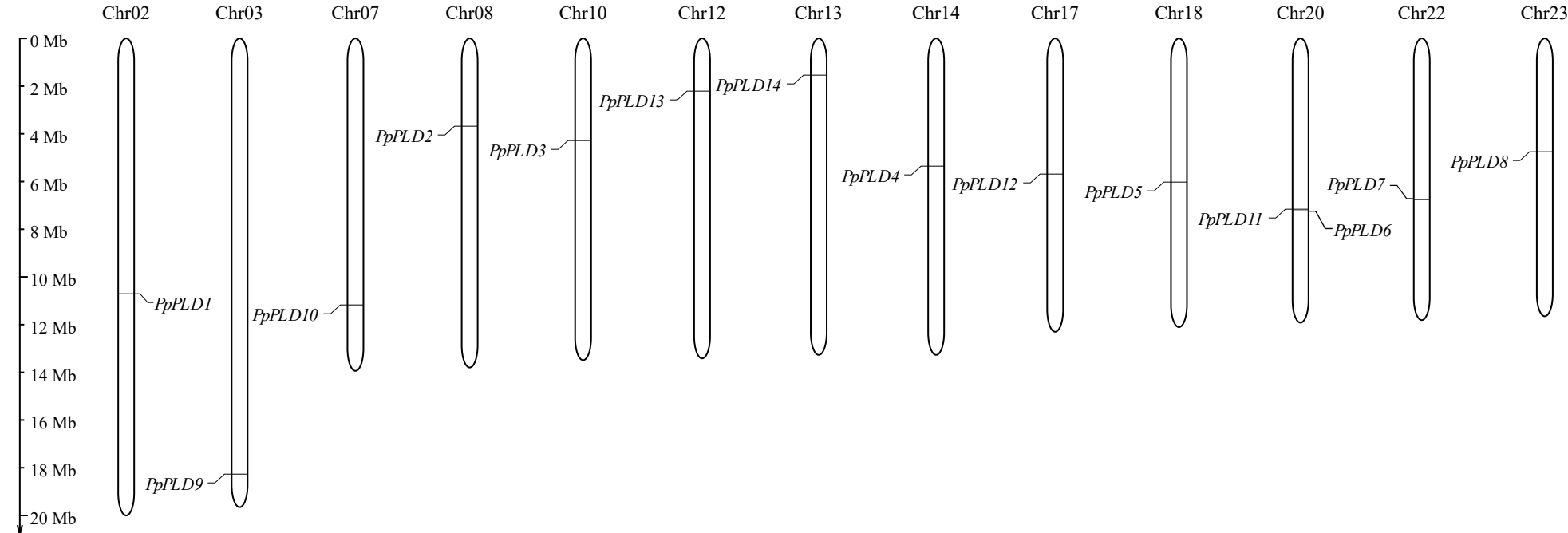

*S. fallax*

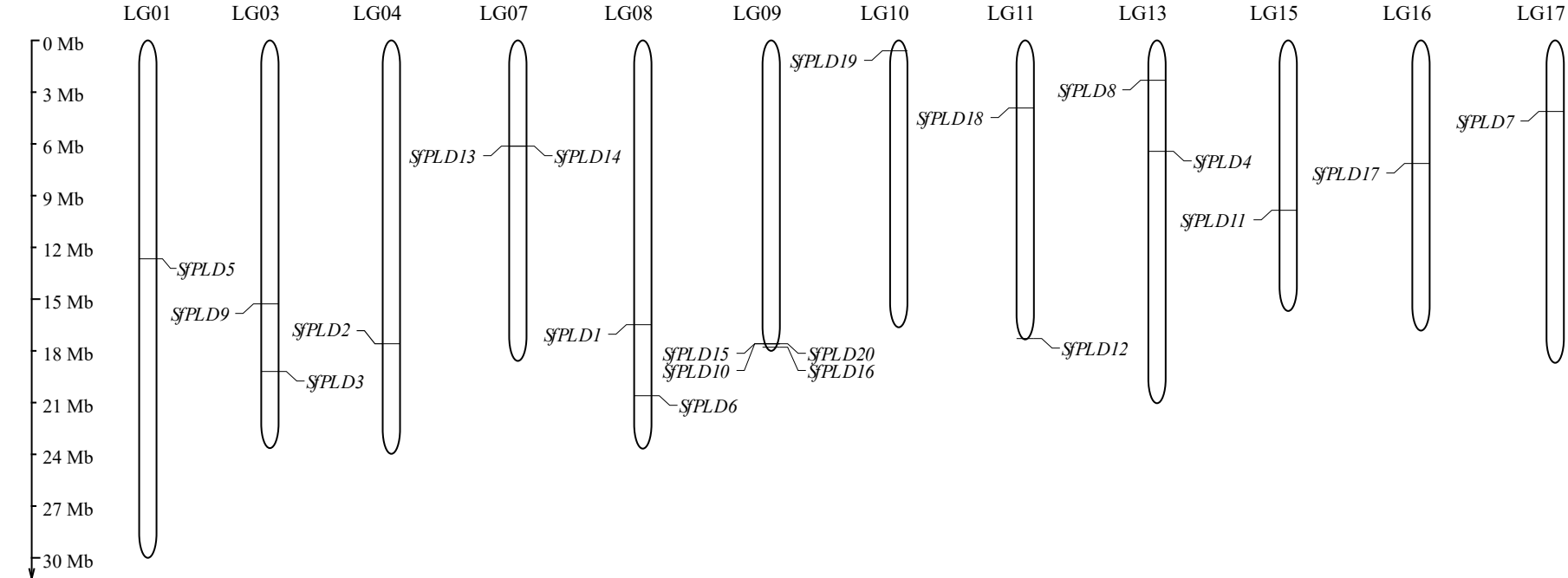

*S. magellanicum*

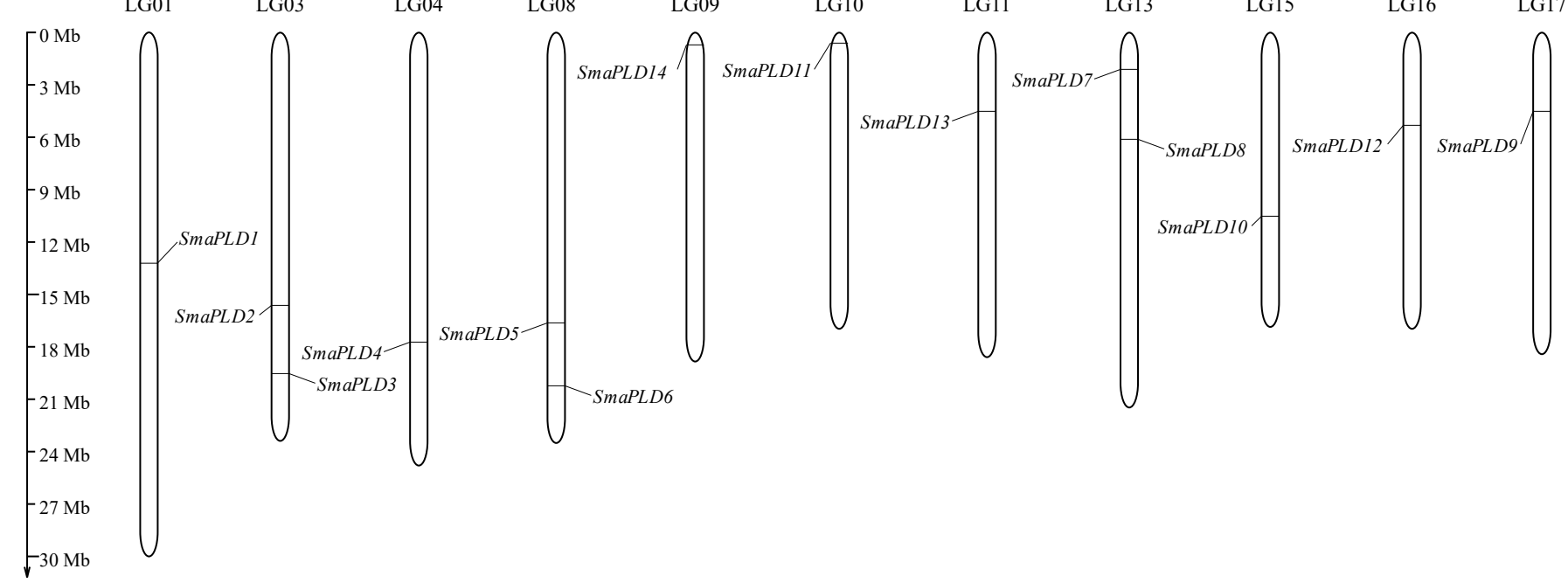

*C. richardii*

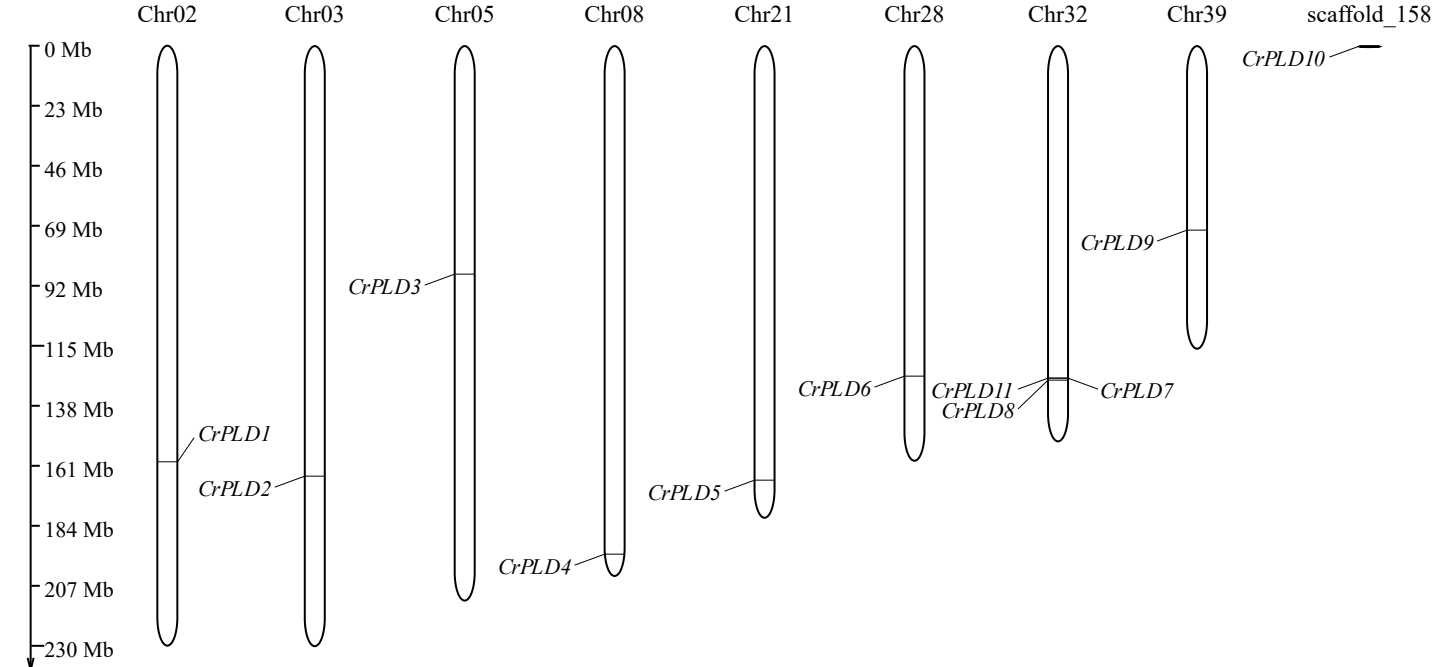

*C. purpureus*

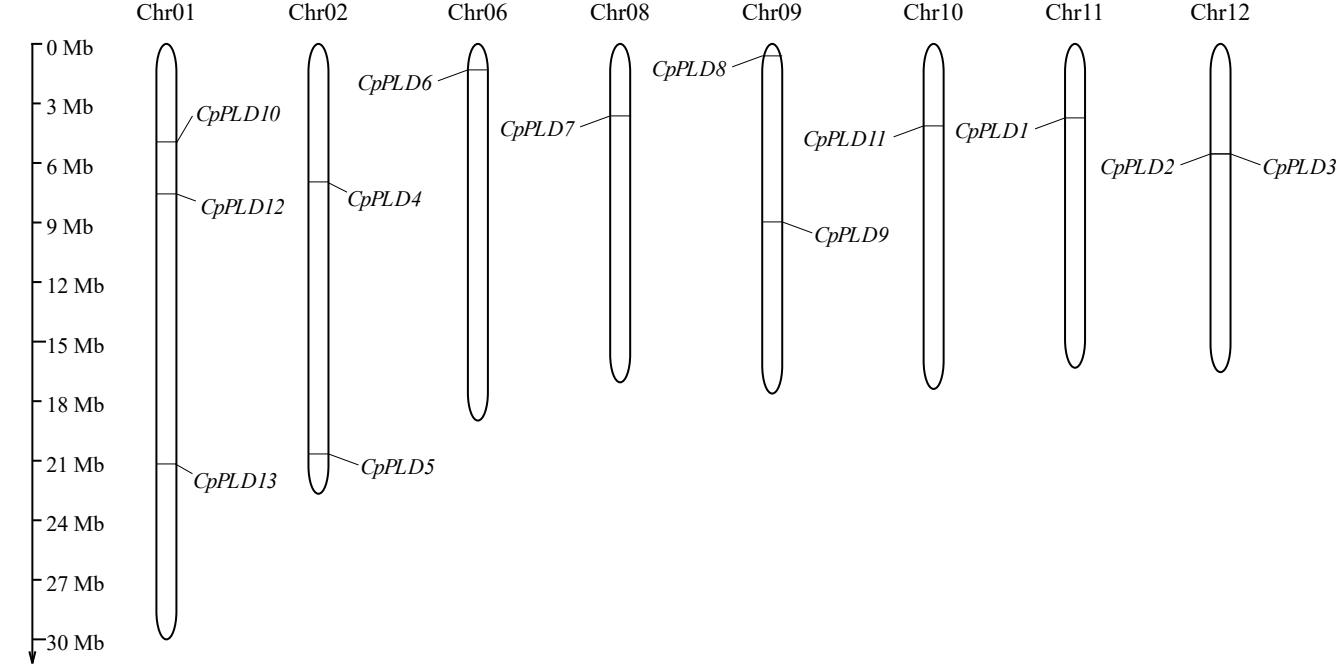

Supplement: Supplementary file 12 [file Image1.PDF]
