# Supplementary material for: Characterization and evolutionary diversification of the phospholipase D gene family in mosses
Source: Front Genet. 2022 Oct 13;13:1015393. doi: 10.3389/fgene.2022.1015393 (PMC9607936; doi:10.3389/fgene.2022.1015393)
Supplement: Supplementary file 7 [file Image3.PDF]

1 Alignment of the N terminal PLD domain of moss PLDs:

```
SfPLD10         -GVLHAKLWISNKK------------------HVYVGSAN-----
SmaPLD14        -GVLHAKLWISNKK------------------HAYVGSAN-----
SfPLD20         -GVLHAKLWISNKK------------------HAYVGSAN-----
SfPLD15         -GVLHAKLWISNKK------------------HAYVGSAN-----
SfPLD14         -GVLHAKLWISNKK------------------HAYVGSAN-----
PpPLD9          ---IHSKLWIANKE------------------DAYLGSAN-----
PpPLD1          -YSHHQKMTIVDT--GP---------HDRRTITSFIGGLDLTGGR
PpPLD2          -FTHHQKSLIVDAS-MPSDC----ITSQERRLVSFVGGLDLCDGR
PpPLD3          MFTHHQKTIIVDAP-LPGS------DTGRRRIMSFVGGIDLCDGR
PpPLD4          -YTHHQKTVIVDSQ-GP---------GNKRKLTSFLGGLDLCDGR
PpPLD5          --SHHQKTVIVDS--GP---------NEQRRLTSFIGGLDLTGGR
PpPLD6          -FTHHQKTVIVDAP-LPGAD----AFSSGRRLVSFVGGLDLCDGR
PpPLD7          -YSHHQKITIVDS--GP---------PKQRRLISFIGGLDLTGGR
PpPLD8          -FTHHQKTVIVDAA-LPGAN----PFSPGRRIISFVGGLDLCDGR
PpPLD10         -FTHHQKVVCMDAP-ALPS-----PHLAPRRVLAFQGGIDLCDGR
PpPLD11         --SHHQKTIIVDAE-RP---------GAKGRLVSFVGGLDLCDGR
PpPLD13         LWSHHEKLVIVDHH------------------ICFLGGLDLCFGR
PpPLD14         LWSHHEKLVIVDHH------------------VCFLGGLDLCYGR
SfPLD1          -YSHHQKVVIVDTD-GP---------SNMRKLTSFIGGLDLTGGR
SfPLD2          -YSHHQKLMIVDSS-GP---------RNMRKLTSFIGGLDLCDGR
SfPLD3          -YTHHEKMLIVDAP-SL---------GDQRIVTSFVGGLDLCDGR
SfPLD4          MFTHHQKTIIVDAPAVPGRYQGSATEQQQRRIVSFVGGIDLCDGR
SfPLD5          MFTHHQKTVITDAP-VSGPH---GYYTGQRRIVSFVGGIDLCDGR
SfPLD6          MFTHHQKTIIVDAA-VPGS------SSGQRRILSFVGGIDLCDGR
SfPLD7          -YTHHQKLIIMDTQ-GTG--------GNSRKLTSFIGGLDLCDGR
SfPLD8          -YTHHQKVLIVDTQ-GP---------GDRRIVTSFVGGLDLCDGR
SfPLD9          MFTHHQKLLVVDAP-VVTSG---YRQSEQRRIVSFVGGIDLCLGR
SfPLD11         -FTHHQKVVVMDYP-DLV------PDSNQRRLVAFQGGIDLCDGR
SfPLD12         -YSHHQKVVIVDTD-GP---------SNMRKLTSFIGGFDLTGGR
SfPLD17         LWSHHEKLIIVDHQ------------------VCFIGGLDLCFGR
SfPLD18         LWSHHEKLVIVDNY------------------VCFLGGLDMCFGR
SfPLD19         LWSHHEKLVIVDNY------------------VCFLGGLDMCFGR
CpPLD1          -YSHHQKITIVDTV-GP---------SGPRRLTSFIGGLDLTGGR
CpPLD2          -FTHHQKSVTVDAA-LPGAG----SDSAGRRIVSFVGGLDLCDGR
CpPLD3          -FTHHQKSVIVDAP-LPGAG----AYSPGRRIVSFVGGLDLCDGR
CpPLD4          -YTHHQKITIVDSQ-GP---------GNKRKLTSFIGGLDLCDGR
CpPLD5          MFTHHQKTIIVDAA-HPGA------DNGRRRIMSFVGGIDLCDGR
CpPLD6          MFTHHQKTIIVDAP-LPGA------DTGRRRIMSFVGGIDLCDGR
CpPLD7          -YTHHQKCVIVDS--GP---------YGQRRLSSFVGGLDLTAGR
CpPLD8          -YSHHQKMTIVDS--GP---------YDRRTVVSFIGGLDLTAGR
CpPLD9          MFTHHQKSVILDAP-LPGQG-----ESGQRRVMSFVGGIDLCDGR
CpPLD10         -FTHHQKVVCMDAP-ALPS-----SHPAPRRVLAFQGGIDLCDGR
CpPLD11         LWSHHEKFVIVDHH------------------VCFLGGLDLCFGR
CpPLD12         LWSHHEKLVIVDHH------------------VCFLGGLDLCYGR
SmaPLD1         MFTHHQKTVITDAP-VSGPH---GYYTGQRRIVSFVGGIDLCDGR
SmaPLD2         MFTHHQKLLVVDAP-VVTSG---YRQSEQRRVVSFVGGIDLCLGR
SmaPLD3         -YTHHEKMLIVDAP-SL---------GDQRIVTSFVGGLDLCDGR
SmaPLD4         -YSHHQKLMIVDSQ-GP---------RNMRKLTSFIGGLDLCDGR
SmaPLD5         -YSHHQKVVIVDTD-GP---------SNMRKLTSFIGGLDLTGGR
SmaPLD6         MFTHHQKSIIVDAA-VPGS------SSGQRRILSFVGGIDLCDGR
SmaPLD7         -YTHHQKVLIVDTQ-GP---------GDRRIVTSFVGGLDLCDGR
SmaPLD8         MFTHHQKTIMVDVPAVPGRYQGSASEQQQRRIVSFVGGIDLCDGR
SmaPLD9         -YTHHQKLVIMDTQ-GTG--------GSSRKLTSFIGGLDLCDGR
SmaPLD10        -FTHHQKVVVMDYP-DLV------PDSNQRRLVAFQGGIDLCDGR
SmaPLD11        LWSHHEKLVIVDNY------------------VCFLGGLDMCFGR
SmaPLD12        LWSHHEKLIIVDHQ------------------VCFIGGLDLCFGR
SmaPLD13        LWSHHEKLVIVDNY------------------VCFLGGLDMCFGR
FaPLD1          -YTHHQKLTILDTQ-GP---------VNKRQLVSFIGGLDLCEGR
FaPLD2          --THHQKSVIVDS--GP---------IGQRRLSSFIGGLDLTGGR
FaPLD3          -YTHHQKVTIVDS--GP---------EERKTLTSFIGGLDLTGGR
FaPLD4          -FTHHQKVVCMDAP-AL--------QSPQRRVLAFQGGIDLCDGR
FaPLD5          -FTHHQKSVIVDAP-APGG-------GHGRRIVSFVGGLDLCDGR
FaPLD6          -YTHHQKMTIVDS--GP---------KERRTVTSFIGGLDLTSGR
FaPLD7          LFTHHQKSVIVDAP-SGGG-------VAQRRIVSFVGGLDLCDGR
FaPLD8          MFTHHQKTIIVDAP-LPGA------DTGRRRIMSFVGGIDVCDGR
FaPLD9          -YTHHQKCVIVDTQ-GP---------GNKRKLTSFIGGLDLCDGR
FaPLD10         LWSHHEKLVIVDHR------------------VCFLGGLDLCYGR
FaPLD11         LWSHHEKFVIVDHH------------------VCFLGGLDLCFGR
```

```
PsPLD3          -YTHHQKITILDTQ-GP---------TNKRQLISFIGGLDLCEGR
PsPLD4          -YSHHQKMTIVDS--GP---------HERRTVISFIGGLDLTGGR
PsPLD5          --THHQKSVIVDS--GP---------LGQRRLSSFIGGLDLTAGR
PsPLD6          -YTHHQKCTIVDTQ-GP---------GNKRKLTSFIGGLDLCDGR
PsPLD7          LFTHHQKSVIVDAA-LPGS------AHQQRRIVSFVGGLDLCDGR
PsPLD8          -YSHHQKMTIVDS--GP---------NERKTITSFIGGLDLTGGR
PsPLD9          -FTHHQKSVIVDAP-EPGG-------AHGRRIVSFVGGLDLCDGR
PsPLD10         LWSHHEKFVIVDHH------------------VCFLGGLDLCFGR
PsPLD11         LWSHHEKLVIVDHH------------------VCFLGGLDLCYGR
                       * *    :                      : *. :
```

2 Alignment of the C-terminal PLD domain of moss PLDs:

```
CpPLD13         ---NHAKFVVSDVR-ANIGTSNLVWDY
SfPLD10         ---NHAKYVVSDVR-ANIGTSNLL---
FaPLD12         ---NHAKFAVSNVR-AHIGTSNLVWDY
SmaPLD14        ---NHAKYVVSDVR-ANIGTSNLL---
SfPLD16         ---NHAKYVVSDVR-ANIGTSNLLW--
SfPLD13         ---NHAKYVVSDVR-ANIGTSNLLW--
SfPLD10         -GVLHAKLWISNKKHVYVGSAN-----
PpPLD9          ---NHAKFAVSDVR-ANIGTSNLVWDY
PpPLD1          MIYVHSKGMIVDDEYVISGSANINQRS
PpPLD2          -IYVHSKMMIVDDEYIIVGSANINERS
PpPLD3          -IYVHSKFMIVDDEYTIIGSANINQRS
PpPLD4          -IYVHAKGMVVDDEYIICGSANINQRS
PpPLD5          MIYVHAKGMIVDDELVILGSANINQRS
PpPLD6          -IYVHSKMMIVDDEYIIVGSANINERS
PpPLD7          MIYVHAKGIIVDDELVIMGSANINQRS
PpPLD8          ---VHSKMMIVDDEYIIVGSANINERS
PpPLD10         -IYCHAKIMVVDDEYLIIGSANINQRS
PpPLD11         -IYVHSKMMIVDDEYIIVGSANINERS
PpPLD12         -IYVHAKGMIVDDEYIICGSVSINQRS
PpPLD13         -IYVHSKIMIVDDWAVLIGSANINDRS
PpPLD14         -IYVHSKLMIVDDRLAIIGSANLNDRS
SfPLD1          -IYVHAKGMIVDDEFVIVGSANINQRS
SfPLD2          -IYVHSKGMIVDDELVILGSANINQRS
SfPLD3          -IYVHSKGMIVDDEYIICGSANINQRS
SfPLD4          -IYVHAKMMIVDDEYIMIGSANINQRS
SfPLD5          -IYVHAKMIIVDDEYIIVGSANINQRS
SfPLD6          -IYVHAKMMIVDDEYIIIGSANINQRS
SfPLD7          -IYVHAKGMVVDDEYVICGSANINQRS
SfPLD8          -IYVHSKGIIVDDEYIICGSANINQRS
SfPLD11         -IYCHAKMMVVDDEYVIIGSANINQRS
SfPLD12         -IYVHAKGMVVDDEFVIVGSANINQRS
SfPLD17         -IYVHSKIMIVDDRTVLIGSANINDRS
SfPLD18         ----HSKLMIVDDRFVLTGSANINDRS
SfPLD19         ----HSKLMIVDDRFVLIGSANINDRS
CpPLD1          MIYVHAKGMIVDDELVIMGSANINQRS
CpPLD2          -IYVHSKVMIVDDEYIILGSANINERS
CpPLD3          -IYVHSKMMIVDDEYIIVGSANINERS
CpPLD4          -IYVHAKGMVVDDEYIICGSANINQRS
CpPLD5          -IYVHSKFMIVDDEYTIIGSANINQRS
CpPLD6          -IYVHAKMLIADDEYIIIGSANINQRS
CpPLD7          MIYVHAKGMVVDDELVIMGSANINQRS
CpPLD8          MIYVHSKGMIVDDEYVISGSANINQRS
CpPLD9          -IYVHAKMMIVDDEYIILGSANINQRS
CpPLD10         -IYCHAKMMVVDDEYLIIGSANINQRS
CpPLD11         -IYVHSKIMIVDDRSVLIGSANINDRS
CpPLD12         ----HSKLMIVDDRFVLIGSANINDRS
SmaPLD1         -IYVHAKMMIVDDEYIIVGSANINQRS
SmaPLD2         -IYVHAKMMIVDDEYIIIGSANINQRS
SmaPLD3         -IYVHSKGMIVDDEYIICGSANINQRS
SmaPLD4         -IYVHSKGMIVDDELVILGSANINQRS
SmaPLD5         -IYVHAKGMIVDDEFVIVGSANINQRS
SmaPLD6         -IYVHAKMMIVDDEYIIIGSANINQRS
SmaPLD7         -IYVHSKGIIVDDEYIICGSANINQRS
SmaPLD8         -IYVHAKMMIVDDEYIMIGSANINQRS
SmaPLD9         -IYVHAKGMVVDDEYVICGSANINQRS
SmaPLD10        -IYCHAKMMVVDDEYVIIGSANINQRS
SmaPLD11        ----HSKLMIVDDRFVLIGSANINDRS
SmaPLD12        -IYVHSKIMIVDDRTVLIGSANINDRS
SmaPLD13        ----HSKLMIVDDRFVLTGSANINDRS
FaPLD1          -IYVHAKGMVVDDEYLICGSANINQRS
FaPLD2          -VYVHSKGMIVDDEFVIVGSANINQRS
FaPLD3          MIYVHSKGMIVDDEYVISGSANINQRS
FaPLD4          -IYCHSKIMVVDDEYLIIGSANINQRS
FaPLD5          -IYVHSKMMIVDDEFIIVGSANINERS
FaPLD6          MIYVHSKGMIVDDEYVISGSANINQRS
FaPLD7          ---VHSKMMIVDDEYIIVGSANINERS
FaPLD8          ------------DEYTIIGSANINQRS
FaPLD10         ----HSKLMIVDDRFVVIGSANINDRS
```

```
FaPLD11         -IYVHSKVMIVDDWSVLIGSANINDRS
PsPLD1          -IYCHSKIMVVDDEYLIIGSANINQRS
PsPLD2          -IYVHSKMLIADDEYTIIGSANINQRS
PsPLD8          MIYVHSKGMIVDDEYVISGSANINQRS
PsPLD9          -IYVHSKMMIVDDEFIIVGSANXNERS
PsPLD10         -IYVHSKVMIVDDWSVLIGSANINDRS
PsPLD11         ----HSKLMIVDDRFVLIGSANINDRS
                                  *:  .
```

3 Alignment of C2 domain of moss *PLDs:*

```
PpPLD1          TLEVKIFEAVQLPNL-DGFSQKLSDFTSG-L----SIFQKSKHK-----------DEPS
PpPLD2          -LLIKIYGAEEIVTQ-DRKTGKAPGFIRM-VV---QTSENLLG-------------FGK-
PpPLD3          TLHVTIYGCKGIASP-VHHGGGFSSFFKS-IV---GAAQDAIS-----------------
PpPLD4          TLEVEIRSAENLPNM-DMFSEKFRQCFSY-LTICKAPFVKTKSKINEK--------GHGH
PpPLD5          SLEVTIFEAVNLPNM-DMFSEKVRRFAHNNLP---SSLEKLKKT-----------AHLH
PpPLD6          --LVTIYDGKNLETE-ERKSGKAPGFLRK-LV---ETSEEVLK------------MGR-
PpPLD7          TLEVFMHQAVNLPNM-DMFSEKIRQLTQN-LP---GPLEKLKKV-----------AKLH
PpPLD8          TLYVHIHDAQNIASG-EHKASRTPGFFKK-LI---ETSEMI--------------LGR-
PpPLD10         ------------------------------------------------------------
SfPLD1          TLDVVLYAAVNLPNM-DIFSEKIRQATSS-LP---SILQRAKSK-----------AKEA
SfPLD2          TLDVMLYEAVNLPNM-DLFSEKLRQFVSN-LP---TKIQKVKAK-----------ASEH
SfPLD3          TLEVWLYEAKNMPNM-DLFSERCRRCFSC-LSVCKPPFVKAKAKVKKH--------SHGH
SfPLD4          VLDVTIYEAENLVNE-KRSTGSAPVFLRK-LL---EGGEEIVG------------IGK-
SfPLD5          TLHVSIYKAEGLINE-DRLTGGAPKLFRQ-FI---EGSEAALG------------LGK-
SfPLD6          TLHVTIYEAENIINT-ERATGGAPAFFRR-FL---ESSQEVVG------------IGK-
SfPLD7          TLNVWLHEARRLPNM-DLTSERLRQCFSF-LPSCKTPFVRMKNKAKEH--------THGH
SfPLD8          TLEVWLYEAKSLPNM-DLFSERLRQCFSF-LSVCKAPFVKAKAKVKEH--------SHGH
SfPLD9          TLDATIYQAENLINV-ERSTGNAPALFRQ-FV---EAAESLVG------------IGK-
CpPLD1          TLEVGIFQAVNLPNM-DMFSEKVRQYTSK----------LKEA------------TGMG
CpPLD2          ILDVKIHDAKNLMNQ-ERKSG----FFRK-VV---EKPEVVRRN-----------MGK-
CpPLD3          TLLVKIHDAENLMNQ-ERKSGKAPGFLRK-LI---ETSEEVLR------------MGK-
CpPLD4          TLEVWLKESTNLPNM-DMFSEKFRQCFSY-LTICKAPCVKAKTKAE----------SHGH
CpPLD5          TLHVTIFGGRGIAAP-ERSGGGPKRFFHS-LI---GAATDAIS-----------------
CpPLD6          TLHVTIYGGRGIATPQDKKQHKISRFFTS-FL---ENAQDAIM-----------------
CpPLD7          TLEASVYEAVSLPNM-DMFSERVRLFANN-LP---GPLEKLKKA-----------TGVH
CpPLD8          TLEVYIDKAARLPNM-DVFSQKISDFASG-L----AIFQKSKSK-----------TKLS
CpPLD9          -LHATIYEGRHMTS--ERRTGGAPAFFRK-IV---EGAEDVLS------------LGK-
SmaPLD1         TLHVSIYKAEGLINE-DRLTGGAPKFFRQ-FI---EGSEAALG------------LGK-
SmaPLD2         TLDATIYEAENLINV-ERSTGNAPAQFRQ-FV---EGAESLVG------------IGK-
SmaPLD3         TLEVWLYEAKNMPNM-DLFSERCRRCFSC-LSVCKPPFVKAKAKVKKH--------SHGH
SmaPLD4         TLDVMLYEAVNLPNM-DLFSEKLRQFVSN-LP---TKIQKVKAK-----------ASEH
SmaPLD5         TLDVVLYEAVNLPNK-DIFSEKIRQATSS-LP---SILQRAKSK-----------AKEA
SmaPLD6         TLHVTIYEAQNIINT-ERTTGGAPAIFRR-FV---ESSQEIIG------------IGK-
SmaPLD7         TLEVWLYEAKSLPNM-DLFSERLRQCFSF-LSVCKAPFVKAKAKVKEH--------SHGH
SmaPLD8         VLDVTIYEAKNLVNE-ERSTGRAPVFFRK-LL---EGAEEIVG------------IGK-
SmaPLD9         TLNVWLYEARRLPNM-DLTSERLRQCFSF-LPSCKTPFVRMKNKAKEH--------THGH
FaPLD1          TLEVHIWKARELPNM-DTVSEKFRQLFSC-VTVCKTALEPKKETKE----------GHHH
FaPLD2          TLEVSIHEAVSLPNM-DMFSEKLRLFAHNYLP---GPLEKLKGV-----------TRGH
FaPLD3          TLEVTIFQAVRLPNL-DGLSQKFSTLTSN-LSI---------------------------
FaPLD5          TLLVTIHDAQSIVNE-ERKSGKAPSFFRK-LV---ETSEEVLK------------MGK-
FaPLD7          TLVVTIHDAENIVTQ-ERKTGSAPAFFRK-II---ETSEEILR------------MGK-
FaPLD8          TLHVTIYEGRGMANPSEKKSHNVGHFFKS-LL---DTAQGAIM-----------------
FaPLD9          TLEVHLKEAKNLPNM-DMFSEKFRQFFSY-LTVCKTPFVKAKTKAEEK--------AHGH
PsPLD5          TLEVSIHEAVSLPNM-DMFSEKIRLFTHKNLP---GPLEKLKSA-----------ARVH
PsPLD6          TLEVHLKEARNLPNM-DMFSEKFRQIFSY-LTVCKAPFVKAKDKVEEKELGDGKKLGHVH
PsPLD7          TLVVTIHDAENIVTQ-ERKTGSAPAFFRK-II---ETSEEILR------------MGK-
PsPLD9          TLLVTIHDAQKIVNE-ERKSGKAPSFFRK-LV---ETSEEVLK------------MGK-


PpPLD1          APNVPHITSDPYVTVVLAGARVARTRVISN---DVNPKWHESFSIPVAHYVDHIVFRVKD
PpPLD2          ------GYSQYYATVDLGKTRVGRTRVLEG--NFKDPEWNETFSIFCAHTVSHLVVSIKD
PpPLD3          ------GDDEYYATVDLGTTRVGRTRVLKE--STSEPVWNESFRIYCCHSVPDLTISVKD
PpPLD4          RPK--GITSDPYAAVNLAGARVARTRVISN---STNPQWNEHFSIPVAHYVSEVEITVKD
PpPLD5          GPST-VITSDPYTVVVLAGARVARTRVINN---DSNPKWNEHFLVPVAHQICNIVFVVKD
PpPLD6          ------GPSQYYATVDLGETRVGRTRVLAGSKDPKDPVWNEKFRIYCAHTISHVIVSIKD
PpPLD7          SAT--VITSDPYAIVVLAGAKVARTRVISN---NANPEWKERFIIPVAHFVHEIVFKVKD
PpPLD8          ------GPPQYYATVDLGPTRVGRTRVLATSKDLKDPVWNETFHIYCAHTVSQVVVSIKD
PpPLD10         ------------------PVRVARTRAVKR---LKRVKFEESFRVFCAHLVASINVFVYS
SfPLD1          AANVTGITSDPYVIVVLAGARVARTRVISN---NVNPKWDEHFSVPVAHYVYEVVFTIKD
SfPLD2          NVK--VITSDPYAIVILAGARVARTRVINN---NVNPKWNEHFVVPVAHFVYDVQITLKD
SfPLD3          RPK--GITSDPYAYVVLAGAKVASTRLIIN---NANPRWNEHFQIPVAHFTNEILIVIKD
SfPLD4          ------GYSQYYATVDLVPARVGRTRVLKG--EPSDPVWNESFRIYCAHTVPDVQISIKD
SfPLD5          ------GFSQYYATVDLGIARVGHTSILKG--EPRDPVWNESFHFYCAHTVSDVSILVKD
SfPLD6          ------GASQYYATVDLGAARVGRTRVLKG--EPVDPVWNESFRIYCAHSVADVQISIKD
SfPLD7          RPK--GITSDPYASVVLAGARVARTRVISN---NASPTWDEHFKIPVAHYVDAVEISIKD
SfPLD8          HPK--GITSDPYASVMLAGAKVASTRVISN---NASPHWNEHFWIPVAHFTNEILIVIKD
SfPLD9          ------GYSQYYATVDLVPARVGRTRVLKG--QVADPVWDESFKIYCAHTVADVQISIKD
CpPLD1          APT--TITSDPYTTVVLGDARVARTRVISN---NVNPVWKEHFQIPVAHHVRDIVLEVKD
```

```
CpPLD2          -----VDKSQYYATVDLGETRVGRARVLEG--EPKDPVWNETFRIYCAHTVSHVVVSIND
CpPLD3          ------GQSQYYATVDLGETRVGRTRVLEG--EPKDPVWNETFRIYCAHTVSQVVVSIKD
CpPLD4          RPK--VITSDPYAAVNLAGARVARTRVISN---NTNPKWDEYFSIPVAHYVNDVEITVKD
CpPLD5          ------GDSKYYAVVDLDSTRVGRTRVLCE--SGGEPQWNESFRIYCCHNVSELTISVKD
CpPLD6          ------GDSEYYATVDLESSRVGRTRVLKN--HTGEPLWNESFRIYCCHTVSELTVSIKD
CpPLD7          GPTS-VITSDPYTVVVVAGARVARTRVISN---NANPKWNEHFLVPVAHHTFDIVFVVKD
CpPLD8          SPNV-RITSDPYVTVVVQDARVARTRVISN---SVNPEWREHFSIPVAHYVNDIVFTVKD
CpPLD9          ------GVSKYYATVDLTPARVGRTGIVKG--QPKTPVWNESFRIYCCHSVASVTVSVKD
SmaPLD1         ------GFSQYYATVDLGTARVGRTSILKG--EPRDPVWNESFQIYCAHTVSDVTILVKD
SmaPLD2         ------GYSQYYATVDLVPARVGRTRVLKG--QVADPVWDESFKIYCAHTVADVQISIKD
SmaPLD3         RPK--GITSDPYAYVVIAGAKVASTRVIIN---NANPRWNEHFQIPVAHFTNEILIVIKD
SmaPLD4         NVK--VITSDPYAIVILAGARVARTRVINN---NVNPKWNEHFVVPVAHFVYDVQITLKD
SmaPLD5         AASVTGITSDPYVIVVLAGARVARTRVISN---NVNPKWNEHFSVPVAHHVYEVVFTIKD
SmaPLD6         ------GASQYYATVDLGAARVGRTQVLKG--EPVDPVWNESFRIYCAHSVTDVQISIKD
SmaPLD7         HPK--GITSDPYASVVLAGAKVASTRVISN---NASPHWNEHFWIPVAHFTNEILIVIKD
SmaPLD8         ------GYSQYYATVDLVPARVGRTRVLKG--EPSDPVWNESFRIYCAHTVPDVQISIKD
SmaPLD9         HPK--GITSDPYASVVLASARVARTRVISN---NASPTWNEHFKIPVAHYVDAVEISIKD
FaPLD1          RPK--VITSDPYAAVNLAGARVARTRVISN---HTDPIWDEHFSIPVAHYVDDVRITVKD
FaPLD2          GASA-VITSDPYTVVVIAEARVARTRVINN---DANPKWKEHFHIPVAHHCFDVRCVVKD
FaPLD3          TPKT-PVHSDPYVTVVLANARVARTRVINN---DANPKWEEHFSIPLAHPVHDIVFTLRD
FaPLD5          ------GPSQYYATVDLGETRVGRTRILQG--EPKDPVWNETFRIYCAHTVSQVVVSIKD
FaPLD7          ------GFSQYYATVDLGKTRVGRTRVLEG--QPKDPVWNESFRIYCAHTVSELTVSIKD
FaPLD8          ------GDSEYYAAVDLESSRVGRTRILKE--HKGDPVWNESFRIYCCHTVSDLTISVKD
FaPLD9          RPK--GITSDPYAAVNLAGARVVRTRVISN---DTNPCWDEHFSIPVAHFVKEVQITVKD
PsPLD5          GPSS-VITSDPYTVVVIAKARVARTRVINN---DANPKWKEHFLIPVAHHCFDVRCVVKD
PsPLD6          RPK--GITSDPYAAVNLAGARLARTRVISN---ETDPNWDEHFSIPVAHYVKEVQITVKD
PsPLD7          ------GFSQYYATVDLGKTRVGRTRVLEG--QPKDPVWNETFRIYCAHMVSELTVSIKD
PsPLD9          ------GPSQYYATVDLGETRVGRTRILQG--EPKDPVWNETFRIYCAHTVSQVVVSIKD
                                    ::  :  :          : * * .  .*    :   : .

PpPLD1          QDM-LGTQKIGDVKIPV-EQVLHGSIVSGWFD-
PpPLD2          AAV-VGTAVIGRARIPA-IDLLSGKQIEDWYPL
PpPLD3          GAI-VGTVVIGRAKIPA-ESLLSGEPVEDWYQL
PpPLD4          NDV-LGAQLIGDVKIPV-GDIMDGKVVEGWHD-
PpPLD5          QDV-MGSEYIGEVRIPA-WLVINGGVVNDWFDL
PpPLD6          AAI-VGTTVVGRAKVPV-LDLLSGEEVDKEYQL
PpPLD7          QDV-VNSQFIGHVKIPV-EVVLNGGVVDNWFDL
PpPLD8          ASI-VGTTVVGRAKLPV-IELLSGQKIDRQYEL
PpPLD10         -----GAVYMGTAKIPVTEELLKGKTIDGWFEL
SfPLD1          QDV-LGSQHIGDVKIPV-EQLLDGDIIDDWFE-
SfPLD2          QDI-LGSKIIGDVKIPI-EQVLNGDTVEGWYDI
SfPLD3          EDM-VGAQHIGEVRIPA-EEVLKKQVVEGWYD-
SfPLD4          DAI-VGTAVVGRAKVPA-ADLLSGALVDGWYPL
SfPLD5          AAI-IGTAVVGRAKVPA-FELLSGRKIDDWYQL
SfPLD6          AAI-LGSALIGRAKVPA-EELLSESVVEDWYPL
SfPLD7          DDM-LGAQYIGSVTIPV-EQVIDGQVIEGWHD-
SfPLD8          DDM-LGAQYIGEVMIPA-EEVLEKQVVEGWYD-
SfPLD9          DAV-VGSAVIGRAKVPA-AELLSGAKLDDWYPL
CpPLD1          QDV-MGSQFIGKVSISA-GSVLNGGVVDGWFPL
CpPLD2          AAIFVGTTVVGRVKVPV-IDLLSGRKIESSYPL
CpPLD3          AAI-VGTTVVGRVKVPV-IDLLSGRKIESSYPL
CpPLD4          NDM-FGAQLIGSVTIPV-DKVIHGEVVEGWHDI
CpPLD5          GAI-IGTVVIGRAKVPT-ESLLSGEPIDDWYQL
CpPLD6          AAV-IGTVVVGRAKIPA-ETLLSGSPVDDWYKL
CpPLD7          QDV-MGSQRIGQVKISA-GPLLNGGVVDGWYDL
CpPLD8          QDV-LGTQHMGDVKISV-EKVLNGGVVNGWFD-
CpPLD9          AAI-LGTAVIGRADVPA-QQLLNGEPVEDWYPL
SmaPLD1         AAI-IGTAVVGRAKVPA-FELLSGRKIDDWY-Y
SmaPLD2         DAV-VGSAVIGRAKVPA-AELLSGAKLDDWYPL
SmaPLD3         EDM-VGAQHIGEVRIPA-EEVLKKQVVEGWYD-
SmaPLD4         QDI-LGSKIIGDVKIPI-EQVLNGDTVEGWYDI
SmaPLD5         QDV-LGSQHIGDIKIPV-EQLLGGDIIDDWFE-
SmaPLD6         AAI-LGSALIGRAKVPA-EELLSESVVEDWYPL
SmaPLD7         DDM-LGAQYIGEVMIPA-EEVLEKQVVEGWYD-
SmaPLD8         DAI-LGTAVVGRAKVPA-ADLLSGALVDGWYPL
SmaPLD9         DDM-LGAQYIGSVTIPV-ERVIDGQVIEGWHD-
FaPLD1          NDV-MGAQLIGDAIVPV-DKILDGESVEGWHD-
FaPLD2          QDV-MGSQYIGEVSIAA-RVLLHGDVVDGWFDL
FaPLD3          QDV-IGTDHIGDVKIPV-ERVLNGGVVHGWFD-
```

```
FaPLD5          AAI-VGTTVVGRARVPV-IDLLSGNRIDESYPI
FaPLD7          AAI-MGTVVVGRAK-------------------
FaPLD8          AAV-IGTVVVGRAKLSA-ETLLSGEPVEDWYQL
FaPLD9          NDV-FGAQMIGDVNIPV-EDVIRGQQIEGWYDI
PsPLD5          QDV-MGSQYIGEVSIAA-KLLLHGGVVDGWFDL
PsPLD6          NDM-LGAQLIGDVNIPV-ERLIHGENIEGWHDI
PsPLD7          AAI------------------------------
PsPLD9          AAI-VGTTVVGRARVPV-IDLLSGRQS------
```

4 Alignment of PX domain of moss *PLDs:*

```
PpPLD13         ------------------------------------------------------------
PpPLD14         ------------------------------------------------------------
SfPLD17         ------------------SIQLEYQGFKWRLERKAAQVFFLHLALKRRALLQDLQEKQEQ
SfPLD18         EVSQSKRNDMSLASQLVYTIECKYRQFTWQLKRRAAEVLLLHLALKKRALLEELQEKQEL
SfPLD19         ----------------VYTIECTYRQFTWRLERQAAEVILLHLFLKKRALLEELQEKQEL
CpPLD11         -VSRSE----GTAFQLIYTIDFEYRQFKWRLVKKATQVFSLHSAIKFRALVEDLHEKQEQ
CpPLD12         ------------------------------------------------------------
SmaPLD11        ----------------VYTIECTYRQFTWRLERRAAEVILLHLFLKKRALLEELQEKQEL
SmaPLD12        -----------------YSIQLEYQGFKWRLERKAAQVFFLHLALKRRALLQDLQEKQEQ
SmaPLD13        EVSQSKRNDMSLASQLVYTIECKYRQFTWQLKRRAAEVLLLHLALKKRALLEELQEKKEL
FaPLD11         -VSRSE----GTALQLIYTIDFEYRQFKWRLVKKATQVFSLHSAIKFRALVEDLHEKQEQ
PsPLD10         -----------TALQLIYTIDFEYRQFKWRLVKKATQVFSLHSAIKFRALVEDLHEKQEQ
PsPLD11         ------------------------------------------------------------


PpPLD13         ------------------------------------------------------------
PpPLD14         ------------------------------------------------------------
SfPLD17         VKEWVQSLGLGEHSPSGVNLHLH-------DDYDQEDNPPAPRTD-IPSSAALPVMRPAL
SfPLD18         LKEWMHSLGLGDEQ-LGINVALQLQVSD--EEGTDEISQFSSKTG-IPARAVLPVMRPAF
SfPLD19         VKEWVNNLRLGDQQ-SDINLSLHPQRSSGGEQGGDENNQSSKRGDHIPSSAVFPVIRPAF
CpPLD11         AREWFQNLGIGDH--TGINLHAQ-------EEEEQDENPSATRKD-IPSSAALPVMRPAL
CpPLD12         ------------------------------------------------------------
SmaPLD11        VKEWVSNLRLGDQQ-SDINVSLHPQRSSGGEQGGDENNQSSKRGDHIPSSAVFPVMRPAF
SmaPLD12        VKEWVQSLGLGEHSPSGVNLHSH-------DDYDQEDNPPAPRKD-IPSSAALPVMRPAL
SmaPLD13        LKEWMHSLGLGDEQ-LGVNVALQPQVSD--EEGTDEISQFSSKTG-IPARAVLPVMRPAF
FaPLD11         AREWFQNLGIGDH--TVINLQEE-------EEPEQDEAPSITRKD-IPSSAALPVMRPAL
PsPLD10         AREWFQNLGIGDH--AVINLQEE-------EEPEQDDAPSTSRKD-IPSSAALPVMRPAL
PsPLD11         ------------------------------------------------------------


PpPLD13         ---------AQTAMQDYIKHFLDDLDIVNTREVCRFLEVS
PpPLD14         -----------AAMQNYLNHFLESLDIVNTVEVCKFLEVS
SfPLD17         GRLPTISHRAQSAMQHYLKHFLDNLDIVNTREVCRFLEVS
SfPLD18         GRLPTISQRATSAMQNYLNYFLASIELVNTREVCRFLEVS
SfPLD19         GRLPTISQRATSAMQNYLNHFLASLEIVNTREVCRFLEVS
CpPLD11         GRLPTITYRAQNAMQDYLRHFLDDLDIVNSREVCRFLEVS
CpPLD12         ----------TAAMQNYLNHFLESLDIVNTSEVCKFLEVS
SmaPLD11        GRLPTISQRATSAMQNYLNHFLASLEIVNTREVCRFLEVS
SmaPLD12        GRLPTISHRAQSAMQHYLKHFLDNLDIVNTREVCRFLEVS
SmaPLD13        GWLPTISQRATSAMQNYLNYFLASIELVNTREVCCFLEVS
FaPLD11         GRLPTITYRAQNAMQDYLKHFLDDLDIVNSREVCRFLEVS
PsPLD10         GRLPTITSRAQNAMQDYLKHFLDDLDIVNSREVCRFLEVS
PsPLD11         ----------SAAMQNYLNHFLESLDIVNTIEVCKFLEVS
                            ***.*:.:** .:::**: *** *****
```

5 Alignment of PH domain of moss *PLDs:*

```
PpPLD13         -------------------GCLGCFRSCCCC-NPTWQKVWAVLKPGFLAILGDPFDAKPL
PpPLD14         --------------------------SCWCCFNTNWQEVWAVLKPGFLVLLADPFVGKPL
SfPLD17         EGYVLVQHLPKFSTEDSSGGCSNCFRMCHCC-SPNWQQVWAVLKPGFLALLVDPTNPKPL
SfPLD19         -----------------PSCCSSFWMSCRCWLNTNWQQVWLVLKPGFLALLADPLDVKAL
CpPLD11         EGYVMVQHLPNFSKE--STGCLACFKSCCCC-NPNWQRVWAVLKPGFLAILGDPFDAKPL
CpPLD12         --------------------------SCWCCFNTNWQEVWAVLKPGFLVLLADPLDAKPL
SmaPLD11        -----------------------FWMSCRCWLNTNWQQVWLVLKPGFLALLADPLDVKAL
SmaPLD12        EGYVMVQHLPKFSTEDSSGGCSNCFRMCHCC-SPNWQQVWAVLKPGFLALLVDPTNPKPL
FaPLD10         ------------------------------SFNTNWQEVWAVLKPGFLVLLADPLDAKPL
FaPLD11         EGFVMVQHLPNISQ---NSGCLACLKSCCCC-NPNWQRVWAVLKPGFLAILGDPFDAKPL
PsPLD10         EGFVMVQHLPNFSQ---NSGCLACLKSCCCC-NPNWQRVWAVLKPGFLAILGDPFDAKPL
PsPLD11         ------------------------------SFNTNWQEVWAVLKPGFLVLLADPFDAKPL
                                                ...**.** *******.:* **   *.*

PpPLD13         DIIVFDVLP-----PLDRGATEGLIALARLNKERNPLRFSFTVTCGTRTVRIRTQRAGRA
PpPLD14         DIILFDML----------SSSENQVALAEPGKERNPLKFSFVVNCGNRELKFRTSRAVSA
SfPLD17         DIIVFDVLV-----PSDRGATDGLIALARMGKERNPLRFSFLVTCGNRVVKFRTQQARSA
SfPLD19         DIILFDVLPSTMGNPRD-AAGLELKSLAIVTKEKNPLRFSFTVNCGNRAIKMRTAWATGA
CpPLD11         DIIVFDVLP-----PVDRGATEGLIALARLNNERNPLRFSFMVTCGTRSVRIRTQRAGRA
CpPLD12         DIIVFDVL----------GSSEKPITLAELRKERNPLKFSFNVNCGNREVTFRTGRAVSA
SmaPLD11        DIILFDVLPSTMGNPRD-AAGLELKSLAIVTKEKNPLRFSFTVNCGNQAIKMRTAWATGA
SmaPLD12        DIIVFDVLV-----PSDRGATDGLIALARMGKERNPLRFSFLVTCGNRVVKFRTQQARSA
FaPLD10         DIIVFDVL----------GSSEKPNALAKLEKDRNPLKFSFVVTCGNRELTFRTGRAVSA
FaPLD11         DIIVFDVLP-----PLDRGATEGLIALARLNKERNPLRFSFMVTCGTRSVRIRTQRAGRA
PsPLD10         DIIVFDVLP-----PLDRGATEGLIALARLNKERNPLRFSFMVTCGTRSVRIRTQRAGRA
PsPLD11         DIIVFDVL----------GSSEKPTALAKLEKDRNPLKFSFVVNCGNRELTFRTGRAVNA
                ***:**:*          .:     :**   :::***:*** *.**.: : :**  *  *

PpPLD13         KDWVASINDA
PpPLD14         RDWVDAINNA
SfPLD17         RDWVAGINDA
SfPLD19         RDWVTAINDA
CpPLD11         KDWVANINDA
CpPLD12         RDWVDAINDA
SmaPLD11        RDWVTAINDA
SmaPLD12        RDWVAGINDA
FaPLD10         RDWVDAINSA
FaPLD11         KDWVANINDA
PsPLD10         KDWVANINDA
PsPLD11         RDWVDAINNA
                :***  **.*
```

6 Alignments of HKD1 and HKD2 motifs of moss *PLDs:*

	**HKD1**	**HKD2**
CpPLD2	HQKSVTVD	HSKVMIVD
CpPLD3	HQKSVIVD	HSKMMIVD
FaPLD5	HQKSVIVD	HSKMMIVD
PsPLD9	HQKSVIVD	HSKMMIVD
PpPLD6	HQKTVIVD	HSKMMIVD
PpPLD11	HQKTIIVD	HSKMMIVD
PpPLD8	HQKTVIVD	HSKMMIVD
FaPLD7	HQKSVIVD	HSKMMIVD
PsPLD7	HQKSVIVD	--------
PpPLD2	HQKSLIVD	HSKMMIVD
SfPLD5	HQKTVITD	HAKMIIVD
SmaPLD1	HQKTVITD	HAKMMIVD
FaPLD8	HQKTIIVD	--------
PsPLD2	--------	HSKMLIAD
CpPLD6	HQKTIIVD	HAKMLIAD
PpPLD3	HQKTIIVD	HSKFMIVD
CpPLD5	HQKTIIVD	HSKFMIVD
CpPLD9	HQKSVILD	HAKMMIVD
SfPLD4	HQKTIIVD	HAKMMIVD
SmaPLD8	HQKTIMVD	HAKMMIVD
SfPLD9	HQKLLVVD	--------
SmaPLD2	HQKLLVVD	HAKMMIVD
SfPLD6	HQKTIIVD	HAKMMIVD
SmaPLD6	HQKSIIVD	HAKMMIVD
FaPLD4	HQKVVCMD	HSKIMVVD
PsPLD1	--------	HSKIMVVD
PpPLD10	HQKVVCMD	HAKIMVVD
CpPLD10	HQKVVCMD	HAKMMVVD
SfPLD11	HQKVVVMD	HAKMMVVD
SmaPLD10	HQKVVVMD	HAKMMVVD
FaPLD6	HQKMTIVD	HSKGMIVD
PsPLD4	HQKMTIVD	--------
CpPLD8	HQKMTIVD	HSKGMIVD
PpPLD1	HQKMTIVD	HSKGMIVD
FaPLD3	HQKVTIVD	HSKGMIVD
PsPLD8	HQKMTIVD	HSKGMIVD
FaPLD2	HQKSVIVD	HSKGMIVD
PsPLD5	HQKSVIVD	--------
CpPLD7	HQKCVIVD	HAKGMVVD
PpPLD5	HQKTVIVD	HAKGMIVD
PpPLD7	HQKITIVD	HAKGIIVD
CpPLD1	HQKITIVD	HAKGMIVD
SfPLD12	HQKVVIVD	HAKGMVVD
SmaPLD5	HQKVVIVD	HAKGMIVD
SfPLD1	HQKVVIVD	HAKGMIVD
SfPLD2	HQKLMIVD	HSKGMIVD
SmaPLD4	HQKLMIVD	HSKGMIVD
FaPLD9	HQKCVIVD	--------
PsPLD6	HQKCTIVD	--------
CpPLD4	HQKITIVD	HAKGMVVD
PpPLD4	HQKTVIVD	HAKGMVVD
PpPLD12	--------	HAKGMIVD
FaPLD1	HQKLTILD	HAKGMVVD
PsPLD3	HQKITILD	--------
SfPLD3	HEKMLIVD	HSKGMIVD
SmaPLD3	HEKMLIVD	HSKGMIVD
SfPLD8	HPKGITSD	HQKVLIVD
SmaPLD7	HPKGITSD	HQKVLIVD
SfPLD7	HQKLIIMD	HAKGMVVD
SmaPLD9	HPKGITSD	HQKLVIMD
FaPLD10	HEKLVIVD	HSKLMIVD
PsPLD11	HEKLVIVD	HSKLMIVD
CpPLD12	HEKLVIVD	HSKLMIVD
PpPLD14	HEKLVIVD	HSKLMIVD
SfPLD18	HEKLVIVD	HSKLMIVD
SmaPLD13	HEKLVIVD	HSKLMIVD
SfPLD19	HEKLVIVD	HSKLMIVD

SmaPLD11	HEKLVIVD	HSKLMIVD
FaPLD11	HEKFVIVD	HSKVMIVD
PsPLD10	HEKFVIVD	HSKVMIVD
CpPLD11	HEKFVIVD	HSKIMIVD
PpPLD13	HEKLVIVD	HSKIMIVD
SfPLD17	HEKLIIVD	HSKIMIVD
SmaPLD12	HEKLIIVD	HSKIMIVD
SfPLD16	HAKYVVSD	--------
SfPLD20	--------	--------
SfPLD15	--------	--------
SfPLD10	HAKYVVSD	--------
SfPLD14	--------	--------
SmaPLD14	HAKYVVSD	--------
SfPLD13	HAKYVVSD	--------
CpPLD13	HAKFVVSD	--------
FaPLD12	--------	--------
PpPLD9	HAKFAVSD	--------
