# Supplementary material for: Characterization and evolutionary diversification of the phospholipase D gene family in mosses
Source: Front Genet. 2022 Oct 13;13:1015393. doi: 10.3389/fgene.2022.1015393 (PMC9607936; doi:10.3389/fgene.2022.1015393)
Supplement: Supplementary file 11 [file DataSheet1.DOCX]

**Supplementary data sheet 1. The sequence alignment used for phylogenetic tree reconstruction.**

>AfPLD1

YVCMSLVVASTNVISPCWEQHIGKIEASCLSS--DGKATLEISLEYLPVEHYKGLADVPD

GCLPEIDLDEFEHSRCWADISKAIIDAKHLVYIAGWSIYTPVKLVRNSDTLGNLLKEKAE

DGVQVLLFVWDDYTSFQNDGVMNTYDELLARRHGHVVETFYSHHKKIVLVDVAFLGGLDL

CKGRYDTPHHSLFSNLDTTFDGDFYNTFESEGPRQPWHDLHCKIEGPAAYDVLKSFEQCW

QKTLFHLNKDKNWHIQ-------------DMGIHSAYIKAIRSAQHFIYIENQYFIGSSK

TAGSNHLVPIEIALKIAKKIKSRQPFAVYIVIPMWPEGIPNNNVIQKMLFFQSQTMEMMY

TIINALKEMNSTDFLNFFCREPMIYVHSKGMIIDDEYLVMGSANINLWAEHLGFQVHRVQ

ELANENWKKYPYPINT

>AfPLD10

YLNHFLLIVNTREVCPKLREGLKADPVFDALPSDKERRTIRIRSKRAGIARWVCHSHRYG

SFAPPRGDYDIDGRAGFHAIASAIEHAKSEVFIAGWWLCPELYMRRPFTRLDTLLQERAQ

MGVKIYILLYKEVALLKKRRLLTIHENVKVLRFPNAGVYLWSHHEKIVIVDHCFIGGLDL

CFGRYDTQEHTVHDTSSRIWGKDYYNRERQQYPRMPWHDVQCAIWGPACRDVARHFVQRW

RSKAMVLPEQEWWQDQVLRSVGFWSATSQETSIHIAYCSLIDKSEYFVYIENQFFIGFDD

DDAIRNRVLQALYNRIIQAHKEQKCFRVIVVIPLLPVGVDDAGAVRAIMHWQYRAISRGK

YSLQRLNDAAAENYVSFYGYGPQVYVHSKIMIIDDRVVLLGSANVNLWREHLGLQQSEMN

LLIDHTWRAYLFPLES

>AfPLD2

YATIDFAVARTRLVKPMWNESIGQIPTNLVKSGNR--GKIYVKLQFLDARQWGNKAHMQD

GFMPRIPLAGYQPTRCWEDIFKALSDAKHLIYITGWSIYSQITLVRDMETLGQLLKRKAS

EGVRVLMLVWDDRTSIDTTGVMHTHDEVLCPRNPDEIGLIFTHHQKTVIVDESFVGGIDL

CDGRYDNQKHSLFSTLASTHKEDFHQCFKKGGPREPWHDVHSKIEGPAAWNVLYNFEQRW

EKQGRLLKIPEAWNVQIFRSIDEGAVANADRSIQDSYICAVRRAKNFIYMENQYFIGSSR

DSGAIQLIPMEITQKIVSKIEAGERFSVYIVIPMWPEGVPESEAVQNILYWQRLTMEMMY

KRINALEKKSPTDYLNFFCREPMIYVHAKTMIVDDEYIIVGSANCNLWYEHMGFLVRKVQ

RISKDLWGWYYYPILS

>AfPLD3

YATVDLAVGRTHMVSPVWRESVGRIPVQSLLSGANNNGKVRVSLQFLCVQKWMCHAEMQP

GFLPSIYLSDYKGSRCWEDISDAISDAKIFIYIAGWSIYTEISLRRDGSTLGELLKRKAE

EGVSVAILVWDDRSSIRPQGVMDTHDELLCPRIPDQIATMFSHHQKLVSLDVSFMGGIDL

CNGRFDDQRHSLFRTLNAEHSRDFYQCFASGGPRQPWHDIHCQLQGPIAWDVVSNFEQRW

KKQGVLADNPEAWNAQLFRSMDAGAAAAEERSIQDAYIQAIRSADRFIYIENQYFIGSCQ

NVGCLNLIPMEITLKIVSKIEAGERFVAYIVIPMWPEGVPDSVTVQAILDWQRRTMEMMY

KKIFALRRSSPSDYLCFFCREQMIYVHSKMMIVDDEYIIVGSANINLWYEHLGFLVRYVC

KVSNHLWDLFTYPVE-

>AfPLD4

YVCFILALARTDVISPVWEQSIGKIPSERVLSSYDDKAELQISLTYWPVDRYRCRAHVPH

GLQPQIQLAGFEPRSCWDDICQAILDAQHLIYIAGWSIYTKITLVRDKENLGELLKWKAA

CGVRVLLLVWDDKTSFQTSGLMGTHDELLAPRHIDVDGSFFTHHQKLVVVDTAFIGGLDL

CNGRYDTPEHSLFCKLDTVFKDDYYNIFNSKGPRQPWHDLHCKIEGPAVYDVIKNFEERW

RRQTALVDASERWYIQVFRSIDSGSV---DHPLRGQDIKMLVPPKLFWYIQQH---ATDQ

PCGSNHLVPMELALKIVSKIKAHEEFAVYVIIPMWPEGDPPTQTVQEILFFQSQTMEMMY

TLIEALKEEHPTDYLNFFCREPMIYVHSKGMIVDDEYVIIGSANINLWAEHLGFLVNMVQ

HLAAVNWKQYPYPLST

>AfPLD5

YVSLSFALARTRVVKPTWQETL-HVDATELLTGRN--GRISLNLKFIPVHEWCNLAHVPD

NFCPPISLRGYRPARCWEDLYTAISGAKHLIYIAGWSVFTSITLVRDGATLGELLKRKSE

EGVRVLMLVWDDTTSLKTNGVMHTHDEVLCPRNPDKTELMFTHHQKTVVTDVAFVGGIDL

CDGRYDTPHHSLFRTLGPVHANDFHQNFGMGGPREPWHDVHARVEGPCAWDILKNFEDRW

KRQGSLVKVQEAWDGQVFRSIDAGAVAAGDRSIQDAYICAIRRAKHFIYMENQYFVGGAQ

NSGAQQLIPMELTLKIVRKIEAGERFTVYVVIPMWPEGVPESSSVQSILHWQHCTMEMMY

KRIKAIEDNKPTDFLNFFCREPMIYVHAKTMIVDDEYIIVGSANCNLWYEHLGFKVNKVQ

FLSQSFWNLYPYPIHT

>AfPLD6

YVSIHMAVAKTRIIKPVWNESVGKVPVSDILSGQNDNARIHIRMQYFDISTYGCKSHVPD

SFDPKIYLSGHEFHRCWEDIYKAISGAKHLIYVTGWSIYTKIELVRDPDTLGQLLKNKAS

EGVRVNMLVWDDRTSIKKNGIMGTHDEVLCGRNPDEISTMFTHHQKTVVVDASFVGGIDL

CDGRYDTQRHSLFRTLGPPHHEDFHQNFTKGGPREPWHDIHCRIEGQAAWDILHNFEQRW

EKQGKLVSNPSSWHAQVFRSIDAGAAAARDRSIQDAYIHAIRRAKSFIYIENQYFLGSSN

DAECVHLIPIELALKIADKIKARERFAAYVVMPMWPEGVPTDGSVQAILFWQRRTIEMMY

RIIDALRDANPKDYLSFFCREPMIYVHAKMMIVDDEYILVGSANINLWCEHLGFLIRRVN

AISDELWRLFTYPIKS

>AfPLD7

YACVILAVARTQVISPQWNEHMGQIQAESILSKGDNKVQIEISIKYISVADYKGRAHVPD

GCLPSFELQNFEHGKCWEDIFNAIVDARHLVYIAGWSIYTKIKLLRGERTLGELLKSKAQ

ETARVLLLVWDDKTSIGNVGVMGTHDELLAPRYADVIGSLYTHHQKVVIVDAAFIGGLDL

CDGRYDTPNHTLFKNLNTTFADDYHNTFESGGPRQPWHDLHCKIEGPAAYDILTNFEQRW

RKAA----VPESWHV--------------DMSIHTAYISAIRSAQHFVYIENQYFLGSSQ

EAGANHLVPIELALKIVSKIKANERFSVYVVIPMWPEGIPTSSSVQEILHFQTQTMEMMY

LLIKALEEMHPTDYLSFFCREVMIYVHAKGMIVDDEYVILGSANIN--FEQGAYEGRQLE

AVCTRG----LYPIQT

>AfPLD8

YASVEMAVGRTRIIEPVWNESMGKIPVTNVLSGDNDEARIHVKMQYFDVSTFGCKAHIPD

NFNPQIYLSNYEPQRCWEDIYKAICDAKHLIYITGWSVYAEVELIRDKDTLGQLLKRKAS

EGVRVNMLVWDDRTSWKKDGLMATHDQILCPRNPDQIGTMFTHHQKIVVVDASFVGGIDL

CDGRYDTPFHPLFRTLDTVHKSDFHQNFEKGGPREPWHDIHTKVEGQAAWDVLHNFEQRW

EKQGKLLTISSTWSVQIFRSIDAGAAAASDRSIQDAYIHAIRRAKNFIYIENQYFLGSCK

NCGASHLIPVELALKVASKIEAGERFSVYVVVPMWPEGVPESGSVQAILDWMHKTMEMMY

KIIQALQAKSPRDYLTFFCREPMIYVHSKMMIVDDEYIIAGSANINLWYEHTGFLVRAVN

TIGDELWKKFTYPVKC

>AfPLD9

YLNHFLLIVNTREVCPKLAEGLKADPVFDVLPAAKERRTIKLRTKRSGNARWVCHPHRYG

SFAPPRGDDEIDGKSAFHAIASALERAKSEVLIAGWWLCPELYLRRPFARLDTLLRKRAE

MGVKIYILLYKEVALLKKRLLLAVHENIKVLRFPNAGVYLWSHHEKIVIVDHCFLGGLDL

CFGRYDSQDHRVHDSSSRIWGKDYYNRERQQYPRMPWHDVHCAIWGPACRDVARHFVQRW

RSKAMVLPKQEWWQGQVLRSVGFWSATSQETSIHTAYCSLIDKAEYFIYIENQFFIGFDD

DDVIQNRVLQALYDRIMRAHKEGKCFRVIIIIPLLPGGVDDAGAVRAIMHWQYRTISRGR

HSLQRLDNAETENYVSFYGYGPQVYVHSKIMIIDDRVVLMGSPNLNLWREHLGLQHSEAS

VLMDHMWMAYLFPLES

>AfPLD13

YATVDLAVSRTHMVEPVWEESVGRIPVQSLLSGDNGSGTVKVSLQFFKAQSWTCHADMQD

GFLPKIHLSGFQPSCCWKDIYEAISNAKLFIYIAGWSVYTKISLLRYGDTLGDLLKRKAE

EGVCVALLVWDDRTSVKQQGIMGTHDELLCPRIADQISTMFTHHQKLISVDVSFIGGIDV

CDGRFDDQRHSLFRTLNAEHSRDFYQCLASGGPRQPWHDIHCQLEGSIAWDVVFNFEQRW

NKQGVLVEAEESWNAQLFRSIDAGAAVADERSIQDAYIQSIRRANHFIYM----------

-----------------------------------------------------RT-----

-------------------------------------------NIS--------------

----------------

>CpPLD1

YTTVVLAVARTRVISPVWKEHMGSISAGSVLNGGQGNAQLQFSARYMPVEQYTCRAHIYD

NTLPSIPLDGFVQARCWEEMCTAINDAKVLVYIAGWSVFDKITLVRDLNTLGELLKKKAS

QGVRVLLLVWDDKTSVKTAGVMNTHDEVLAPRYGAVVGTLYSHHQKITIVDTSFIGGLDL

TGGRWDTPSHTLFASLQKEHKNDFRNSWESGGPREPWHDWHCRIEGHAAYDVLKNFEQRW

MKAAELIDRDETWHVQVFRSIDSGSVVQKDISIQMAYIKAIRSAQHFVYIENQYFLGSST

TAGANHLIPMELTLKICSKIREGKRFAVYVVIPMWPEGIADSAPVQEILYFQSQTMKMMY

AKIDTLRETKPTDYLNFYCREPMIYVHAKGMIVDDELVIMGSANINLWAEHLAFEVQRIN

YMADRNWEQYRYPLNQ

>CpPLD10

IATVELVVARTRPIKMKFQESSGKISTEQVLDGEDREAKFHIVLQFTQAMKWDCKSHIDE

AFNPKIMLDNYKPEKGWETLYHAMNDAKHFIYVAGWSVNATIALVRDKETIGELLVRKAN

EGVTVLMLVWDDKTD--LTGIMNSHDELLCPRNPEVAGFIFTHHQKVVCMDAAFQGGIDL

CDGRYCFPSHPLFQYLDTLFKNDYHQSIAHGGPREPWHDCYSKLEGEIAWDVHENFRQRW

LKQAQLVSNRESWNVQLFRSLDETSGIFNERSIQDAYIQAIRRAKRFLYIENQYFLGSSR

DESAAQLIPLEIALKIASKIRAGEDFSVYIVNPLWPDGPPASLSGQDIMLFHRKTLEMMY

RIIKALIDMDMTHYLSFYCREPMIYCHAKMMVVDDEYLIIGSANINTWFEHLGFDKKRVN

ELATRYWEEYRYPYEK

>CpPLD11

YLRHFLLIVNSREVCPKLHEGLKGDPVFDVLPVDNERRSVRIRTQRAGRAKWVCHPHRFG

SFAPPRGF-SVDGKAAFEAIAAAIENARSEIFIAGWWLCPDLYLRRPFSRLDHLLESKAK

MGVQVYILLYKEVALLKKQRLLGLHENIKVLRFPDSGVYLWSHHEKFVIVDHCFLGGLDL

CFGRYDSPDHRVSDYPSAIWGKDYYNRERQKVPRMPWHDVHCALWGPPCRDVARHFVQRW

RSKAMVIPQSEWWDVQVIRSVGQWSATSQERSIHEAYCSLIERAEYFVYIENQFFIGLEG

DDTIHNRVLQALYSRIMRAYKEHRCFRVIVVLPLLPGGVDDGGAVRAIMHWQYRTICRGK

NSLERLSNEQAEDYITFYGHGPQIYVHSKIMIVDDRSVLIGSANINLWAEHLGLRASEVN

AIRDDLWMSYFFPLES

>CpPLD12

YLNHFLLIVNTSEVCPKLQEGLKADPVFDVLG--KERREVTFRTGRAVSARWVCNPHRFG

SYAPQRGLASVDGKAAFEAIAVAIESAQSEIFLTGWWLCPELYLRRPFMRLDVLLESKAK

AGVQIYVLLYKEVSMLKKRRLQGIHENIKVLRWPDSGVYLWSHHEKLVIVDHCFLGGLDL

CYGRYDDPCHRVWDKPPSIWGKDYYNRERTKLPRMPWHDVQCAIWGPACRDVARHFVQRW

RNKAMVIPVTDWWGVQVIRSVGQWSATTQERSIHAAYCSLIERAEHFVYIENQFFIGMDG

DETIQNRVLQALYARIMRAHREGQCFRVIVVMPLLPGGVDDSGAVRFILHWQFRTICRGR

HSLQRLKDSKANDYVSFHGFGPQVYVHSKLMIVDDRFVLIGSANINLWSEHLGIDAADVE

TILDKVWVSYNFPLES

>CpPLD13

VVVVVLVVLVS--IGLMQDEDVQSIPVEGVLNGDRGNKGLDVTAQY-----WE-------

---PRSPRSG--------EYGFSEQEMERFGASAGKAVY-------------ESLLAAAD

RGVP-----------LQHSGFSPSFDA----------------HMKLWMANKAYLGSA--

---NND------------------------------WKSLTQVKEGVYFKLLEGYFNNFW

--NAVVWD--QQWQLLFPKYTITPHVLSTYQIDEQGWLDTILSVPMNVRINTQFL-DAD-

---------------FTDAISKVKGATVKVLVAHWRT-------TEEYL-----------

---------MYLNYTNSLCKSPTRVNHAKFV-VSDVRANIGTSNL-VWDYYTGFGVSQLQ

AIFDADWISYAIPLSL

>CpPLD2

YATVDLTVGRARVLEPVWNETVGKVPVIDLLSGRHTGAHIRFSLQFFDAARWGCRAHMAD

NFLPNIYLANYQPTRCWEDVFEAIYNAQKLIYITGWSVYTEIKLVRDPRTLGELLKRKAD

QGVRVNVMVWANLT--RALGVINTHDQFLSPRYPDQIAGLFTHHQKSVTVDASFVGGLDL

CDGRYDTQYHSLFRTLDTTHATD---LFECGRPRQPWHDIHSRLEGPVAWDVLENYEQRW

RKIASLLPIGETWTVQLFRSIDAGGSAAEDRSIQDAYINAIRRAKNSIYIENQYFLGSSQ

EAGAYNLIPMELVRKIVTKIEAGERFTVYIVVPLYPEG--SIVEVQAILDWQRRTFQMMY

KEIDALSKKHPREYLAVYCREPMIYVHSKVMIVDDEYIILGSANINLWCEHAGFLIRKLN

ERADELWGLWAYPYRF

>CpPLD3

YATVDLTVGRTRVLEPVWNETVGKVPVIDLLSGRHTGAHIRFSLQFFDAARWGCRAHMAD

NFLPNIYLANYQPTRCWEDVFEAIYNAQKLIYITGWSVYTEIKLVRDPRTLGELLKRKAD

QGVRVNVMVWDDRSSLRQVGVMTTHDEFLCPRNADQIGGLFTHHQKSVIVDASFVGGLDL

CDGRYDTQYHSLFRTLDTTHATDFHQVFECGGPREPWHDIHSRLEGPVAWDVLYNFEQRW

RKQAALLPIGETWNVQLFRSIDAGAAAAEDRSIQHAYINAIRRARNFIYIENQYFLGSSQ

EAGAYNLIPMELVRKIVTKIEAGERFTVYIVVPLYPEGIPESQSVQAILDWQRRTFQMMY

KEIIALNSKHPREYLTVFFREPMIYVHSKMMIVDDEYIIVGSANINLWFEHAGFLIRRLN

QRADELWELWAYPYTS

>CpPLD4

YAAVNLAVARTRVISPKWDEYFGTIPVDKVIHGESGKAQILFKIKFTPVERYFCEAHIMD

GALPEIKLEGFQHRKAWEELCTAILEAHHLVYIAGWSIYTKVRFWRDTTTLGELLKRKSA

EGVRVLLLVWDDKTSIKTEGVMGVHDEVLAPRYADVVGTLYTHHQKITIVDSSFIGGLDL

CDGRFDTPSHCIFSTLNTFHKNDFHQTFDAGGPREPWHDWHCKIDGPAAYDVLTNFEQRW

RKATELIQISNTWYTQVFRSIDSGSVAEKDISIQMAYIKAIRSAQHFIYIENQYFIGSSK

DAGANHLIPMELALKIASKIREGQHFAVYVTIPMWPEGVPDSAAMQEILFFQTQTMKMMY

GVIDALKDVHPRDYLNFYCREPMIYVHAKGMVVDDEYIICGSANINLWAEHLGFDVQEVN

RLAQANWDQYPYPINQ

>CpPLD5

YAVVDLTVGRTRVLCPQWNESIGKVPTESLLSGEGDESQVRFRLQFFDATHWGCKAHMAE

DFLPAIHQAGRQPTRLWEDVFEAINNAQHLIYITGWSVYCEILLVRDPRTLGELLKKKAS

EGVRVNMLVWDDKTSLKRDGLMATHDEHLCPRNPDTIGTMFTHHQKTIIVDASFVGGIDL

CDGRFDNQNHSLFRTLNDLNMNDFHQNYAKGGPREPWHDIHSKVEGPVAWDVLWNFEQRW

QKQAKLIPIEDTWNVQLFRSIDAGAAIAKDRSIQDAYINAIRRAKDFIYIENQYFLGSSQ

DAGAIHLIPMELTRKIVSKIEDGERFTVYVVVPMWPEGIPESGSVQAILDWQKKTMEMMY

TEIAALRAQSPRDYLTFFCREPMIYVHSKFMIVDDEYTIIGSANINLWYEHIGFLIRKVN

EIGDQYWSMFSYPIKS

>CpPLD6

YATVDLSVGRTRVLKPLWNESIGKIPAETLLSGSGDEGEIRFRMQFFEASHWGCKAHMED

GFLPTIYQAGRQPTHCWEDVFKAIDGARHLIYITGWSVYCEILLVRDPRTLGELLKKKAA

EGVRVNLLVWDDKTSLKRDGLMATHDEHLCPRNPDQIGNMFTHHQKTIIVDASFVGGIDL

CDGRYDNQNHSLFRTLNDIHMNDFHQNFTKGGPREPWHDIHARVEGPVAWDVLWNFEQRW

QKQALLIPIDDTWNVQLFRSIDAGAAVARDRSIQDSYINAIRRAKNFIYIENQYFLGSCQ

DAGALHLIPMELTRKIVSKIEDGERFTVYVVVPMWPEGIPESGSVQAILDWQHKTMEMMY

TEITALRAATPRDYLTFFCREAMIYVHAKMLIADDEYIIIGSANINLWYEHIGFLIRKVN

EAADRHWAMWTYPVKS

>CpPLD7

YTVVVVAVARTRVISPKWNEHMGKISAGPLLNGGEGKAKLRVSARYIPVEQYTCRAHVYD

HTLPNIMLDGYAHGRCWEDICSAINDAQHLIYITGWSVWDKVALVRDANTLGELLKKKAS

QGVRVLLLLWDDKSSVKTSGVMNTFDEVLAPRYGAVVGTLYTHHQKCVIVDSSFVGGLDL

TAGRWDTPSHYIFASLQREHKDDFRNSWGSGGPREPWHDLHCKIEGHAAYDVLTNFEQRW

RKATELIDHEETWHVQIFRSIDSGSVVHKDISIQMAYINAIRCAQHFIYIENQYFLGSST

SAGANHIIPMELALKICSKIREGKRFAVYIVVPMWPEGVPDSAPVQEILYFQSQTMKMMY

GKVDALREVKPTDYLNFYCKEPMIYVHAKGMVVDDELVIMGSANINLWAEHLGFEVQRVN

YTADRNWEQYRFPLNQ

>CpPLD8

YVTVVVAVARTRVISPEWREHLGKISVEKVLNGGQGRAQLCLSASYIPVEQYTCRTHIYD

HTLPNIRLDGYEPRRCWEDLCAAIYDAQHLIYIAGWSVYDKVRLIRDYNTLGELLKLKAS

QGVRVRLMIWDDKTSVKTEGVMNTHDEVLASRYGAVVGTLYSHHQKMTIVDSSFIGGLDL

TAGRWDTPSHKLFSSLDHEHKGDFRNSWDSGGPREPWHDWHCKIEGHAAYDVLTNFEQRW

NKVAQLLNREDTWQVQIFRSIDSGSVVTKDISIHMAYVKAIRSAQHFIYIENQYFLGSSK

TAGANHLIPMELTLKICSKIREGKRFAVYIVIPMWPEGIPDSGPVQEILFFQTQTMKMMY

SLVDTLRDCKPIDYLNFYCREPMIYVHSKGMIVDDEYVISGSANINLWAEHLGFEVRRVN

DMAEKNWQQYRYPLNQ

>CpPLD9

YATVDLAVGRTGIVKPVWNESLGDVPAQQLLNGEDNESEIRFRLQFFQASHWGCKAHMED

GFLPPIYLSGRQPTRCWEDIFEAISNAKHFIYITGWSVYTEITLVRDPSTLGELLKKKAT

EGVRVNMLVWDDRTAVQPAGLMGTHCLYLCPRNPDTISTMFTHHQKSVILDASFVGGIDL

CDGRYDNQRHSLFHTLDNLHAHDFHQNFEKGGPREPWHDIHSKLEGPIAWDVLYNFEQRW

RQQAKLLPIGETWHVQFFRSIDAGAAAERDRSIQDAYINAIRRAKDFIYIENQYFLGSSQ

DAGADHLIPAELTRKITSKIADGERFTVYIVMPMWPEGIPDSGSVQAILKWTMNTMEMMY

TEITALRASTPQDYLTFFCREPMIYVHAKMMIVDDEYIILGSANINLWYEHTGYLIRMIK

AIGDQHWAMWTYPVKS

>CrPLD1

YVTIDIAVARTRPLMPKWDEKIGQLSTVDLLRNRP--GKLYVKFEFRNVKSWGNKSHMKD

GFMPEIILDRYKPTRCWEDIYTALSEARHFIYIAGWSVFTHITLVRDPKTLGDLLKRKAE

EGVRVLLLVWDDRTS---EGIMHTHDEVLCPRNPDEVGLLFTHHQKTVTVDVCFLGGIDL

CDGRYDEQHHYIFKTLNTVHAKDFHQNFKFGGPREPWHDVHARIEGPAAWDVLKNFEQRW

KKQGHLLSIPEAWNAQIFRSIDEGAVAAQDRSIQDAYICAIRRAKHFIYIENQFFIGSSQ

DSGAIQLIPMELTQKIVSKIEEGERFAVYVVIPMWPEGIPESSPVQDILYWQKLTMEMMY

KRIAALIKKVPTDYLNFFCRE-MIYVHAKTMIVDDEYIIVGSANCNLWYEHTRFEVKKMQ

AISQELWSKYPYPIRS

>CrPLD10

YLNHFLLIVNTREVCPKMREGLKADPVFDVLSSDKERRTLKIRCKRPGGARWVCHPHRHG

SFAPPRGISDIDGKAAFNAISSAIDNAKSEIFIAGWWLCPELYLRRPFARLDVLLQRKAQ

MGVKIYILLYKELALLKKRRLLMIHENVKVLRYPNSGVYLWSHHEKIIIVDHCFIGGLDL

CFGRYDDWKHKVHDSNADVWGKDYYNRERRLYPRMPWHDVHCAIWGPACRDVARHFVQRW

RSKAMVLPEHDWCHEQVLRSVGLWSATSQETSIHLAYCSLIGRAEHFVYIE---------

------------------------------------------------------------

------------------------------------------------------------

----------------

>CrPLD11

YVTLNFAIARTCTVKPIWNESLGHIPPQVLLSGED--GKIKVRLRFIRVEDWGNKAHMED

GFMPPIHLSDHQPTRCWEDIYKAIVNAKHIIYIAGWSVYTKITLVRDMETLGELLKKKST

EGVRVLLLVWDDRTSMRTPGVMQTHDEVLCPRNPDQTRLMFTHHQKTLVVDVAFLGGIDL

CDGRYDTQSHSLFRTLGTMHKDDFHQCIKGVGPREPWHDVHARIEGPCAWDVLYNFEQRW

GRQAALRNVPEAWNGQIFRSIDEGCVAASERSIQDAYISAIRRARNFVYIENQYFIGGGN

NEGAFQLVPTELTMKIVRKIEQGERFSVFIVVPM--------------------------

------------------------------------------------------------

----------------

>CrPLD2

YVCVVLAVARTTVISPEWNEHLGDISAESLLYGSDGKTALKISVEYCPFDQYKGRAHVSP

TLLPSIRLAGFQHRSCWDDICQAIVDAQHLIYIAGWSIYTEITLVRDRNTLGELLKWKAK

GGVRVLLLVWDDMTSINKVGVMKTHDELLAPRYADVVGTIYTHHQKTVVTDAAFLGGLDL

CDGRYDSPEHTLFCKLDTDYKDDYHNTFKSGGPRQPWHDLHCKIEGPAVYDVLTNFEQRW

RRVAFLLDKSEGWHVQVFRSIDSGSVAEKDTSIQKAYIKAIRSAQHFIYIENQYFLGSSK

TPVSSHLVPIEIAQKIVSKIKAHEPFAVYIVIPMWPEGNPTDAAVQEILYFQSQTMEMMY

ALIEALKEEHPTDFLNFFCREPMIYVHSKGMIVDDEYVIIGSANINLWAEHLRFMVKTVK

RLAQQNWDQFQYPLPT

>CrPLD3

YVCVVLAVARTRVVRPKWDEHFGKIRAESLYS--DGKVKLQVSMKYCPVEKYKGRAHVPD

GMLPEIELEGFEHRKCWEEICEAIQGAHHLVYIAGWSIYTKTKLIRKELTLGELLKNKAY

ETARVLLLVWDDKSSINNVGVMGTHDELLAPRYADVVGTLYTHHQKLVIVDAAYIGGLDL

CDGRFDTPDHTLFKSLDTVFADDFHNTFGS-GPRQPWHDLHCKIEGAAAYDVYKNFEQRW

WKAAVFVDVPETWHVQVFRSIDSGSVIEKDMSIHTAYIKAIRSAQHFIYIENQYFIGSSK

NAGANNLIPIEIALKIVSKIKANEDFRVYIVIPMWPEGVPTSTAMQEILFFQSQTMEMMY

THINALREMQPTDYLNFFCREAMIYVHAKGIIIDDEYIILGSANINLWTEHLRFKVKRVR

DLGIENWNQYCYPLST

>CrPLD4

YASVEMAVGRTRVIVPLWNESVGKIPVGDILSGEHDDAKVHLRMQYFDISNLGCKAHVPN

DFNPKIYLGDYQQQRCWEDIYKAIVGAKHLIYITGWSVYTQIELIRDSETLGDLLKKKAE

EGVRVNLLVWNDRTSFRKDGLMATHDEKLCPRNPDQIGTMFTHHQKLVVVDASFVGGIDL

CDGRYDTQFHSLFRTLDTVHKADFHQNFKMGGPREPWHDIHSKLEGQAAWDVLHNFEQRW

LKQGKLVTIPNSWNVQVFRSIDGGAAACKDRSIQDAYIHAIRRAKNFIYIENQYFLGSCK

DAAAVHLIPVELALKIACKIEAGERFSVYVVVPMWPEGIPESGSVQAILDWMHKTMEMMY

KIIQALQAKSPRDYLTFFCREPMIYVHSKMMIVDDEYIIVGSANINLWYEHLGFLIRRVN

SIADDLWDQYTYPVKG

>CrPLD5

YVSIEMAVGRTRVIVPLWNECVGKIPVADILSGEHDKGKIHLRMQYFDITNMNCKAHVPN

NFQPKIYLHDYEQQRCWEDIFKAISGAKHLIYITGWSVYTKIELVRDTEILGDLLKRKAD

EGVRVNLLVWDDRTSLKKDGLMATHDEILCPRNPDQIGMMFTHHQKIVVVDASFIGGIDL

CDGRYDTPYHSLFRTLDTVHKADFHQNFKKGGPREPWHDIHSKLEGQAAWDVLHNFEQRW

QKQGKLLTVPNSWNVQVFRSIDGGAAAAKDRSIQDAYIHAIRRAKNFIYIENQYFLGSCQ

DVGAEHLIPVELALKIACKIEEGEPFRVYIVTPMWPEGVPESDSVQAILNWMHKTMEMMY

KIIQALQAKSPRDYLTFFCREPMIYVHSKMMIVDDEYIILGSANINLWYEHLGFLIRKVN

AIADDLWDQFAFPVKT

>CrPLD6

YVSIEMAVGRTRMIVPVWNESVGKIPVTDLLPGEDGVAKIRLRMQFFDVTTYGCRAHVPN

NFCPKIYLGNYEQHRCWEDIYESICNARHLIYIAGWSVYTEIELIRDKETLGELLKRKAR

EGVRVNLLVWDDRTSFKRDGLMATHDEVLCPRNPDQIGAMFTHHQKIVVVDASFIGGLDL

CDGRYDTQFHSLFRTLDTVHKTDFHQNFKCGGPREPWHDIHCKIEGQAAWDVLQNFEQRW

QKQGRLLIIPHSWNVQVFRSIDGGAAAAKDRSIQDAYIHAIRRAKNFIYIENQYFLGSCQ

SAGCLHLIPVEIALKIASKICAGEPFRVYIVVPMWPEGVPESAAVQAILDWQRRTMQMMY

KLIRALKAEHLRDYLTFFCREPMIYVHSKMMIVDDEYIIIGSANINLWYEHLGFLIRRVN

AIGDELWNQFSYPIKG

>CrPLD7

YVTLDFAIARTRIVQPIWNESLGHIPPQVLLSGED--GKIRVRLRFIRAEDWGNKAHMQD

GFMPPIYLSDHQPTRCWEDIYNAILKAKHIIYIAGWSVYTKITLVRDMKTLGELLKKKSM

EGVRVLLLVWDDRTSFKTPGVMHTHDEVLCPRNPDQTKLMFTHHQKTLVVDVAFLGGIDL

CDGRYDTQSHSLFRTLETMHKDDFHQCIKGVGPREPWHDVHARIEGPCAWDVLYNFEQRW

GRQAALRNIPEAWNGQIFRSIDEGCVAASERSIQDAYISAIRRARNFVYLENQYFIGGGN

NEGAFQLVPIELTMKIVRKIEQGERFSVFIVIPMWPEGAPNSGSVQEILRWQKLTMEMMY

RRIRALRECKPTDYLNFFCREPMIYVHAKTMIVDDEYIIIGSANCNLWYEHLGYLVSRIR

DRSQNLWDIYLYPMHA

>CrPLD8

YVTLDFAIARTRIVKPIWNESLGHIPPEVLLSGED--GKIKVSLRFIKVEDWGNKAHMQD

GFMPPIHLSNYQPARCWEDIYKAIVQAKHIIYIAGWSVYTKITLVRDMKTLGELLKKKSM

EGVRVLLLVWDDRTSMKTPGIMHTHDEVLCPRNPDQTKLMFTHHQKTLVVDVAFLGGIDL

CDGRYDTQSHSLFRTLGTMHKDDFHQSIKDIGPREPWHDVHARIEGPCAWDVLYNFEQRW

RRQAALKNIPEAWNGQIFRSIDEGCVAAYERSIQDAYISAIRRARNFVYMENQYFIGGGN

NAGAIQLVPTELMMKIVRKIEQGERFSAFIVVPMWPEGAPNSGSVQEILRWQKLTMEMMY

RRIRAIKDCKPTDYLNFFCREPMIYVHAKTMIVDDEYIIVGSANCNLWYEHLGYLVSRVR

ARSQHLWDMYLYPMHT

>CrPLD9

YATVDLAVGRTAMIEPEWMESVGKIPIQEVASGANGQGKVRVRLQFFSVQQWACHAHMQH

GFLPRIYLSDFKPARCWEDISDAISAARIFIYIAGWSVFTDISLRRHGATVGELLKRKAE

EGVTVSVLVWDDRTSIKPQGIMGTHDEVLCPRSPDQLSTMFTHHQKLVCVDASFIGGLDL

CDGRYDDQRHSLFRTLDGEHANDFYQSLANGGPRQPWHDIHCRLEGPIAWDVVYNFEQRW

MKQAAPMDHPDSWNAQLFRSIDGGAAAAMEHSIQDAYINAIRQAKNFIYIENQYFMGSCQ

DIGCLHLIPLEITLKIVNKIKAGERFVAYIVIPMWPEGLPESDAVQSILDWQRRTMEMMY

KQILALRSVTPTDYLCFFCREPMIYVHSKMMIVDDEYIIVGSANINLWYEHLGFLVRFVY

GVATELWHLYAYPVQ-

>FaPLD1

YAAVNLAVARTRVISPIWDEHMGIVPVDKILDGENGKASIYFKLKFTPVERYVCKAHIMD

GSLPAITLENFQHRRAWEEICQAILDAHHLIYIAGWSINTKVKLWRDTSILGELLKRKSA

EGVRVLMLVWDDKTSIKTEGVMGVHDEVLAPRYADVVGTLYTHHQKLTILDTSFIGGLDL

CEGRWDTPTHSLFNTMPTIHKHDYHNTFEAGGPRQPWHDWHCKIEGPAAYDILTNFEQRW

RKATDLIQISDSWHCQVFRSIDSGSVAEQEISIQMAYVKAIRSAQHFIYIENQYFI----

--GANQLIPMELALKVASKIRAGQRFAVYIVIPLWPEGAPDSAAMQEMLYFQTQTIKMMY

GVIDALRDVHPRDYLNFYCREPMIYVHAKGMVVDDEYLICGSANINLWAEHLSFTVNEVN

RLAQVNWEQYPYPI--

>FaPLD10

YLNHFLLTVNTIEVCPKLQEGLKADPVFDVLG--KDRRELTFRTGRAVSARWVCNPHRFG

SYAPQRGLTPVDGKAAFEAIAIAIESAQSEIFLTGWWLCPELYLRRPFKRLDVLLESKAK

AGVQIYVLLYKEVSMLKKRRLQGIHENIKVLRWPDSGVYLWSHHEKLVIVDHCFLGGLDL

CYGRYDDPCHRVWDDPPSIWGKDYYNRERTKLPRMPWHDVQCAIWGPACRDVARHFVQRW

RNKAMVIPAADWWRVQVIRSVGQWSATTLERSIHAAYCSLIDKAEHFVYIENQFFIGMDG

DDIIQNRVLEAMYSRIMRAHREGQRFRVIVVIPLLPGGVDDSGAVRFIMHWQFRTICRGR

SSLQRLQNSQARDYVSFHGYGPQVYVHSKLMIVDDRFVVIGSANINLWSEHLGIDASSLE

TILDEVWMSYNFPLES

>FaPLD11

YLKHFLLIVNSREVCPKLHEGLKGDPVFDVLPLDKERRSVRIRTQRAGRAKWVCHPHRFG

SFAPPRGF-SVDGKAAFEGIATAIENARSEIFIAGWWLCPDLYLRRPFGRLDHLLEAKAK

MGVQIFILLYKEVALLKKQKLLGLHENIKVLRFPDSGVYLWSHHEKFVIVDHCFLGGLDL

CFGRYDSPEHRVSDHPSTTWGKDYYNRERQKVPRMPWHDVHCALWGPPCRDVARHFVQRW

RSKAMVIPQSEWWDVQVIRSVGQWSATSQERSIHEAYCSLIEKAEYFVYIENQFFIGLEG

DDTITNRVLQALYSRIMRAYKEHRCFRVIVVLPLLPGGVDDGGAVRAIMHWQYRTICRGK

NSLERLSREQAADYISFYGHGPQIYVHSKVMIVDDWSVLIGSANINLWAEHLGLRANEVK

AIRDDLWMAYFFPLES

>FaPLD12

LLLVLLV-----VLG------VQSIPAPGVLGSDRGNEGLDVTAQY-----WE-------

---PGNPLSG--------DYGFSEEQMKHFGATEGKAVY-------------ASVLAAAE

RGVPLHHLVIELQAELQHTGFSPSFDA----------------HCKLWMANRAYLGSA--

---NND------------------------------WKSLTQVKEGVYLKLLKGYFNNFW

--NAKVAT--QPHSAQL---------LDT-------------------------------

----------------------VKHATVKVLVAHWRT-------TEKYL-----------

---------LYLNSTNALCSSPARVNHAKFA-VSNVRAHIGTSNL-VWDYYVGFGVSQLQ

AIFDADWVSYAVPLKI

>FaPLD2

YTVVVIAVARTRVINPKWKEHMGSIAARVLLHGDEGKAKLRLSARYIPVELYTCRAHVYD

HTLPKIVLDGYVHGLCWEETCTAINEARHLVYVAGWSVWDKVKLVRDAKTLGELLKKKAR

QGVRVLVLAWDDKSSVKTAGVMNTHDEVLAPRYGAVVGSLYTHHQKSVIVDSSFIGGLDL

TGGRWDTPSHYLFASLQNEHKGDFRNSWESGGPREPWHDMHCKIEGPAAYDVLTNFEQRW

RKATELLDHEETWNVQVFRSVDSGSVVQNDVSIQMAYINAIRSAQHYIYIENQYFLGSSK

SAGANHLIPMELSLRICSKIREGKRFAVYVIVPMWPEGVPNSGAVQEILYFQSQTMKMMY

AKIETLREVKPTDYLNFYCKEPMVYVHSKGMIVDDEFVIVGSANINLWAEHLGFEVRRVN

YMADRNWEQYRFPLNQ

>FaPLD3

YVTVVLAVARTRVINPKWEEHIGKIPVERVLNGGQGKAQLELRASYVPVEQYMCRTHIYD

HTLPAIRLDGYEPRRCWEDLVEAMWQAKHMIYIAGWSVYDKVRLIRDSRTLGEMLKVKAS

QGVRVLLLLWDDKTSIKTDGVMNTHDEVLAARYGAVVGTLYTHHQKVTIVDSSFIGGLDL

TGGRWDTPSHSLFSSLDKEHKNDFRNSWDSGGPRQPWHDWHCKIEGHAAYDVLTNFEQRW

RKATELLDHAETWQVQIFRSIDSASVVTKDISIQMAYIKAIRCAQHFIYIENQYFLGSSK

TAGANHLIPMELTLKICSKIKANQRFAVYIVIPMWPEGIPDGGPVQEILYFQTQTMKMMY

RLIDTLRDCKPTDYLNFYCREPMIYVHSKGMIVDDEYVISGSANINLWAEHLGFEVRRVN

EMSDRNWEQYRYPLNQ

>FaPLD4

VATVELVVARTRPVKIKFEESSGKIPTEQLLAGEDHEAKFHIVLQFKPAVTWDCKAHIDE

TFNPKIVLDNYTPEKGWESVYHAMNDAKKFIYIAGWSINATISLIRDKETLGELLVRKAN

EGVTVLVLVWDDKSD--ITGVMHSHDELLCPRNPQVSGFIFTHHQKVVCMDAAFQGGIDL

CDGRYCFPSHPLFQYLDTLFKNDFHQSIAHGGPREPWHDCYSKLEGEIAWDVHENFRQRW

MKQALLVDNRDTWNVQLFRSFDETSGIFKEKSIQDAYITAIRRAKRFLYIENQYFLGSSR

DESAAQLIPLEIALKIASKIRAGEEFSVYVVNPLWPDGAPTSLSGQDIMLFHRKTLEMMY

GIVQALQETDMTDYLSFFCREPMIYCHSKIMVVDDEYLIIGSANINTWFEHLGFDMRRVK

ALASRYWNEYRYPYTV

>FaPLD5

YATVDLTVGRTRILQPVWNETVGRVPVIDLLSGNHTKARIRFSLQFFEAGRWGCRAHMAD

NFLPPIYLANYQPHRCWEDIFESIYNAQHLIYITGWSVYTEIKLCRDPHTLGELLKRKAD

QGVRVNVMVWDDRSSLRQVGVMTTHDEYLCPRNADQIGGLFTHHQKSVIVDASFVGGLDL

CDGRYDTQYHSLFRTLNTTHSADFHQVFACGGPREPWHDIHSRLEGPVAWDVLYNFEQRW

RKQAALLPLGETWNVQVFRSIDAGAAAANDRSIQHAYINAIRRARNFIYIENQYFLGSSQ

QSGAYNLIPMELVRKIVSKIEAGERFSVYIVIPIYPEGIPESQSVQAILDWQKKTFQMMY

KEIIALRSKHPREYLTVFCRETMIYVHSKMMIVDDEFIIVGSANINLWFEHLGFLIRTVN

QRADELWGLFAYPYTS

>FaPLD6

---------------------LGHVPVEQVLDGGQGRAQLQLQARYVPVEQYSCRTHLYD

GFRPRIRLEGYEPRRCWEDLCAAIHGAQHLVYIAGWSVYDKVRLIRDFQTLGELLKAKAG

QGVRVLLMIWDDKTSLKTDGVMDTHDEVLAPRYGAVVGTLYTHHQKMTIVDSSFIGGLDL

TSGRWDTPSHTLFASLEQEHKHDFSNSWDSGGPRQPWHDWHCKIEGHAAYDVLTNFEQRW

TKATELLDRDDTWQVQIFRSIDSGSVVIKDMSIHMAYVKAIRSAQHFIYIENQYFLGSSK

TAGANHLIPMELTLKVCSKIRGGQRFAVYVVVPMWPEGVPESGPVQEILFFQTQTMKMMY

SLVDTLRDCHPTDYLNFYCREPMIYVHSKGMIVDDEYVISGSANIN---------ISQFS

EFSYANFE--WFPVSN

>FaPLD7

YATVDLTVGRTRVLEPVWNESMGKKKVFMLMTGQHTGARIRFSLQFFEACHWGCKAHMAD

HFLPNIYLANYQPTRCWEDIFEAIHNARQLIYITGWSVYTEFKLCRDPHTLGELLKRKAD

EGVRVNLLVWDDRSSLHQQGQMRTHDEYLCPRNPTQIGNLFTHHQKSVIVDASFVGGLDL

CDGRYDTQYHSLFRTLNGAHQQDLHSVFECGGPREPWHDVHAKLEGPVAWDVLYNFEQRW

RKQAALLDSGETC---------SGAAAALDRSLQHAYVHAIRRARHFVYIENQYFIGSAQ

ESGAYNLVPMELARKIVSKIEAGERFAAYVVIPLYPEGAPDSAPVQEILAWQRRTFKMMY

REINAFRAKHPREYLSVFCREPMVYVHSKMMIVDDEYIIVGSANINLWFEHTGFLVRTVN

QRADELWALFTYPFSL

>FaPLD8

YAAVDLSVGRTRILKPVWNESIGKLSAETLLSGEGEEGEIRFKLQFYEASHWGCKAHMED

GFLPQIFQAGREPTHCWEDVFSAISNARHLVYITGWSVYCEILLVRDPNTLGELLKKKAA

EGVRVNLLVWDDRTSFHKDGLMATHDEHLCPRNPDQIGNMFTHHQKTIIVDASFVGGIDV

CDGRYDNQNHSLFRTLNDLNMNDFHQNYAKGGPREPWHDIHSRVEGPVAWDVLWNFEQRW

QKQALLIPIDDTWTVQLFRSIDAGAAVARDRSIQDAYINAIRRAKNFIYIENQYFLGSCQ

DAGALHLIPMELTRKICSKIEDGERFTVYVVVPMWPEGIPESGSVQAILDWQHNTMEMMY

TEITAIRAA-PREYLTFFCREPMIYVHSKMLIADDEYTIIGSANINLWYEHIGFLIREVN

AAGDRHWEMWSYPVKS

>FaPLD9

YAAVNLAVVRTRVISPCWDEHFGNIPVEDVIRGQKGKAKIFFKMKFTSVANYSSHAHIMD

GSLPDIELEGFRHKKAWVELCTAILEAHHLVYITGWSVFTKVRLLRDTRILGDLLKRKSA

EGVRVLLLVWDDKTSIKTEGMMGVHDEVLAPRYADVVGTLYTHHQKCVIVDTSFIGGLDL

CDGRFDTPSHSLFSTLNTFHKNDYHNTFDAGGPREPWHDWHCRIDGPAAYDVLTNFEQRW

RKATELIQISESWHVQVFRSIDSGSVAEMDLSIQMAYIKAIRSAKHFVYIENQYFIGSSK

NAGANHLIPMELALKVASKIRAGERFVVYVVVPMWPEGVPDSAAMQEILFFQVTSI----

------------------------------------------------------------

----------------

>MpPLD1

YATVDLTVGRTRMISPVWNETVGCVPAPVVLSGNDGSSRIRFRLKFVAAADWSCRAHMLD

GFMPHIALDDREPSRCWEDVYQMIVGARHLIYITGWSVYTEIRLVRDEQTLGELLKRRAA

EGVTVNMLVWDDRTS--KDGVMATHDEVLCPRNPDSISLAFTHHQKSIVVDASFVGGLDL

CDGRWDTQQHSLFGTLAGAHRDDFHQNFACGGPREPWHDIHSRIEGEAAWDILYNFEQRW

RMQARLVTVAEAWNVQIFRSIDGGAVAAKDRSIQDGYVSAIRRAKHSVYIENQYFIGSAA

DCPAPHLIPMELTRKIVSKIEANERFAVYVVMPMWPEAVPDTATVQEILQWTHLTMQMMY

KEIRALHARSPRDYLNFYCREPLIYVHAKLMIVDDEYVIVGSANINLWYEHLGFVVRTVN

ALADELWSLYPFPVES

>MpPLD2

YATVDLTVGRTRIIEPVWNESIGKVPVSEVLSGRNGDAKVRFRMQYFAAVDWGCRAHMAD

NFLPPIYLDNHEPTRCWEDAYQMIMGAKHLIYITGWSVYTEIKLIRDEDTLGELLKRKAA

EGVKVNLLVWDDRTSLKRDGLMATHDEILCPRNPDQIGTMFTHHQKTLIVDASLVGGIDL

CDGRYDTQHHPIFRTLATVNHDDFHQNFECGGPREPWHDIHAQVEGRVAWDILYNFEQRW

RKQARLVTQNETWNVQLFRSIDNGAVAAKDRSIQDAYISAIRRAKDYIYIENQYFLGSAR

DSGASHLIPMELALKIVSKIEAGERFAVYVVVPMWPEGAPESGSVQAILNWMHRTLQMMY

KLIQALHAASPRDYLNFYCREPMIYVHSKMMIVDDEYIIVGSANINLWFEHMGFLVQTVN

SIADELWAYYPFPVLQ

>MpPLD3

YVSVVLALARTRVIAPVWNEHLGEVPVSAVVEAGAGKAQLRLKIAYLPVHKY-GNAHVEE

HTLPDFKLDNFQHRRCWEDICTAILEAHHLVYIAGWSIYDKIKLMRDINTLGELLKRKSK

EGVRVLLLVWDDKTSIKTEGVMGTHDEILAPRYGDVVGTLYTHHQKIVICDAAFLGGLDL

CDGRYDTPGHPLFRHMDTVYKEDYHQTFDSGGPRQPWHDLHCRIDGPAAYDVLTNFEQRW

KKATELIEISETWHVQIFRSIDSGSAAERDVSIQTAYIKAIRGAQKFIYIENQYFLGSSK

TAGADQMIPMELALKIASKIRANERFVAYIAIPMWPEGAPDSAAVQEILYFQSQTVQMMY

TIIDAIRDQDPRDYLNFYCREPMIYIHSKGIIVDDEYIIIGSANINLWAEHLGFDVHIIN

EAAEKNWEQYPYPYNT

>MpPLD4

YAAVDLSVGRTRIIEPVWNESIGKVPVTEVLSQRSGDARVRFRMRFYAAVDWSCRAHMPN

GFLEKIYLADKQPTHCWEDAYRMIMGARHMIYVAGWSVYTEITLIRDEDTLGQLLKRRAD

EGVRVNILVWDDRTSMGAKGVMGTHDEILCPRNPDQGGTMFTHHQKTLIVDASMVGGLDL

CNGRYDTPNHPLFSTLHDIHNGDFHQNFENGGPREPWHDIHGRVEGEIAWDVLYNFEQRW

RKQAKLVPTDDSWNVQLFRSIDSGAVAARDRSIQDAYINAIRRARDFIYIENQYFLGSSR

DVQALHLVPMEIALKIVSKIEAGERFSVYVVIPMWPEGDPDAGAVQYILAWQHHTMQMMY

KRIVALHAVTPKDYLSFFCREPMIYVHSKMMIVDDEYIIVGSANINLWYEHMRFLIQHVT

AMGDKFWNQYTYPV-H

>MpPLD5

---------------------------------------------------------MPD

GFLEKISLADKQPTHCWEDAYRMIMGARHMIYVAGWSVYTEITLIRDEDTLGQLLKRRAD

EGVRVNILVWDDRTSLYSKGTMATHDNILCPRKTDKGGTMFTHHQKTLIVDASMVGGLDL

CNGRYDTPNHPLFSSLHDIHKGDFYQCFDNGGPRQPWHDIHGRVEGEIAWDVLYNFEQRW

RKQAKLVPTDDSWNVQLFRSIDSGAVAARDRSIQDAYINAIRRARDFIYIENQYFLGSAS

DVKAHHLVPMEIALKIVSKIMAGERFSVYVVIPMWPEGDPESNVVQYILFWQHCTMQMMY

KRIAALHAVTPKDYLSFFCREPMIYVHSKMMIVDDEYIIVGSANINLWYEHMRFLIQHVN

AMVDKFWNQYTYPV-H

>MpPLD6

FATVELVMARTRAEQIAWNECSGNIPTDILLK-RFFKGKIHVIVDFEPADEWGCLSHIED

DFIPPIAMQGRIPGKYWKELHHAICSAKKFVYIAGWSMFAKISLVRSPDTLGELLIRRAQ

EGIRVNVMIWDDKTSLKREGIMHTHDELKCPRDPRAEGFVFTHHQKVVVVDAAFVGGIDL

CGGRYDTPSHSLFRTLTTVHRKDFQQCIKSGGPRQPWHDIHSKLEGAVAWDAHTNFVQRW

LCQALLVSEAAAWNVQLLRSIDTTSVVLEDRSIQDAYICAIRRAKHFLFIENQYFIGSSK

DTAAPHLIPMEIALKIVSKIRAGEHFCCYVIVPMMPDGAPDGPSVQDILHHVKETMQMMY

QMIDALREVRPTDYLCLFCREPEIYCHAKLMIVDDEYLIIGSANINLWFEHLGFDMRKVQ

ELAAHNWQEYIYPFGI

>MpPLD7

---------------------------------------------------------MPD

GFLEKISLADKQPTHCWEDAYRMIMGARHMIYVAGWSVYTEITLIRDEDTLGQLLKRRAD

EGVRVNILVWDDRTSLYSKGKMATHDNILCKRRTDKGDTMFTHHQKTLIVDASMVGGLDL

CNGRYDTPNHPLFSSLHDIHNGDFYQCLENGGPRQPWHDIHGRVEGEIAWDVLYNFEQRW

RKQAKLVPTDDSWNVQLFRSIDSGSVAARDRSIQDAYVNAIRRARDFIYIENQYFLGSAS

DVKAHHVVPMEIALKIVSKIMAGERFSVYVVIPMWPEGDPESVTVQSILFWQHCTMQMMY

KRIAALHAVTPKNYLSFFCREPMIYVHSKMMIVDDEYIIVGSANINLWYEHMRFLIQHVN

AMGDKFWNQYTYPVLN

>PpPLD1

YVTVVLAVARTRVISPKWHESLGKIPVEQVLHGSQGRAQLKFSASYVPVEQYKCRTHIYD

GTLPNIRLDGYEPRRCWEDLCVAIHEAKYLIYIAGWSVYYKVKLIRDYNTLGELLKLKAK

QGVRVLLLVWDDKTSIKTDGVMNTHDEVLAPRYGAVVGTLYSHHQKMTIVDTSFIGGLDL

TGGRWDTPSHTLFSSLEREHKHDFRNSWDSGGPRQPWHDWHCKIEGHAAYDVLKNFEQRW

NKATELLDLEDTWQVQIFRSIDSGSVVTKDISIQMAYIKAIRSAQHFLYIENQYFLGSSK

TAGANHLIPMEIALKICSKIREGKRFSVYIVIPMWPEGVPDSSPVQEILYFQTQTMKMMY

SMIGALRDCKPTDYLNFYCREPMIYVHSKGMIVDDEYVISGSANINLWAEHLGFDVQRVN

DMAERNWQQYRYPLNI

>PpPLD10

IAIVELVVARTRAVKVKFEESSGKIPTEELLKGKDHEAKFHITLRFIQAVNYNCKAHIDE

TFSPKIKLDNYTPEKGWEAVYHAMNEAKKFIYVAGWSINATIALIRDKETFGELLVRKAN

EGVTVLMLIWDDKSN--LTGVMNSHDELLCPRKPQITGFIFTHHQKVVCMDAAFQGGIDL

CDGRYCYPAHPLFQHLDTLFKHDFHQSIVHGGPREPWHDCYSRLEGEIAWDVHENFRQRW

LKQALLVSNRDTWNVQLFRSLDETSGIFKERSIQDAYIQAIRRAKRFLYIENQYFLGSSR

DESCAQLIPLEIALKIASKIRAGEDFSVYVVNPLWPDGAPTSLSGQDIMLWHRKTLEMMY

RIIKALQDIDMTRYLSFYCREPMIYCHAKIMVVDDEYLIIGSANINTWFEHLGFQMQRVN

ELASRYWEEYRYPYHQ

>PpPLD11

RQERQFT--KTGLFLEYLKDKSGVKETVGLLAGEYTGSRIRFSLEFFEARRWGCRAHMAE

NFLPPINLSN-------------------------------ITLCRDPQTLGELLKKKAD

QGVRVHVMVWKDPTTLREQGFLSTHCKLRCRRKFDHTLS-FSHHQKTIIVDASFVGGLDL

CDGRYDNQVHSLFRTLDTAHRRDFYQKFECGGPREPWHDIHSKLEGPVAWDVLRNFEERW

IKQADLLPLGETWNVQVFRSIDAGAAAANDRSIHHAYINAIGRARNFIYIENQYFLGSS-

ETEAFNLIPMELVRKIVSKIEAGERFVVYIVIPMYPEGNPESRTVQEILGWQRRTFQMMY

KEILALTARHPKDYLSVFCREPMIYVHSKMMIVDDEYIIVGSANINLWYEHLGFLIRHVN

ESSDYLWNLFSYPYTS

>PpPLD13

YIKHFLLIVNTREVCPKLHEGLKGDPVFDVLPLDKERRTVRIRTQRAGRAKWVCHPHRFG

SFAPPRGF-SIDGKAAFEAIAGAIENARSEIFIAGWWLCPDLYLRRPYGRLDHLLEAKAK

MGVQIFILLYKEVALLKKQRLLGLHENIKVLRFPDSGVYLWSHHEKLVIVDHCFLGGLDL

CFGRYDTPDHRVSDHPSTIWGKDYYNRERRKIPRMPWHDVHCALWGPPCRDVARHFVQRW

RSKAMVIPQQEWWDVQVIRSVGQWSATSQERSIHEAYCSLIDKAEYFVYIENQFFIGLEG

DDTIHNRVLQALYSRIMRAYKEGRCFRVIVLLPLLPGGVDDGGAVRAIMHWQYRTICRGK

NSLERLSCEQAHDYISFYGHGPQIYVHSKIMIVDDWAVLIGSANINLWAEHLGLRTTEVD

AIRDDLWMSYFFALES

>PpPLD14

YLNHFLLIVNTVEVCPKLQEGLKADPLFDMLS--KERRELKFRTSRAVSARWVCNPHRFG

SYAPQRGMTAIDGRAAFDAIMMAIESAQSEIFLTGWWLCPELYLRRPFMRLDVLLESKAK

EGVQIYVLLYKEVSMLKKRRLQGIHENIKVLRWPDSGVYLWSHHEKLVIVDHCFLGGLDL

CYGRYDDPNHRVWDSPPSIWGKDYYNRERNKLPRMPWHDVQCAIWGPACRDVARHFVQRW

RNKAMVIPAADWWGVQVIRSVGQWSATTQERSIHAAYCSLIEKAEHFIYIENQFFIGMDD

DDTIRNRVLQALYVRIMRAHSEGKCFRVIVVMPLLPGGVDDAGAVRFIMHWQFRTICRGR

HSLHRLQASQANDYVSFYGHGPQIYVHSKLMIVDDRLAIIGSANLNLWSEHLGVDFSNIE

TIVDEVWMKYNFPLES

>PpPLD2

YATVDLTVGRTRVLEPEWNETVGRIPAIDLLSGKSTGARLRFSVQFTRAIEWGCRAHVTD

NFLPPIYLGHYQPCRCWEDMFDAIHNAKHIIYITGWSVYTEFKLCRDPQTLGELLKRKAD

QGVRVNLMVWDDRSS-GIMGQMATHDEFLCPRSGYKTVVMFTHHQKSLIVDASFVGGLDL

CDGRYDDQYHSLFRTLDTVHSTDFHNGFEYGGPREPWHDIHCKLEGPIAWDVLYNFEQRW

RKQALLSPRGETWNVQLFRSIDAGAAVAKDRSIQNAYIHAIRCAKNFIYIENQYFVGSSQ

EVGANNLIPMELVRKIASKIEAGERFSVYIVIPLYPEGYPSGDAVQAILRWQQKTFQMMY

KEINSLRLKHPKDYLSVFCREPMIYVHSKMMIVDDEYIIVGSANINLWFEHMGFLIRKVN

HRGDELWSMFTYPYSS

>PpPLD3

YATVDLTVGRTRVLKPVWNESVGKIPAESLLSGEGDESQIRFRMQFYEASHWGCKAHMED

GFLPPIYQSGRQPTHCWEDVFDAIMGARHLIYITGWSVYCETVLVRDPRTLGELLKKKAK

EGVRVNMLVWDDKTSMKRDGLMATHDEFLCPRSPDTIGTMFTHHQKTIIVDASFVGGIDL

CDGRYDNQNHSLFRTLNDVNMNDFHQNYAKGGPREPWHDIHARVEGPVAWDVLWNFEQRW

RMQARLIPIDDTWSVQLFRSIDAGAAIARDRSIQDAYINAIRRAKDFIYIENQYFLGSCQ

DAGAFHTIPMELTRKIVSKIEDGERFAVYVVVPMWPEGIPESGSVQAILDWQKKTMEMMY

TQINALRAQSPRDYLTFFCREPMIYVHSKFMIVDDEYTIIGSANINCWYEHIGFLIRKVN

RIADQHWEMFSYPIKS

>PpPLD4

YAAVNLAVARTRVISPQWNEHLGKIPVGDIMDGKSGKARIYFTMKFTPVEMYMCEAHIMD

GSLPQITLADYQHRQAWEEMCTAILDAHHLIYIAGWSIYTKIKLLRDTTCLGDLLKRKSA

EGVRVLMLVWDDKTSIKTVGVMGVHDEVLSPRYADVVGTLYTHHQKTVIVDSSFLGGLDL

CDGRWDTPTHSLFNTLSTFHKDDFHNTFEGGGPRQPWHDWHCKIDGPAAYDVLTNFEQRW

RKAAELIQISSTWRCQVFRSIDSGSVAEKDISIQMAYIKAIRSAQHFIHIENQYFIGSSK

DAGANHLIPMELALKVASKIREHKRFAVYVVIPMWPEGVPDSGAMQEILFFQAQTIKMMY

GVIDALRDVHPRDYLNFYCREPMIYVHAKGMVVDDEYIICGSANINLWSEHLGFTVRTVN

KIADENWKQYPYPINQ

>PpPLD5

YTVVVLAVARTRVINPKWNEHMGRIPAWLVINGGEGKTRLRIFTRYIPVEAYTCRAHVYD

NSLSNIMLDSYSHGHCWEDICTAINDARHLVYIAGWSVYHKITLVRDENTLGELLKKKAS

QKVRVLMLVWDDKSSLKTSGLMNTHDEILAPRYGAVVGSLYSHHQKTVIVDSSFIGGLDL

TGGRWDTPCHYPFASLEKEHKHDFRQSWESGGPREPWHDWHCKIEGHAAYDVLTNFEQRW

RKATELIDHDETWHVQLFRSIDAGSVVQKDISIQMAYIKAIRSAQHFIYIENQYFLGSSP

TAGANHVIPMELALKICSKIREGKRFAVYVVIPMWPEGIPDSGPVQEILFFQSQTMKMMY

ATIETIRECKPTDYLNFYCREPMIYVHAKGMIVDDELVILGSANINLWAEHLGFEVQRIN

YIADMNWEQYRYPLNQ

>PpPLD6

YATVDLTVGRTRVLAPVWNEKVGKVPVLDLLSGEHTGSRIRFSLQFFEASRWGCHAHMAE

NFLPPIYLSGYQSRRCWEDIFEAINNAQKLIYITGWSVYTEIRLCRDPQTIGELLKKKAD

QGVRVNVMVWDDRSSLRQQGVMSTHDEFLCPRDADQIGGLFTHHQKTVIVDASFVGGLDL

CDGRYDNQFHSLFRTLDTAHSRDFHQVFECGGPREPWHDIHSKLEGPVAWDVLSNFEERW

KKQADLLPLGETWNVQVFRSIDAGAAAANDRSIHHAYINAIRRARNFIYIENQYFLGSSK

EAGAFNLIPMELVRKIVSKIEAGERFAVYVVIPMYPEGAPESQSVQAILDWQRRTFQMMY

KEIVALNARHPREYFSVFCREPMIYVHSKMMIVDDEYIIVGSANINLWYEHLGFLIRHVN

ERSDDLWNLFSYPYTS

>PpPLD7

YAIVVLAVARTRVISPEWKERVNKIPVEVVLNGGQGKAKLRLSVKYFPVEQYMCRAHIYD

NTLPSIALEGYVQNRCWEDMCTAINDAQHLIYIAGWSVFDKVTLVRDVNTLGELLKKKAS

EKVRVLMLVWDDKTSFKTAGVMNTHDEILAPRYGAVVGTLYSHHQKITIVDSSFIGGLDL

TGGRWDTPTHSLFASLQDEHKYDFRNSWGSGGPREPWHDWHCKIEGHAAYDVHTNFVQRW

RKATDLIDREETWHVQVFRSIDSGSVVQKDISIQMAYIKAIRSAQHFVYIENQYFLGSST

TAGANHLIPMELTLKICSKIREGKRFAVYVVVPMWPEGIPDSAPVQEILFFQSQTIKMMY

AIIETIRDTKPTDYLNFYCREPMIYVHAKGIIVDDELVIMGSANINLWAEHLAFEVQRIN

YIADRGWEQYRYPLNQ

>PpPLD8

YATVDLTVGRTRVLAPVWNETVGKLPVIELLSGQHFGAGIRFSLQFFEANRWGCHAHMTN

NFLPPIYLGDYQPHRCWEDIFEAINNAQRLIYITGWSVNTEIKLCRDPWTIGELLKKKAD

QGVRVNVMVWDDRSSLRQTGVMSTHDEFLCPRDADQIGGLFTHHQKTVIVDASFVGGLDL

CDGRYDDQYHSLFRTLDTAHNQDLHQVFACGGPREPWHDIHSRLEGPVAWDVLYNFEQRW

KKQADLLPL-ETWNVQVFRSIDAGAAAAEDRSIHDAYINAIRRARNFIYIENQYFLGSSQ

EAGAFNLIPMEIIRKIVSKIEAGERFCVYIVIPLYPEGVPDTQSVQAILDWQRRTFQMMY

KEIEALSAQHPREYLTVFCREPMVYVHSKMMIVDDEYIIVGSANINLWFEHLGFLIRQVN

EIADKNLKLFAYPYTA

>PpPLD9

ATTLLLVLLLS--IGSLEKEDVQSIPVPGVLNGDQGNKGLDVSAQY-----WE-------

---PINPISG--------DYGFSEQEMAHFGAPVGKAVY-------------DSLLAAAD

RGVP-----------LQHTGFSPSFDA----------------HSKLWIANKAYLGSA--

---NND------------------------------WKSFTQVKEGVYFKLLDGYFNNFW

--NAVVWD--QQWQLQFSKYTTTSHVLDTHQTDEQGWLDTIFSVPVNVRINTQFL-DMD-

---------------FSNAISKVKHATVKVLVARWKT-------TDKYL-----------

---------LYLNYTNFLCKSPARVNHAKFA-VSDVRANIGTSNL-VWDYYAGFGVSQLQ

EIFDADWTSYAVPLSL

>PsPLD10

YLKHFLLIVNSREVCPKLHEGLKGDPVFDVLPLDKERRSVRIRTQRAGRAKWVCHPHRFG

SFAPPRGF-SVDGKAAFEGIATAIENARSEIFIAGWWLCPDLYLRRPFSRLDHLLEAKAK

MGVQIFILLYKEVALLKKQKLLGLHENIKVLRFPDSGVYLWSHHEKFVIVDHCFLGGLDL

CFGRYDSPEHRVSDYPSATWGKDYYNRERQKVPRMPWHDVHCALWGPPCRDVARHFVQRW

RSKAMVIPQSEWWNVQVIRSVGQWSATSQERSIHEAYCSLIEKAEYFVYIENQFFIGLEG

DDTISNRVLQALYSRIMRAYKEHRCFRVIVVLPLLPGGVDDGGAVRAIMHWQYRTICRGK

NSLERLSSEQADDYISFYGHGPQIYVHSKVMIVDDWSVLIGSANINLWAEHLGLRANEVN

AIRDDLWMAYNYPMES

>PsPLD11

YLNHFLLIVNTIEVCPKLQEGLKADPVFDVLG--KDRRELTFRTGRAVNARWVCNLHRFG

SYAPQRGLTLVDGKAAFEAIAMAIESAQSEIFLTGWWLCPELYLRRPFKRLDFLLESKAK

AGVQIYVLLYKEVSMLKKRRLLGIHENIKVLRWPDSGVYLWSHHEKLVIVDHCFLGGLDL

CYGRYDDPCHRVWD-PPSIWGKDYYNRERTKLPRMPWHDVQCAIWGPACRDVARHFVQRW

RNKAMVIPATDWWRVQVIRSVGQWSATTLERSIHAAYCSLIENAEHFVYIENQFFIGMDG

DDTIQNRVLEAMYSRIMRAHREGQRFRVIVVIPLLPGGVDDSGAVRFIMHWQFRTICRGR

SSLQRLQDSQARNYVSFHGYGPQVYVHSKLMIVDDRFVLIGSANINLWSEHLG-------

----------------

>PsPLD5

YTVVVIAVARTRVINPKWKEHMGSIAAKLLLHGGEGKAKLRLSARYIPVEQYSCRAHVYD

HTLPKIVLDGYVHGLCWEEICTSINDAQHLVYVAGWSVWDKVKLVRDVNTLGELLKKKAS

QGVRVLVLAWDDKSSVKTAGVMNTYDEVLAPRYGAVVGSLYTHHQKSVIVDSSFIGGLDL

TAGRWDTPSHYLFASLQNEHKGDFRNSWESGGPREPWHDLHCKIEGPAAYDVLTNFEQRW

RKATELLDHEETWNVQVFRSIDSGSVVQKDVSIQMAYINAIRSAQHFIYIENQYFLGSSK

SAGANHLIPMELSLRICSKIREGKRFAVYVIVPMWPEGVPNSGAVQEIFHRQ--------

------------------------------------------------------------

----------------

>PsPLD6

YAAVNLALARTRVISPNWDEHLGNIPVERLIHGENGKAKIWFKMKFTSVEKYYGRAHIMD

GSLPEIELEGFHHRQAWEELCTAILEAHHLVYITGWSVYTKVRLLRDTRILGDLLKRKSA

EGVRVLLLVWDDKTSIKTEGMMGVHDEVLAPRYADVVGTLYTHHQKCTIVDTSFIGGLDL

CDGRFDTPSHSLFSTLTTFHKNDYHNTFEAGGPREPWHDWHCRIDGPAAYDVLTNFEQRW

RKATELIQISESWHVQVFRSIDSGSV----------------------------------

------------------------------------------------------------

------------------------------------------------------------

----------------

>PsPLD7

YATVDLTVGRTRVLEPVWNETV----------GRHTGARIRFSLQFFEASHWGCKAHMAD

HFLPNIYLANYQPTRCWEDIFEAINNARQLIYITGWSVYTEFKLCRDPHTLGELLKRKAD

QGVCVNIMVWDDRSSLRQQGQMRTHDEFLCPRNPTQIGNLFTHHQKSVIVDASFVGGLDL

CDGRYDTQYHSLFRTLNGAHQQDLHAVFECGGPREPWHDIHSKLEGPVAWDVLYNFEQRW

RKQAALLDNGETWNVQMFRSIDAGAAAALDRSIQQAYIHAIRRARNFIYIENQYFIGSAQ

ESGAYNLIPMELARKIVSKIEAGERFTAYIVIPLYPEGIPDSVPVQEILAWQRRTFKMMY

REINAFRAKHPREYLSVFCREPMIYVHSKMMIV---------------------------

----------------

>PsPLD8

------------------------------------------------------------

------------------------------------------------------------

---------------------MNTHDEVLAARYGAVVGTLYSHHQKMTIVDSSFIGGLDL

TGGRWDTPSHCLFGSLDNEHKNDFRNSWDSGGPRQPWHDWHCKIEGHAAYDVLTNFEQRW

KKATELLDHGETWQVQIFRSIDSGSVVTKDISIQMAYIKAIRCAQHFIYIENQYFLGSSK

TAGANHLIPMELTLKICSKIKANQRFAVYIVIPMWPEGVPEGGPVQEILYFQTQTMKMMY

GLIDTLRDCKPTDYLNFYCREPMIYVHSKGMIVDDEYVISGSANINLWAEHLGFEVRRVN

DMSXRNWEQYRYPLNQ

>PsPLD9

YATVDLTVGRTRILQPVWNETVGRVPVIDLLSGRHTKARIRFSLQFFEAARWGCRAHMAD

NFLPPIYLDNYQPHRCWEDIFESIYNARHLIYITGWSVFTEIKLCRDPNTLGELLKRKAD

QGVRVNVMVWDDRSSLRQVGVMSTHDEYLCPRNADQIGGLFTHHQKSVIVDASFVGGLDL

CDGRYDTQYHSLFRTLTTTHSADFHQVFACGGPREPWHDIHSRLEGPVAWDVLYNFEQRW

RKQAALLPLGETWNVQVFRSIDAGAAAANDRSIQHAYINAIRRARNFIYIENQYFLGSSQ

QSGAYNLIPMELVRKIVSKIEAGERFTVYIVIPVYPEGIPESQSVQAILAWQKKTFQMMY

KEIIALSSKHPRDYLTVFCRETMIYVHSKMMIVDDEFIIVGSANXNLWFEHLGFLIRTVN

QRADELWGLFAYPYTS

>ScPLD1

YATVEMAVARTRVLDPIWNETVGLIPAAQIVSGEDGRSKLHLRMQYYDVSSVGCNAHVPD

EFCPPI--GNYQPRRCWVDIYDAIAGAKHLVYIAGWSVFADVELIRDDETLGELLKKKAL

EGVRVNLLVWDDRTSWKKDGLMATHDEVLCPRNPDQIGAMFTHHQKIVVVDASFIGGLDL

CDGRYDTPSHSLFRTLNSIHKSDFHQNFKKGGPREPWHDIHCRIEGAAAWDVWRNFEQRW

RKQGKLVEVTGSWNVQVFRSIDGGAAAAMDRSIQDAYISAIRRAKSFVYIENQYFLGSCQ

ECGATHLIPMEIALKIASKIAAGESFCAYIVVPMWPEGVPESASVQAILGWMHKTMAMMY

RIVRALDAKNPRDYLAFYCREPMIYVHSKMMIVDDEYIIVGSANINLWYEHLGFMMRRVN

GVAEQLWDLFKYPVAK

>ScPLD2

YASIEMTVARTRVLKPVWNESLGKIPVADILTGERDEAKIHLKMQFFDINGFGCKAHIPN

NFNPKIYLSNYQVQRCWEDIYRAISGAKHFIYITGWSVYAEIELIRDPDTLGELLKKKAK

EGVRVNMLVWDDRTSLRKDGLMCTHDQILCPRNPDQIGTMFTHHQKTVIVDASFVGGIDL

CDGRYDTPFHPLFRTLDTVHKSDFHQNYAKGGPREPWHDIHSKLEGQAAWDVLHNFEQRW

QKQGHLLTISSSWNVQVFRSIDAGAAAAKDRSIQDAYIHAIRRAKNFIYIENQYFLGSCQ

ECGATHLIPVELALKIASKIEADERFTVYVVVPMWPEGVPESGSVQAILDWMHKTMEMMY

KIISALEAKSPRDYLTFFCREPMIYVHSKMMIVDDEYIIVGSANINLWYEHLGFQVQRVN

TIADELWDKFTYPVKG

>ScPLD3

YVCLILSLARTNVISPEWEQHIGKLPAEDILSKPYGQAQLTISLSYRPIEQYKCKAHVPR

GLLPRIDLADFEQRNCWHDICKAIVDAKHLVYIIGWSIYTKITLVRDHENLGELLKWKAG

RDVRVLLLVWDDKTSVRNEGVMGTHDELLAPRYADIVGTIYTHHQKVVIVDTAFIGGLDL

CDGRYDTPEHTLFSKLHTDFKDDYHNTFDSGGPREPWHDLHCKIEGPAVYDVLENFEQRW

RKVEGLLDATQKWYIQVFRSIDSGSVARQDMSIQAAYVKAIRSAQHFVYIENQYFIGSSK

NAGADNLVPMELALKIVSKIKAHKDFAVYVVIPMWPEGDPISLSVQEILFFQWQTMEMMY

TLIEALKEEHPTDYLNFFCREPTIYVHSKGMIVDDEYVVIGSANINLWAEHLGFCVHAVQ

HISRENWKKFPYPVST

>ScPLD4

YASIEMIVARTRIIDPVWNESVGIFSVSEILSGEEGMARVHLRMRYIDISRLGCKAHVSS

RFCPKVLLSNYEPHRCWEDIYLAIRGAKHLIYIAGWSVYTRIELIRDREQLGELLKQKAA

EGVVVNMLVWDDRTSLKRDGLMATHDEILCPRNPDQIGTMFTHHQKIVVVDASFVGGIDL

CDGRYDTQCHSLFRTLYSFHKDDFHQNFRLGGPREPWHDIHCKLEGQVAWDVLHNFEQRW

QKQGKLLNISSSWNVQLFRSIDGGAAAANDRSVQDAYIHAIRRAKRFIYVENQYFIGGSR

DSGCSHLIPIEIALKIASMIEAGEQFTVYIVVPMWPEGVPESGSVQAILHWMHRTMEMMY

RIVRALEAKNLREYLTFFCREPMIYVHSKMMIVDDEYIIVGSANVNLWYEHLGFLIREVN

SIADYLWDQFTYPVKT

>ScPLD5

YACVVLAVARTRVISPRWEEHMGQLPAESLLSKENNEMHLLISVKYVSVSEYKCHAHVPD

GLLPPIELKGYEQGKCWEDICQAIVDARHLVYIAGWSIYTKTRLMR---SLGELLKAKAN

GTARVFLLVWDDKTSIGNVGVMGTHDELLAPRYGAMVGTLYTHHQKTVIVDAAFLGGLDL

CDGRYDTPEHTLFKNLNSVFVEDYHNTFESGGPRQPWHDLHCKIEGPAAYDVLKNFEQRW

HKAAMLVDVPETWHVQIFRSIDSGSAMGEDMSIHTAYVTAIRSAQHFIYIENQYFLGSSQ

DAGANHLVPIELALKIVSKIKANERFA---------------------------------

----------------------MIYVHAKGMIVDDEYVILGSANINLWYEHLHFKVRKVR

SLAETNWVHYKYPIQT

>ScPLD6

YLNHFLLIVNTKEVCPKLREGLKSDPVFDVIPSDKERRSIRIRSKRAGIARWVCHPHRYG

SFAPPRGINNIDGKAAFNAMASAIERAKSDVFIAGWWLCPELYMRRPFVRLDTLLQERAQ

MGVKIYILLYKELALLKKRRLLTIHENVKVLRFPNAGVYLWSHHEKIVIVDHCFLGGLDL

CFGRYDTQEHRVHDFPSRIWGKDYYNRERQRYPRMPWHDVHCAIWGPACRDVARHFVQRW

RCKAMVLPQQEWWQDQVLRSVGLWSATSQETSIHAAYCSLIDNAEYFVYIENQFFIGFDD

DDVIQNRVLQALYNRILQAHKEGKTFRVVVVIPLLPGGVDDAGAVRAVMHWQYRTISRGP

CSLQRLRKADTENYVSFYGYGPQVYVHSKIMIVDDRAVLVGSANINLWKEHLGLGQSEIQ

SLMDQIWMAYCFPLES

>ScPLD7

LPLILLILCSSARIS------VESIPVPGLLSGEQGNQKLDLLVQY-----WQ-------

---PNNSQSG--------DYGYSGDDMQRFGASIGQTVF-------------RFLDAAAA

RGVD-----------IQHSGVFPDYDQ----------------HAKVWIADQLYIGSA--

---NND------------------------------WKSLTQVKEGVYMKIVEGYFENLW

--NAHVWD--EQFQLKLPNAIKETDDLLTHPTDEKGWVETILSAPLNIRINTQYL-GTT-

---------------ISTAVSKVKNVTVRVIVAYWTH-------TNEYL-----------

---------TALNYTNILCTSPTRVNHAKYV-VSDLRVHIGTSNL-VWDYYTGFGVSQLQ

AVFDADWESYAVPWAS

>SfPLD1

YVIVVLAVARTRVISPKWDEHLGKIPVEQLLDGDNGKAKLHLRVSYQPVEKYTCDTHIYD

STLPRIWLESYHQHRCWEEMCAAIAGAHHLVYLAGWSIYDKIKLVRDINTLGELLKKKSA

DGIRVLLLQWDDRTSLKTNGVMNTHDEILAPRYADVVGTLYSHHQKVVIVDTSFIGGLDL

TGGRWDTPSHLLFSSLQNEHKGDFRNSFEGGGPREPWHDWHCRIDGHAAYDVLTNFEQRW

RKATELLDIPETWHVQVFRSIDSGSVVQQDVSIQMAYIKAIRSAQHFIYIENQYFLGSSD

KAGANHLIPMELALKICSKIRAGQMFAVYVVVPMWPEGVPESAPVQEILYFQSQTMKMMY

KLIEALRDCHPRDYLNFYCREPMIYVHAKGMIVDDEFVIVGSANINLWAEHFGFEVRRVN

ALAESNWQQYPYPLNQ

>SfPLD10

MQLLLLLVCLSAAKH------VQSIPVPGVLRGDQGNKSLDIMSYY-----WE-------

---PKNPASG--------DYGYSQQQLDQFGAPVGQAVY-------------DSLIAAAD

RGVP-----------VQDTGFSPDFDS----------------HAKLWISNKVYVGSA--

---NND------------------------------WKSLTQVKEGIYFKRLEHLFENYW

--NATVDD--KEWQIKLPAYVSTPRVLNTYQTDEQGWVDTILSVPVNVRINTEYT-NTI-

---------------LSSAIAKVKHATVKVIVAHWAY-------TEQYL-----------

---------LFLNYTNALCSSPTRVNHAKYV-VSDVRANIGTSNL-LWDYYNGFGVKQLQ

AVFESDWNSYAVLLNA

>SfPLD11

IATVELVVARTLSASVSWHESSGKVASESLLSGEDHEAKIHLIMQFQAAKLWNCKAHIDD

SFSPNIMLETYQPGKAWEDLYWALDHAKHFIYIAGWSVNARISLVRDPETLGSLLIRKAN

QGVSVLCLVWDDKTDLDRPGIMHSHDELLCPRNPDAEGFIFTHHQKVVVMDYAFQGGIDL

CDGRYDTPSHPLFKHLTTLFKNDFAQSIKLGGPREPWHDGYSKLEGSIAWDVHTNFTQRW

LKQAFLVPDTEAWNVQLFRSIDDTSVILKDRSVQDAYIQAIRRAKRFLYIENQYFLGSSK

DDSATQLIPLEIALKIVSKIHAGEPFSVYIVNPLWPHGSPTSTSGQDICLWHRKTLEMMY

RMIKALKDTHPTDYLGFFSREPMIYCHAKMMVVDDEYVIIGSANINMWFEHLAFNMKRVT

ELATQYWEEYRYPYGK

>SfPLD12

------------------------------------------------------------

-------------------MCAAIAGAHHLVYLAGWSIYDKIKLVRDINTLGELLKKKSA

AGIRVLLLRWDDRTSLKTSGVMNTHDEILAPRYADVVGTLYSHHQKVVIVDTSFIGGFDL

TGGRWDTPSHLLFSSLQNEHKGDFRNSFEGGGPREPWHDWHCRIDGHAAYDVLTNFEQRW

RKATELLDIPETWHVQVFRSIDSGSVVQQDVSIQLAYIKAIRSAQHFIYIENQYFLGSSD

KAGTNHLIPMELALKICSKIRAGQMFAVYVVVPMWPEGVPESAAVQEILYFQSQTMKMMY

KLIEALSDCHPRDYLNFYCRVPMIYVHAKGMVVDDEFVIVGSANINLWAEHFGFEVRRVN

ALAESNWQQYPYPLNQ

>SfPLD17

YLKHFLLIVNTREVCPKLREGLKVDPVFDVLVSDKERRVVKFRTQQARSARWVCHPHRFG

SFAPPRGLGSIDGKAAFEAIAVAIDDAKSEIFLAGWWVCPDLYLRRPFSRLDTLLEAKAK

SGVQIYVLLYKEVALLKKQRLLSIHENIKVLRYPDSGVYLWSHHEKLIIVDHCFIGGLDL

CFGRYDSPEHRVSDYPVSLWGKDYYNREREKYPRMPWHDVHCALWGPPCRDVARHFVQRW

RSKAMVIPQVEWWEVQVIRSVGQWSATSQEHSIHTAYCSLIEKAEFFVYIENQFFIGLDS

DDIIHNRVLQALYTRIMRAHEDRRCFRVIVVMPLLPGGVDDSGAVRAIMHWQYRTICRGR

QSLQRLLDRDLENYISFYGHGPQIYVHSKIMIVDDRTVLIGSANINLWAEHLGIRQAEID

LVKDGVWMSYFFPLES

>SfPLD18

YLNYFLILVNTREVCPKLREGLKADPMFDVLPMPKEKRAVRMWTSWAAGARWVCYPHRFQ

SFAPERGRTHIDGKAAFDAMAMAIEDAETEIFITGWWLSPELYLRRPFKRLDHLLEAKAK

MGIKIYILLYKEVSMLKKQRLLALHENIKVLRWPNSGVYLWSHHEKLVIVDNCFLGGLDM

CFGRYDDPTHPVCDSPSTIWGRDYYNRERNKLPRMPWHDVQCAIWGPPCRDIARHFVQRW

QSKAMVIPQAQCFD-QVIRSVSHWSATSQERSIEAAYCELIEKAEYFIYIENQFFIGLDG

DETIQNRVSQALYARIVRAYNKGLVFRVIVVIPLLPAGLDEVGAVRFIMHWQYRTICHGK

HSLEKLKETRVEDYISFYGHGPLVYVHSKLMIVDDRFVLTGSANINLWAEHLGLQHSELD

CIKDDIWMSYNFPLET

>SfPLD19

YLNHFLLIVNTREVCPKLREGLKADPLFDVLPTMKEKRAIKMRTAWATGARWVCHPHRFQ

SFAPERGITDVDGKAAFEAIALAIEDARTEIFITGWWLCPELYLRRPFHRLDCLLESKAK

MGVRIYILLYKEVSMLKKQRLLAIHENIKVLRWPDSGVYLWSHHEKLVIVDNCFLGGLDM

CFGRYDDPEHRVSDNPPTIWGKDYYNRERNKFPRMPWHDVHCAIWGPSCRDVARHFVQRW

RNKAMVIPASEWFE-QVIRSVGHWSATSQEQSIHAAYCSLIEKAENFIYIENQFFIGLED

DETIQNRVLQALYTRIMRAHKEAQVFRVIVVMPLLPGGVDDVGAVRLIMHWQYRTICKGK

HSLQKLEEKKVEDFISFYGHGPQVYVHSKLMIVDDRFVLIGSANINLWAEHLGLQHSELQ

CIKDEIWMLYNFPLES

>SfPLD2

YAIVILAVARTRVINPKWNEHLGKIPIEQVLNGDKGEAKLHVSVHYLPVEKYTCKTHIYD

NTLPEVYLDGYRHKQCWEEMCTAIMEACHLVYIAGWSVYDKVKLIRDRNTLGELLKKKAG

EGVCVFLLIWNDKTSLKTDGIMKTHDEVLAARYGDVVGTLYSHHQKLMIVDSSFIGGLDL

CDGRWDTPLHHLFNSLQNEHKDDFYNSFEAGGPRQPWHDWHCKIEGPASYDILTNFEQCW

RKATELVNSGESWHVQVFRSIDGGSVVQTDVSIQMAYVKAIRMAQHFIYIENQYFLGSSD

VAGANHLIPMELTLKICSKIRAGQRFAVYIVVPMFPEGIPDSGPVQEILYFQSQTMKMMY

FLIDAIRDSHPQDYLNFFCREPMIYVHSKGMIVDDELVILGSANINLWAEHLGFLVHRVN

QMAEANWLQYVYPLNQ

>SfPLD3

YAYVVLAVASTRLIIPRWNEHVGRIPAEEVLKKQSGKAKLRFKIILHPIDRFMCRAHVFD

GELPDVILENFEHRRCWEEMCTAILEARYMVYIAGWSVYVKVRLIRDSSTLGELLKRKAA

EGVRVLVLAWDDKTSFKTEGLMGVHDEVLAPRYADVVGTLYTHHEKMLIVDASFVGGLDL

CDGRWDTPTHSLFATLKTFHKNDFHNTFDAGGPREPWHDLHCKTEGPAAYDVLTHFEQRW

RKDAELIKIPSTCHAQVFRSVDSGSVVERDVSIQMAYVKAIRSAQHFIYIENQYFIGSSK

NAGANHVIPMELALKVAAKIRDSQRFAVYVVIPMWPEGIPATAALQEILFFQMQTMKMMY

GVIAALRDVHPRDYLNFYCREPMIYVHSKGMIVDDEYIICGSANINLWAEHLGLRVLRVN

EMAQANWEQYPYPISQ

>SfPLD4

YATVDLAVGRTRVLKPVWNESVGKVPAADLLSGAEEQSRIRFSLRFSEASRWSCKSHMPD

GFLPRIYQTSRQPTRCWEDVFEAISNARFLIYITGWSVYTEITLVRDPRTLGELLKKKAN

EGVTVNLLVWDDRTSVHPAGIMGTHDEFLCPRNPDTIGTMFTHHQKTIIVDASFVGGIDL

CDGRYDTQYHSLFRTLNGVHSNDFHQNFKHGGPREPWHDLHSRVEGPIAWDVLFNFEQRW

RKQALLRPIPDTWHVQFFRSIDAGAAAAKDHSIHDAYINAIRRARDFIYIENQYFLGSCQ

DAGALHLIPMELTLKIVSKIEAGERFTVYVVLPMWPEGVPHSGSVQAILHWQRRTMEMMY

KQIEALRIHTPKDYLTFFCREPMIYVHAKMMIVDDEYIMIGSANINLWFEHMGFLIRKVN

SIAERHWDLFTYPVLS

>SfPLD5

YATVDLAVGHTSILKPVWNESIGKVPAFELLSGRAGESRIRFYIQFFEACRWGCQVHMFD

NFMPPIYLAGQQPTRCWEDIFEAISNAEHLIYITGWSVFTEITLIRDPQTLGQLLKKKAD

QGVRVNLLVWDDRTSFNQIGLMATHDEYLCPRVPDTIGTMFTHHQKTVITDASFVGGIDL

CDGRYDTQYHSLFHTLNNVHANDFHQNFEFGGPREPWHDIHCKLEGPIAWDVLFNFEQRW

QKQALLIPIPDTWHVQLFRSIDAGAAAAKDRSIQDAYINAIRRAKNFIYIENQYFLGSCQ

DCGALHLIPMEITRKILSKIEVDERFTAYIVVPMWPEGVPESASVQAILDWQRRTMEMMY

REIIALQVKHPKDYLSFFCREPMIYVHAKMIIVDDEYIIVGSANINLWFEHMGFLVRKVN

QRSNELWDLFTYPLPS

>SfPLD6

YATVDLAVGRTRVLKPVWNESLGKVPAEELLSESNDNSRIRFRLQFFEASHWGCKSHMAD

GFLPPIYQAGRQPTHCWEDVFEAISNARHLIYITGWSVYTQILLVQDPRTLGELLKKKAD

EGVRVNLLVWDDKTSLHETGLMATHDQYLCPRNPDTIGTMFTHHQKTIIVDASFVGGIDL

CDGRYDNQHHSLFRTLNGVHNKDFHQNFEKGGPREPWHDIHCRIEGPVAWDVLFNFEQRW

QKQALLIPIPDTWHVQLFRSIDAGAAAAKDRSIQDAYINAIRRAKDFIYIENQYFLGSCQ

DAGALHLIPMELTRKIVSKIEAGERFAVYVVVPMWPEGVPNSGSVQAILDWQRRTMEMMY

KEINALNAQSPKDYLTFFCREPMIYVHAKMMIVDDEYIIIGSANINLWYEHMGFLIRKVN

RIGDQLWDLFTYPVLS

>SfPLD7

YASVVLAVARTRVISPTWDEHLGTIPVEQVIDGQSGNAQCRFKLFFTPVQNFTCDAHIVD

GSFPEVMLEDFQQHRCWEELCTAILDAHHMVYIAGWSIYHQVKLLRDTSTLGELLKRKSA

EGVRVLVLAWDDKTSIKNEGLMGVHDEVLAPRYGDVVGTLYTHHQKLIIMDTSFIGGLDL

CDGRWDTPTHSMFATLQTLHKNDYHNTFEAGGPRQPWHDWHCKIEGPAAYDILTNFEQRW

KKATELIQISNTWHVQVFRSIDSGSAAEKDISIQMAYIKAIRSAQHFVYIENQYFIGSSA

NAGANQLIPMELALKIASKIKAGERFAVYVIIPMWPEGVPDSAAVQEILYFQAQTMKMMY

GVINALREMHPQEFLNFYCREPMIYVHAKGMVVDDEYVICGSANINLWAEHLGFTVQRVN

EMAQANWEQYPYPVQQ

>SfPLD8

YASVMLAVASTRVISPHWNEHLGMIPAEEVLEKQSGKAKLHLKIIYHPIDTFKCRAHAFD

GELPDVILENLEHRCCWEEMCTAILKARHMVYIAGWSVYVKVRLIRDVSTLGDLLRRKAA

EGVRVLVLAWDDKTSFKTEGLMGVHDEVLAPRYADVVGTLYTHHQKVLIVDTSFVGGLDL

CDGRWDTPTHSPFVTLQTTHKNDFHNSFDGGGPRQPWHDLHCKIEGPAAYDVLTNFEQRW

RKATELIQISSAWHAQVFRSIDSGSVIEQDVSIQMAYVKAIRSAQHFIYIENQYFIGSSK

TAGANHLIPMELALKVASKIRDHQPFAVYVVIPMWPEGVPDSGPMQEILFFQTQTMKMMY

GVIAALSDVHPCDYLNFYCREPMIYVHSKGIIVDDEYIICGSANINLWAEHLGFRVRRVN

ELAQANWEQYRYPINQ

>SfPLD9

YATVDLAVGRTRVLKPVWDESVGKVPAAELLSGANGDSRIRFSLQFFAASDWGCNSHMAD

GFLPTIYQTGRQPTHCWEDVFKAITDARYLIYVIGWSVTTSITLVRDLQTLGELLKKKAD

EGVTVNLLVWDDKTSIHKDGVMGTNDQFLCPWNPDEIGTMFTHHQKLLVVDASFVGGIDL

CLGRYDTQHHPLFSTLNTVHSKDFHQNFKYGGPREPWHDIHSRLEGPVAWDVLFNFEQRW

KKQALLLSIPDTWNVQFFRSIDAGAAAAKDHSIHDAYINAIRRAKDFIYIENQYFMGSSQ

DAGALHLIPAELALKIVNKIEAGERFSVYIVVPMWPDGVPESGTMQAMLHWQNRTMQMMY

KQIDALRIHSPKDYLSFFC----IWRH------------------K--------------

----------TYPV--

>SmaPLD1

YATVDLAVGRTSILKPVWNESIGKVPAFELLSGRAGEARIRFYLHFFEASRWGCRVHMFD

NFLPPIYLAGQQPTRCWEDIFEAISNAQHLIYITGWSVFTEITLIRDPRTLGQLLKKKAD

QGVRVNLLVWDDRTSFNQIGLMATHDEYLCPRNPDTIGTMFTHHQKTVITDASFVGGIDL

CDGRYDTQYHSLFHTLNNVHANDFHQNFEFGGPREPWHDIHCKLEGPIAWDVLFNFEQRW

QKQALLIPIPDTWHVQLFRSIDAGAAAAKDRSIQDAYINAIRRAKNFIYIENQYFLGSCQ

DCGALHLIPMEITRKILSKIEVGERFTAYIVVPMWPEGVPESASVQAILDWQRRTMEMMY

REIIALQVKHPKDYLSFFCREPMIYVHAKMMIVDDEYIIVGSANINLWFEHMGFLVRKVN

QRSNELWDLFTYPIPS

>SmaPLD10

IATVELVVARTLSASVSWHESSGKVASESLLSGEDHEAKIHLIMQFQAAKLWNCKAHIDD

SFSPNIMLETYQPGKAWEDLYWALDHAKHFIYIAGWSVNARISLVRDPETLGSLLIRKAN

QGVSVLCLVWDDKTDLDRPGIMHSHDELLCPRNPDAEGFIFTHHQKVVVMDYAFQGGIDL

CDGRYDTPSHPLFKHLTTLFKNDFCQSIKLGGPREPWHDGYSKLEGSIAWDVHTNFTQRW

LKQAFLVPDTEAWNVQLFRSIDDTSVILKERSVQDAYIQAIRRAKRFLYIENQYFLGSSK

DDSAVQLIPLEIALKIVSKIHAGEPFSVYIVNPLWPHGSPTSTSGQDICLWHRKTLEMMY

GMIKALKDTNPTDYLGFFSREPMIYCHAKMMVVDDEYVIIGSANINMWFEHLAFNMKRVT

ELATQYWEEYRYPYEK

>SmaPLD11

YLNHFLLIVNTREVCPKLREGLKADPLFDVLPTMKEKQAIKMRTAWATGARWVCHPHRFQ

SFAPERGITDVDGKAAFEAIALAIQDARTEIFITGWWLCPELYLRRPFHRLDRLLESKAK

MGVRIYILLYKEVSMLKKQRLLAIHENIKVLRWPDSGVYLWSHHEKLVIVDNCFLGGLDM

CFGRYDDPEHRVSDNPPTIWGKDYYNRERNKFPRMPWHDVHCAIWGPSCQDVARHFVQRW

RNKAMVIPASEWFD-QVIRSVGHWSATSQEQSIHAAYCSLIEKAENFIYIENQFFIGLED

DETIQNRVLQALYTRIMRAHKEAQVFRVIVVMPLLPGGVDDVGAVRFIMHWQYRTICKGK

HSLQKLEEKKVEDFISFYGHGPQVYVHSKLMIVDDRFVLIGSANINLWAEHLGLQHSELQ

CIKDEIWMLYNFPLES

>SmaPLD12

YLKHFLLIVNTREVCPKLREGLKVDPVFDVLVSDKERRVVKFRTQQARSARWVCHPHRFG

SFAPPRGLSSIDGKAAFEAIALAIDDAKSEIFLAGWWVCPDLYLRRPFSRLDTLLEAKAK

MGVQIYVLLYKEVALLKKQRLLSIHENIKVLRYPDSGVYLWSHHEKLIIVDHCFIGGLDL

CFGRYDSPEHRVSDYPVSLWGKDYYNREREKYPRMPWHDVHCALWGPPCRDVARHFVQRW

RSKAMVIPQVEWWEVQVIRSVGQWSATSQEHSIHTAYCSLIEKAEFFVYIENQFFIGLDS

DDIIHNRVLQALYTRIMRAHEDRRCFRVIVVMPLLPGGVDDSGAVRAIMHWQYRTICRGR

QSLQRLLDRDLENYISFYGHGPQIYVHSKIMIVDDRTVLIGSANINLWAEHLGIRQAEID

LVKDGVWMSYFFPLES

>SmaPLD13

YLNYFLILVNTREVCPKLREGLKADPVFDVLPMPKEKRAVRMWTSWAAGAHWVCYPHRFQ

SFAPERGRTHIDGKAAFDAMATAIEDAETEIFITGWWLSPELYLRRPFKRLDHLLQAKAK

MGIKIYILLYKEVSMLKKQRLLALHENIKVLRWPNSGVYLWSHHEKLVIVDNCFLGGLDM

CFGRYDDPTHPVCDSPSTIWGRDYYNRERNKLPRMPWHDVQCAIWGPPCRDIARHFVQRW

QSKAMVIPQAQCFD-QVIRSVSHWSATSQERSIEAAYCELIEKAEYFIYIENQFFIGLDG

DETIQNRVSQALYARIVRAYNEGLVFRVIVVIPLLPAGLDEVGAVRFIMHWQYRTICHGK

HSLEKLKETRVEDYISFYGHGPLVYVHSKLMIVDDRFVLTGSANINLWGEHLGLQHSELD

CIKDDIWMSYNFPLET

>SmaPLD14

MQLLLLLVCLSAAKQ------VQSIPVPGVLRGDQGNKSLDITSYY-----WE-------

---PKNPASG--------DYGYSQQQLDQFGAPVGQAVY-------------DSLKAAAD

RGVP-----------VQDTGFSPDFDS----------------HAKLWISNKAYVGSA--

---NND------------------------------WKSLTQVKEGIYFKRLEHFFENYW

--NATVDD--TEWQIKLPAYVSTPRVLNTYQTDEQGWVDTILSVPVNVRINTEYT-NTI-

---------------LSSAIAKVKHATVKVIVAHWAY-------TEQYL-----------

---------LFLNYTNALCSSPTRVNHAKYV-VSDVRANIGTSNL-LWDYYNGFGVKQLQ

AVFESDWNSYAVPLNA

>SmaPLD2

YATVDLAVGRTRVLKPVWDESVGKVPAAELLSGANGDSRIRFSLQFFAASDWGCNSHMAD

GFLPTIYQTGRQPTHCWEDVFKAITDACYLIYVIGWSVTTSITLVRDPQTLGELLKKKAD

EGVRVNLLVWDDKTSIHKDGVMGTNDQFLCPRNPNEIGTMFTHHQKLLVVDASFVGGIDL

CLGRYDTQHHPLFSTLNTVHSKDFHQNFKYGGPREPWHDIHSRLEGPVAWDVLFNFEQRW

KKQALLLSIPDTWNVQFFRSIDAGAAAAKDHSIHDAYINAIRRAKDFIYIENQYFMGSSQ

DAGALHLIPAELALKIVSKIEAGERFSVYIVVPMWPDGVPESGTMQAMLHWQNRTMQMMY

KQIEALRIHSPKDYLSFFCMELMIYVHAKMMIVDDEYIIIGSANINLWYEHTGFLIRLVN

SIGEKHWGIFTYPVLS

>SmaPLD3

YAYVVIAVASTRVIIPRWNEHVGRIPAEEVLKKQSGKAELRLKIVLHPIDRFMCRAHVFD

GELPDVILENFEHRRCWEEMCTAILEARYMVYIAGWSVYVKVRLIRDSSTLGELLRRKAA

EGVRVLVLAWDDKTSFKTEGLMGVHDEVLAPRYADVVGTLYTHHEKMLIVDASFVGGLDL

CDGRWDTPTHSLFANLKTFHKNDFHNTFDAGGPREPWHDLHCKIEGPAAYDVLTHFEQRW

RKDAELIKIPSTCHAQVFRSVDSGSMVERDVSIQMAYVKAIRSAQHFIYIENQYFIGSSK

NAGANHLIPMELALKVAAKIRDSQRFAVYVVIPMWPEGIPATAGLQEILFFQMQTMKMMY

GVIAALQDVHPRDYLNFYCREPMIYVHSKGMIVDDEYIICGSANINLCAEHLGLRVLRVN

EMAQANWEQYPYPISQ

>SmaPLD4

YAIVILAVARTRVINPKWNEHLGKIPIEQVLNGDKGEAKLHVSVHYLPVEKYTCKTHIYE

NTLPEVYLDGYHHKQCWEEMCTAIMEACHLVYIAGWSVYDKVKLIRDRNTLGELLKKKAG

EGVCVLLLIWNDKTSFKTDGVMKTHDEVLAARYGDVVGTLYSHHQKLMIVDSSFIGGLDL

CDGRWDTPLHHVFNSLQNEHKDDFYNSFEAGGPRQPWHDWHCKIEGPASYDILTNFEQCW

RKATELVNSGESWHVQVFRSIDGGSVVQTDVSIQMAYVKAIRMAQHFIYIENQYFLGSSD

VAGANHLIPMELTLKICSKIRAGQRFAVYIVVPMFPEGIPDSGPVQEILYFQSQTMKMMY

FLIDAIRDSHPQDYLNFFCREPMIYVHSKGMIVDDELVILGSANINLWAEHLGFLVHRVN

QMAEANWLQYVYPLNQ

>SmaPLD5

YVIVVLAVARTRVISPKWNEHLGKIPVEQLLGGDNGKAKLHLRVSYQPVEKYTCDTHIYD

STLPRIWLESYHHHRCWEEMCAAIAGAHHLVYLAGWSIYDKIKLVRDINTLGELLKKKSA

DGIRVLLLQWDDRTSLKTNGVMNTHDEILAPRYADVVGTLYSHHQKVVIVDTSFIGGLDL

TGGRWDTPSHLLFSSLQNEHKGDFRNSFEWGGPREPWHDWHCRIDGHAAYDVLTNFEQRW

RKATELLDIPETWHVQVFRSIDSGSVVQQDVSIQMAYIKAIRSAQHFIYIENQYFLGSSD

KAGANHLIPMELALKICSKIRAGQMFAVYVVVPMWPEGVPESAAVQEILYFQSQTMKMMY

KLIEALRDCHPRDYLNFYCREPMIYVHAKGMIVDDEFVIVGSANINLWAEHFGFEVRRVN

ALAEANWQQYPYPLNQ

>SmaPLD6

YATVDLAVGRTQVLKPVWNESLGKVPAEELLSESDDNSRIRFRLQFFEASHWGCKSHMAD

GFLPPIYQAGRQPTHCWEDVFEAISNARHLIYITGWSVYTQILLVQDPRTLGELLKKKAD

EGVRVNLLVWDDKTSLHETGLMATHDEYLCPRNPDTIGTMFTHHQKSIIVDASFVGGIDL

CDGRYDNQHHSLFRTLNDVHNKDFHQNFEKGGPREPWHDIHCRIEGPVAWDVLFNFEQRW

KKQALLIPIPDTWHVQLFRSIDGGAAAAKDRSIQDAYINAIRRAKDFIYIENQYFLGSSQ

DAGALHLIPMELTRKIVSKIEAGERFAVYVVVPMWPEGVPDSGSVQAILDWQRRTMEMMY

KEINALNAQSPKDYLTFFCREPMIYVHAKMMIVDDEYIIIGSANINLWYEHMGFLIRKVN

RIGDQLWDLFTYPVLS

>SmaPLD7

YASVVLAVASTRVISPHWNEHLGMIPAEEVLEKQSGKAKLHLKIIYHPIDSFKCRAHVFD

GELPDVILENFEHRCCWEEMCTAILKARHMVYIAGWSVYVKVRLIRDVSTLGDLLRRKAA

EGVRVLVLAWDDKTSFKTEGLMGVHDEVLAPRYADVVGTLYTHHQKVLIVDTSFVGGLDL

CDGRWDTPTHSPFATLQTTHKNDFHNSFDGGGPRQPWHDLHCKIEGPAAYDVLTNFEQRW

RKATELIQISSAWHAQVFRSIDSGSVIEQDVSIQMAYVKAIRSAQHFIYIENQYFIGSSK

NAGANHLIPMELALKVASKIRDHQPFAVYVVIPMWPEGVPDSGPMQEILFFQTQTMKMMY

GVIAALSDVHPCDYLNFYCREPMIYVHSKGIIVDDEYIICGSANINLWAEHLGFRVRRVN

ELAQANWEQYRYPINQ

>SmaPLD8

YATVDLAVGRTRVLKPVWNESLGKVPAADLLSGAEEQSQIRFSLQFSEASRWSCKSHMPD

GFLPRIYQTSRQPTRCWEDVFEAISNARFLIYITGWSVYTEITLVRDPRTLGELLKKKAN

EGVTVNLLVWDDRTSVHPAGIMGTHDEFLCPRNPDTIGTMFTHHQKTIMVDVSFVGGIDL

CDGRYDTQYHSLFRTLNGVHSNDFHQNFKHGGPREPWHDLHSRVEGPIAWDVLFNFEQRW

RKQALLLPIPDTWHVQFFRSIDAGAAAAKEHSIHDAYINAIRRARDFIYIENQYFVGSSQ

DAGALHLIPMELALKIVSKIEAGERFTVYVVLPMWPEGVPDSGSVQAILHWQRRTMEMMY

KQIEALRIHTPKDYLTFFCREPMIYVHAKMMIVDDEYIMIGSANINLWYEHMGFLIRKVN

SIAERHWDLFTYPVLS

>SmaPLD9

YASVVLAVARTRVISPTWNEHLGTIPVERVIDGQSGNAQCRFKLVFSPVQNFTCDAHIFD

GSLPEVMLEDFHQHRCWEELCTAILDAHHMVYIAGWSIYHQVKLLRDTSTLGELLKRKSA

EGVRVLVLAWDDKTSIKNEGLMGVHDEVLAPRYGDVVGTLYTHHQKLVIMDTSFIGGLDL

CDGRWDTPTHSLFATLQTLHKNDYHDTFEAGGPRQPWHDWHCKIEGPAAYDILTNFEQRW

KKATELIQISNTWHVQVFRSIDSGSAAEKDISIQMAYIKAIRSAQHFVYIENQYFIGSSV

NAGANQLIPMELALKIASKIKAGERFAVYVIIPMWPEGVPDSAAVQEILYFQAQTMKMMY

GVINALREMHPQEFLNFYCREPMIYVHAKGMVVDDEYVICGSANINLWAEHLGFTVQRVN

EMAQANWEQYPYPVAQ

>SmPLD1

YATIDLQVGRTRLIRPVWNESVSKVPVKRIMYGESGKSRVRLRLQFFDITRWGCKAHMTD

NFLPKIYLHGREPTRCWEDVYEAIANAQHFIYITGWSVYAEITLIRDSAKLGDLLKKKAD

QGVRVLMLVWDDRTSVKMEGLMATHDQLLCPRTPDQIGTMFTHHQKTIIVDASFVGGLDL

CDGRYDTQFHSLFRTLGTVHKEDFHQNYDKGGPREPWHDIHSKLEGPVAWDVLRNFEQRW

HKQGHLVS-GNTWNVQLFRSIDGGAAAAADRSIQDAYIHAIRRAKDYIYIENQYFLGSSH

DAEAHHVIPRELALKIVSKIEAGERFSVYVVLPMWPEGVPESGQVLAILDWQKKTMQMMY

KAIQALDAQSPRDYLAFYCREPMIYVHSKMMIVDDEYIIVGSANINLWYEHMGFLMRKVN

RTADQLWHMYAYPVKS

>SmPLD2

YATVDLTVGRTRVVDPVWNESIGKVSVLNLLSGQDGKAKVRVRLQFFDVTKFGCRAHMSD

NFLPPIFLAGYQPTRCWEDMFHAISNAKHLIYITGWSVFTEITMIRDPATLGQLLRRKAE

EGVRVLLLVWDDRTSVKTEGVMNTHDERLCPRNPDQIGAMFTHHQKTLTVDVSFVGGLDL

AAGRYDNQHHSVFHTLNAEHKDDFRQSFKFGGPREPWHDIHARIEGPAAWDVLHNFEQRW

RKQAWLLEMRNTWNVQLFRSIDAAAVAGADRSVHDAFIAAIRRAKDFIYIENQYFLGSSQ

NAGAHHLVPREIALKIVSKIQAGQPFRVYIVIPMWPEGVPDSPQVMAILEMMVRTMEMMY

RTIPALFAKDLRDYLSFYCREPMVYVHSKMMIVDDEYIIVGSANINLWYEHTGFLMRKMN

SMGDYYWQLYAYPIRS

>SmPLD3

YVVLSIALGRTRI--PSWNEQFGRIPAEEVLSKKRGRTQLNLFIKYTPVEEYICRAHAEV

PPLKPISLDNFVRQCCWEDLCRAIVDAHHIVYIIGWAVFHRTRLVRTGHTLGELLKQKSA

EGVRVLLLVWDDKTSFKIDGVMGTHDEVLSPRYGDIVGTLYTHHQKLVIADTSFLGGLDL

CDGRYDTQKHSLFNTLTTIHKDDCYNMFSSGGPRQPWHDQHCMLEGPAAYDCLKNFEQRW

LKSSELVQISETWHAQVFRSIDSGSVAEVDKSIHAAYVERIRSARHFIYIENQYFLGSSK

NAGATHLIPMEIALKIDANIRKRKRFAVYVLVPMWPEGAPDSASGQEILYFQAQTMETMY

RVVKALADTHPRDYLNFYCREPLIYVHAKSMIVDDEYVIIGSANINLWAEHLAFEVRRVN

HLAEENWKQYPYPVPH

>SmPLD4

YATVDLTVGRTRMIG--WNESIGSIEVSELLSGENGESKVRIKLQFHEATNFGCKAHMTE

NFLPDIKLAGHAETRCWEDVFEALTNAKHLIYITGWSVYTEIKLIRDPETLGELLKRKAE

EGVRVLMLVWDDRTSVRMDGLMATHDQLLCPRNPDQIGTMFTHHQKTIIVDASFVGGIDL

CDGRYDNQYHSLFRTLDSYHGDDFHQNFKKGGPREPWHDIHSKLEGPVAWDVLHNFEQRW

LKQALLLPVSSTWNVQLFRSIDGGAAAAKDRSIQDAYIHAIRRAKDFIYIENQYFLGSST

DAGAYHLIARELALKVVNKIEQGERFVIYVVVPMWPEGLPESGQVLAILDWQKKTLDMMY

RSIQALDAKSPKDYLTFFCREIMIYVHSKMMIVDDEYIIVGSANINLWYEHLGYLVRKVN

ELADEYWAMFSYPIKS

>SmPLD5

YVVFSLALAKTKVISPHWGERFGKIPAHRIASGPGGKAQLKISIKYTPVEQYQCSAHCPD

GGLPEITLEGYEHGKCWEDICQAILEAHHLVYIAGWSVFHKVKIVREPETLGELLKRKAA

EGVRVLLLVWDDKTSFTTEGVMGTYDEVLSPRYGDVVGTFYTHHQKLVIVDSSFIGGLDL

AQGRYDTPEHPLFKTLGSIHRDDYHNTFDHGGPRQPWHDLHCRIDGPAAYDVLTNFAQRW

RKAAAMIEISETWNVQVFRSIDSGSVCQKDTSIHTAYVERIRSAQHFIYIENQYFLGSSK

KGGATHMIPMELALKVASKIRSGDPFAVYVVIPMWPEGVPDSATVQEILYFQSQTMKMMY

KIIQALNEVHPTDYLNFYCREPMIYIHSKGMIVDDEYVIIGSANINLWAEHLGFNVHKVN

EIAQGNWKQFSYPMHS

>SmPLD6

YLDHFVLLLNSEAVCSKLKEGLKAHPVFDVLS--PERRSVRVRTKGARGAAWICHPHRFG

SFAPPRGLTSIDGKSAFDAIASTISSAKSEIFIADWWFCPELYLRRPFARLDSLLGAKAR

EGVQIYVLLYKEVALLKKRKLLGIHENIKVLRYPDSGVYLWSHHEKIVIVDQCFLGGLDL

CFGRYDDPHHLLRDDQAVIWGKDYYNRERQKTPRMPWHDIQCAIWGPACRDVARHFVQRW

RNKAMVIPASDWWE-QVVRSVGQWSATSQERSIEAAYCSLIDKAEHFIYIENQFFIGLDG

DSVIQNRVLESLYTRILRAYKEKQCFRVIILLPLLPGGVDDTGAVRAIIHWQYRTICRGN

SSLKRLRDTAVEDFVSFYGHGPQIYVHSKILIVDDRVTLIGSANINLWVEHLGFQASEVN

VIKDDVWTSYCFPLES

>C.subellipsoidea

------SRARTSIKEPVWNEVIGRVAVAELVKGQH--AALRATVSFRSVFAFRCRAHQEL

DT-PPIQLSDFQPRGAWTDLYEALCSAQHLIYIAGWSVYDKITLIRDPSTLGELLIKKAE

EGVRVLLLVWDDQTS--RNGMMATHDEALVPREGGTKGNMFTHHQKSVILSTAFMGGLDL

CDGRYDTPQHTLFHTLNTVHSEDYHQCCKFGGPRQPWHDIHCRLQGPVATDVLVNHVQRW

LKQAKLLPMPEAWRAQLFRSIDSDSASA-DASIHSAYVTAIRRAQRFIYIENQYFLGSSR

SAPCRHLIPLEIALKVVSKINAGERFAVYIVLPMYSEGDPESGSVQEILRFQTHTMRMMY

TLIGALRAKHPRDYLNFFCREPLIYVHSKMMIVDDEYIIVGSANINLWKEHTGFYAREMH

GIGKANWDAFLYPITS

>P.yezoensis_contig_17468_g4270

YVTVSVRLVKTATVDPVWNTTVGAIPVARLMA--DGSGWITVSVTYTPCSY--GVAHVHA

GTLPPVVNGHVTGRTAWVDLWAALDAARVVIYIAGWAVDANLQLVREAPTLGELLKRKAA

AGVRVAIMVWNELASAGSDGLMGTGDEVGVSRRDDAVGGLWTHHQKTVVVDSAFLGGLDL

TTGRWDSPQHSLFRTKTTYHAADFHQCLPAYSPREPWHDIHASVSGAPAVDVMANFCARW

ATQVAVLPFDEEWAVQILRSIDGRSTASRDQSIYKGYIWAIRGARRFVYIENQYFMGSSP

SGAVDQLVPMELALKIAHKVRARESFSAYVVIPLQPEGTQAAA-VEEILFWQGQTVRMMY

ARIAVLDEVLPTDYLQFFAREPAIYVHSKMAIFDDDYLIVGSANLNLWAEHTGFLARRVR

AIAEANWAAFPYPYDS

>P.yezoensis_contig_1916_g311

YVTVRLKIVKTATIQPEWDLDLSKLDAEELASGKKNRGELRLLIKFTPLAD--CRAHTPK

GSLPSAFEGQPETVSAWTDIYQSISDAKHVIYIMGWSVWVDVVMVRDGDPLGQLLLKKAE

EGVRVNLLVWDEVLSFHSTGLMDTKDEAIVPRVADSVSGLFTHHSKAVVVDSAYVGGLDL

TYGRYDTPSHSLFTTLRTTHAEDFHNCFKEKGPREPWHDIHCRLDGGIARDVAEYIEERW

RGQAALVDAANTWSAQMFRSCDERSAVERDKSIQRAYVHHIRRAQRYIYIENQYFLGSSR

HKDAQHLVPLELVLKIEDKIRRKERFAVYVTIPLWSEGPAADKATQEVIFWQYRTVEMMY

SRVEALKEAKATDYLNFFCREPQIYVHAKMMIVDDEVIVLGSANINLWIEHTSFHVQRMR

ELGEEGWQEFLYPYDS

>P.yezoensis_contig_21850_g5396

YVSVALNYFQSRVVNPVWDSSLGELPADELVSSGDDDGSVTVKVRLLPLDSYACQAHVDE

NELPEVP---YIPGCCFRDMYAAIDAAQDFVYITGWSVFTEISLLRERHTLGALLKRKAE

EGVQVRIMVWDELMSLGHKGMMMTHDEVKAARKNDGSTAMFTHHQKTIICDASFVGGLDL

TNGRFDTPTHSLFRTLASTHPPDFHQCVPTKGPREPWHDQAGHVSGAASWDVLQNFTERW

HRQAALADREGAWNVQILRSINEASAVDSDQSIHHAYVHHVRRSKRFCFFENQYWLGSSR

NESADHLLPVEIAMRVARAVKEQEPYHAYVMIPLFPEGPPASGSVQEILAFQFQTFAMMY

KIINAIREA-PIDRLSFYFREASVYCHSKAVIFDDAITIIGSANCN--------------

----------------

>P.yezoensis_contig_8068_g1891

FAELSF---NSRVLAPLLERF--PAPVSSAASGSNSKAALRLRRQIRPRSQLARSEVLTE

TLLPALRGHG-------------------------------IELIP---SMTAYFRRSLS

HGIYLLVVLLNPESG------------RLLPVTP------VTRNTKLVVDDT--------

---LFGESDNLL----------DYV-----------WEEVHRRRSAPATADVARLAAELY

----PLLGCM-----RIFSVIRKGDV---AHSFTGSALLFLRAAARDVRVIKQVLYGGSS

P----------LVASLIRAAKSGKEVTVLVELKASFDRLQRAGCSYGLVGLKCKTMVVVR

EEEGGLRAYLYTDMALFTC--PLV---APVSMLDRVVAMIAAEAAHSWVGHRGFIKRKLR

KSLNHTWQLYARP-AA

>P.umbilicalis_13079

YVSVALNYFQSRVINPVWESFIGELPADELVQSGDDDGSVTVKMRLLPLDAFACQAHVDD

GELPAVD---YTPGCCFRDMSNVMEAAQDFIYITGWSVFTEISLLRERHTLGAFLKRKAE

EGVQVRIMVWDELMSLGHKGMMMTHDEVKAARKNDGSTAMFTHHQKTIICDASFVGGLDL

TNGRFDTPTHSLFRSLATTHPPDFHQCVPTKGPREPWHDQAGHVSGAASWDVLQNFTERW

RRQAALVDRRDAWNVQILRSINEASAEDNDQSIHHAYIHHVRRSKKFLFFENQYWLGSSK

NESADHLLPVEIAMRVARAITAHEPYHAYIMIPLFPEGPPASGSVQEILAFQYQTFAMMY

RVVQAICGA-PLDRLSFYFREASVYCHSKAVIIDDAITIIGSANCNLFGEHLNFEVRKVR

EIAEANWEAYPYPIDG

>P.umbilicalis_395

-----------------------------------------------------GTAHVHA

GMLPPVVDGNTVGRPAWVDLWAALEGASIVIYIAGWAVDAGLRLVREATTLGELLKRKAA

SXVRVCVMVWNELASVGSGGLMGTGDEVGVSRRDDAVGGLWTHHQKTVIVDSAFVGGLDL

TTGRWDTPQHSLFRTKTTYHSADFHQCLPAYSPREPWHDIHASVSGAPAVDVMANFTARW

AVQAAVAPFADEWGVQILRSIDGRSAAARDDSIYRGYLWAIRGAQRFVYLENQYFMGSSP

SDAVDQLIPMELTLKIIHKIRARCPFAVYVVIPLQPEGTSPAA-VEEILFWQGQTVRAMY

TRIAALDEVLPTDYLQFFCREPAIYVHSKMAIIDDEYLIVGSANLNLWAEHAGFLARRLR

AIAEANWVDYPYPYDS

>P.umbilicalis_7704

YVTVRLKIVKTATIQPDWGLDLSKLDAEELAAGKKNRGQLRLFIKFTKLAD--CKAHTPQ

GSLPSAFESQPETVSAWTDIYEAMMGAKHVIYIMGWSVWVDVVMVRDGDPLGQLLLKKAE

EGVRVNLLVWDEVLSFHSTGLMDTKDEAIVPRVADSVSGLFTHHSKAVVVDSAFVGGLDL

TYGRYDTPSHSLFGTLRGVHSEDFHNCFKEYGPREPWHDIHCRLDGGIARDVAEYIEERW

RGQAALVDAANTWSAQMFRSCDERSAVERDKSIQRAYVHHIRRAQRYVYIENQYFLGSSR

HKDAQHLVPLELVLKIEDKIARKERFAVYVTIPLWSEGPAADKATQEVIFWQYRTIEMMF

ARVDALKEAKATDYLNFFCREPQIYVHAKMMIVDDEVIVLGSANINLWVEHAAFNVHKMR

ALGEKGWQEFLYPYDS

>P.purpureum_2164

YVKVKVRILKSHVVAAAFFQDLALIPAGSLTEGNDAEAKVKLLDKMKAITV--CMAHEMS

YLQP------LRRRALWEELYASLHAAEHLILIAGWSVWHDLRLLRERPTLGELLHERAE

RGCTVRVLVWDDPSS--VQGMMGNHDELKQARIPALASSMFTHHQKIVVCDAAFVGGLDL

CDGRWDTPEHFLFKHLNTYWKDDFHNNFPEYGPREPWHDVHSLIEGHAAVDVLRIFEARW

----NLVC--NAWSVQMVRSIDERSSMKKDTSVLSAYVHYIRRAKRFVYIENQYFMGSSK

ENLAPNTIPMEIALRICRAIREREPFCAFIIIPLWPEGVPESMPVQEILFWQYQTVEMMY

RVIQELQEVRPEDYLQILTRE-PIYVHSKLMIVDDAFLIIGSANINLFAEHMG--AREIA

ALADHCWKFYRYPYDT

>P.purpureum_3427

FGELSF---NQRVLAPLLERF---AP-----------DTSKMKASIRPRTEFSTSKCLMR

DILPKLQEHG-------------------------------VKILR---DMDEYFRKKLS

HGIYLVIVVLNPETS------------RVLPVGSD-----VTRNVKLVIDDT--------

---LFGESDNLL----------DFV-----------WEEVHRRRSAPATADIRSLLVREY

----NLLGFF-----KIFSVIRKEDL---TMSFNESSLLFLRAAARCVRMIKCVLYGGSS

P----------LVASLIRAAKNGKEVSVLVELKASFDQLMDAGCSYGLVGLKSKIMLIVR

QEENGFRQYIYTDLALFTCRE-LV---APVNMLDSFIELIDNEAENSWIGHRGFIQRKLM

KMMTNTWEMYANPVES

>P.purpureum_4459.4

YFTCRIHCIKSHAVVPTWEVTSGFIPTVDLIASRKGKGELNLAVVFEPLEA--NKAHHKD

GELPKIVKANYRHGKCWEETYQAILDAKYFVYACGWAIYSDISLIRDPPTVGELFLKKAS

EGCRVLLMPWDEMIS--SAGLMGTHDEEPVLRKDDAVGGMWTHHQKSIIVDAAFLGGIDI

TDGRYDYPEHPLFRTLATCHGNDFYQCWNKYGPREPWHDIHMKVEGNAAHDVLKNFEDRW

KKQVKLYK--KNYDEQFFRSIDERSA---DASIHRAYVHQIRRAKKFLYIENQYFIGSSQ

YDFALHIIPLEIVQKIIEKT--------------------------------RRT-----

--------------------------------------------------HT-F------

----------------

>K.nitens_GAQ78336.1

YAAVDFAVARTRVIKPRWNERAGKIPAEVLLKERNGNAKIKLGFKYTPMDEYKGRAHVKD

GLLPQIELDGYQPGRCWEDICKAIVDAKYMVYIVGWSVFTDITLVRDPDKLGDLLKWKAK

QKCRVLLMVWDDRTSVKITGVMDTHDEKLVPRYPDMVGSMYTHHQKTVIVDTAFLGGIDL

CNHRYDDQHHSLFRTMNGISKGDFENNIKTGGPREPWHDIHCKVEGAAAWDVYTNFLQRW

HKVRGLLKMKENWHVQVFRSIDSGSVVFQDMSIQDAYVHAIRRAQRFIYIENQYFLGSCQ

EVGAWNLIPVEIALKIVGKIREGKQFRAYVLVPMWPEGDPTSGSVQEILFWQARTMEMMY

SLIKALKEQHPRDYLTFFCREPMIYVHSKLMIVDDEYVIVGSANINLWAEHLGYKVRKVN

AAVDANWQVFAFPVTS

>K.nitens_GAQ88090.1

YVNHFLLLVNTREVCPKLHEGLKADPLFDVLQAEKYKRVLQIRTRHGAGSKWVCHPHRFG

SFAPPRGMIAVDGRAAFEAVANALESAKNEIYITGWWLCPDLFLRRPFATVAAILEERAR

AGVQVYILMYKEVSILKKKKLQALHENIKVVRHPDAGVYLWSHHEKIVVVDHAFLGGLDL

CFGRYDTPEHSLGDLPATVWGKDYYNRERSRTPRMPWHDVHCALWGPPCRDVARHFVQRW

RSKAMVIPAAERLEQQIVRSVGQWSATTQERSIHEAYCTLITNAQHFVYIENQFFVGLDE

DDTIQNRVLQALFARILRAHRERATFRVIVVMPLLPGGVDDAGAVRAIMHWQYRTICRGK

HSLTRLAEEAVSEYVGFFAHGHQVYVHSKLMLVDDRVALIGSANLNLWAEHLGLKEADMD

GIRDGLWMAYTFPLES

>R.subcapitata_GBF94843.1

YAVLELTRLRTSVTSPEWRERVGFVDASELLDARNGSARIHFSIRYSSVET--NRAHQNP

GPVRDILLGNFCESSCWDDVYHALTSARRFILITGWSVWVHTLLKRGPAPLGQLLLKKAG

EGVKVLMLVWDDASNLGHPGLMATHDNVLCPRQGG-----FTHHQKTIIVDASFVGGLDL

CDGRYDTCEHPLFGTDGPPHEGDHHQNVERGGPREPWHDIHARIEGPAAYDVMVNFIERW

SKQAHALKFGDSWGVQVFRSIDSDSATYEDRSIQAAYVQAIRRAKRYIYIENQYFLGSSK

DAAAVHLVPAELALKLEAKIRAREPFRVYVVLPLWPEGVPTSGSVQDILAFQARTMAMMY

RRVAAIRDSYPTDYLQFFCRQPMIYVHSKLMIVDDEYAIIGSANINLLKEHIG--ARRLR

AIGDANWVAWTYPVRG

>C.braunii_GBG68949.1

YVSRFLLVVNTREVCPKLHEGLKADPLFDVLAGREQRRTIPIRTKRASSARWVGHPHRFG

SFAPPRGMTKIDGRSAFEAIAEALLAARSVVYITGWWVCPDLYLRRPYERLDRILEGKAK

QGVQIYVLLYKELALLKKRKLMSLHENIRVVRYPDAGVFLWSHHEKIVVVDHCFLGGLDL

CFGRYDTQDHVLSDHPASIWGKDYYNRVRQIVPRMPWHDVHCALWGPPCRDVARHFVQRW

RSKAMVIP--EWWESQVIRSVGHWSATSQERSLHEAYCSLIEKAEQLIYIENQFFIGLDG

DDVIQNRVLQAIFARVLRAHREGRHFRVIVVLPLIPGGVDDSGAVRAIMHWQYRTICRGK

NSLSNLAKEAAEDYVSFYGYGAQIYVHSKIMIVDDRTVLIGSANINLWAEHLGINKNERH

LIKDHLWRAYCFPFES

>C.braunii_GBG72654.1

YATVELAVARTCVIQPAWNEKVGRIPVDTLLTEEGDEASIRLKVKFNSIDT--NHSHIPN

SFAPYIELEGYKQQRLWEDIYKTFVEAKHFIYICGWSVYYKIQLIRDEETLGDLLVRKAN

EGVKVLLLIWDDRTSIGSSGIMGTHDQELCPRDPDQIGMMFTHHQKLVVADASYVGGIDL

CDGRYDTPNHQLFRTLDTWHKEDMHQNFEQGGPREPWHDIHAKVEGPTAWDVLYNFEQRW

RSQRELYDNQEAWNVQFFRSIDTGSVAGEERSIQDAYVYSIRRAEQFIYIENQYFLGSCR

TVSAIHTIPVELALKIVRKIDANEPFAVYVVIPMWPEGIPTDGSVQEILKWQHRTMQMMY

KLIEAIARNKPTDYLNFYCREVMIYVHSKMMIVDDEYIIVGSANINLWAEHTGFLVLRMN

EIAHENWAQFPYPVKK

>C.braunii_GBG85073.1

YVVVDIAVARTCVINPQWNETIGRVPVEALAVANDGSCLVDVSCHYKPAQSWYCRAHVAD

GALESIRLDGYTHGRCWEDICAAIQDAKFMIYIIGWSVYDKIKLIRDPRTLGELLKKKAG

ANVRVLLMIWDDNTSMK-QGVMNVHDEVLAKRTADSTAIQFTHHQKAVLVDVAFIGGIDL

CNGRYDNQSHPLFHTLHVTHGEDYYQCIAHGGPRQPWHDIHCRLEGPAAYDVLKNFTERW

EKSASLLEIQDSWHVQVFRSIDSGSVVRAERSVQDAYINAIRSAQHFVYIENQYFMGSSR

SAGCDNLIPAEIALKIVDKIRQNERFAAYIVIPMFPEGAPDSGVGQTLLYWQYQTIEMMY

KLISALIETHPQEYLNFFCREPMIYVHSKMMIVDDEYVIVGSANINLWAEHLGFMVNRVR

GAAAANWQDYPYPITA

>C.braunii_GBG88554.1

------------------------------------------------------------

------------------------------------------------------------

------------------------------------------------------------

------------------------------------------------------------

------------------------------------------------------------

---------MEIALKIVKKIKAGERFAAYILIPLFPEGDPAAAAGQEILYWQNLTIKMMY

KMIDTLQDVHPRDYLNFYCREPMIYVHSKMMIVDDEYIVVGSANINLWAEHLAFEVHKVN

MMAESNWNLYTYPIPQ

>C.primus_QDZ18356.1

VASVVLVISLV-VMY------VESIPYSGAMSYKMDNESIDLTALY-----WT-------

---ARNAMKNYYSQNCPDDAHFNQTQLEEFGVYQGHEVY-------------EAIKRAAS

RGIK-----------VQSPGLGNGFE-----------------HAKVWVVDGGYIGSA--

---NMD------------------------------WRSLTQTREGVFIRDISNIFESFW

EGQANIID--PHLQIKFSMFETQ---VKMRISDGNMLVETIESASVGVYVSVRFLCYEQ-

---------------LLHALLGAKGADVRLLVSRWWY-------AQDVLR----------

---------KYLEYSDIICASPSRVSHSKFI-VSDNLVNIGTSNM-EWSYHSGFNINQVK

NMFEVAWDHYTHPI--

>AtPLDFF1

YLNHFLLIVNSREVCPKLKEDLKEDPVFDVLPSNKDHRSIRIRAKNSAKVKWVCHPHRFG

SYAPPRGLTDVDGGAAFAAIAAAIENAKSEIFICGWWVCPELYLRRPFDRLDNLLENKAK

QGVQIYILIYKEVALLKKRRLLGIHENVRVLRYPDSGVYLWSHHEKLVIVDNCFIGGLDL

CFGRYDTFEHKVGDNPSVTWGKDYYNRERKKHPRMPWHDVHCALWGPPCRDVARHFVQRW

RNKAMVIPDDEWWETQIIRSVSQWSATSQEESIHSAYRSLIDKAEHFIYIENQFFIGLSG

DDTVKNRVLEALYKRILRAHNEKKIFRVVVVIPLLPGGIDDSGAVRAIMHWQYRTIYRGH

NSITNLYNTKAHDYISFYGYGPQVYVHSKIMIVDDRAALIGSANINLWSEHLGLRTGEID

QIIDEIWMAYSFPLES

>AtPLDFF2

YLSLFLLIVNSKEVCSKMKEGLKEDPVFDTLGKEKEHRTVRLRTTSSRKVKWVCYPHRFG

SFAPPRGLTSVDGHTAFEAIAFAIQNATSEIFMTGWWLCPELYLKRPFERLDALLETKAK

QGVKIYILLYKEVQILKKKRLQNIHKNVKVLRYPDSGIYLWSHHEKIVIVDYCFIGGLDL

CFGRYDTAEHKIGDCPPYIWGKDYYNRERRKYPRMPWHDVHCALWGPPCRDVARHFVQRW

RNKAMVLPHDDWW--QIIRSVSQWSATSQD-SIHRAYCSLIQNAEHFIYIENQFFIGLEK

EDTILNRVLEALYRRILKAHEENKCFRVVIVIPLLPGGIDDFGAVRALMHWQYRTISREG

TSIDNLNALKTQDYISFYGYGPQIYVHSKLMIVDDRIAVIGSSNINLWSEHLGLHAGEIQ

KIEDDLWMAYCFPLES

>AtPLDAA1

YATIDLAVGRTRKIKPKWYESIGYIPVDQVINGEDRNSKIHVKLQYFHVEEWNCKAHIPD

NFVPRIPLAGYEPQRCWEDIFDAISNAKHLIYITGWSVYAEIALVRDSRTIGELLKKKAS

EGVRVLLLVWDDRTSLKKDGLMATHDEILCPRNPDQISTMFTHHQKIVVVDSSFVGGIDL

CDGRYDTPFHSLFRTLDTVHHDDFHQNFTKGGPREPWHDIHSRLEGPIAWDVMYNFEQRW

SKQGILVKLSDVWNVQLFRSIDGGAAAAEDRSIQDAYIHAIRRAKDFIYVENQYFLGSSE

DINALHLIPKELSLKIVSKIEKGEKFRVYVVVPMWPEGLPESGSVQAILDWQRRTMEMMY

KDVQALRAQDPRNYLTFFCREPMIYVHTKMMIVDDEYIIIGSANINLWYEHLGFLIEKVN

RISDKYWDFYRYPIKS

>AtPLDAA2

YATIDLSVARTMMRRPKWLQSVSYLPVTEVITGQNRRSKLHVRVKFTHVTQWNCKAHVLN

EY-PDVTLTGYKHHRCWEEIFDAIWEAKHLIYIAGWSVNTDVTLVRDPKKLGELLKKKAE

ENVTVLMLVWDDRTSFKRDGLMMTHDQVLCPRNPDEVATMFTHHQKTIVVDSSFLGGIDL

CDGRYDTVEHPLFGTLNSVHANDFHQNFKKGGPREPWHDIHCKLDGPAAWDVLYNFEQRW

MKQGYLISLAEGWTVQVFRSIDDGAVAASERSIQDAYVNAIRRAKNFIYIENQYFLGSSN

EINALQLIPKEISLKIVSKIEAGERFSVYIVIPLWPEGKPGSASVQAILDWQRRTMEMMY

TDIIALRKKNPRDYLTFFCREPMIYVHSKMMIVDDEYIIIGSANINLWLEHLRFQIRMVN

ATADELWGLYSYPIKS

>AtPLDAA3

YATIDLAVGRTRKITPKWFESIGYIPVEDILHGEEKNSKIHVKLQYFGVEKWNCKAHIPG

NFVPKIPLAGYEPHRCWEDIFDAITNAKHLIYITGWSVYTEISLVRDSRTVGELLKKKAS

EGVKVILLVWDDRTSLKKDGLMATHDEILCPRNPDQISTMFTHHQKIVVVDSSFVGGLDL

CDGRYDTPFHSLFRTLDTAHHDDFHQNFTKGGPREPWHDIHCRLEGPIAWDVLYNFEQRW

SRQGILVKLGDVWNVQLFRSIDGGAAAAEDRSIQDAYIHAIRRAKDFIYIENQYFLGSSE

EINALHLIPKELSLKIVSKIKAGEKFKVYVVVPMWPEGIPESGSVQAILDWQKRTMEMMY

KDVKALRENDPRDYLTFFCREPMIYVHTKMMIVDDEYIIIGSANINLWYEHLGFLIQKVN

RVADKYWDLYRYPIKS

>AtPLDAA4

YVTIKIKVAKTS---RIWNQTLKRISAEQILTSNNGSLKLKCLMWFRPAYLWCCRAHHKA

TFDPRVDDVPFNARNLWEDVYKAIESARHLVYIAGWALNPNLVLVRDNETVGELLKRKSE

EGVAVRVMLWNDETSIKNKGVMRTNVERLCPRLHKKLPTAFAHHQKTITLDTSFLGGFDL

CDGRYDTEEHSLFRTLGT--EADFYQSVSRGGPREPWHDCHVSVVGGAAWDVLKNFEQRW

TKQCVLVNIRRKWNVQVLRSIDHISA---EKSVHDGYVAAIRKAERFIYIENQYFMGSCK

NSGCTNLIPVEIALKIAAKIRARERFAVYIVIPMWPEGPPESETVEEILHWTRETMSMMY

QIIEAIWEVHPRDYLNFFCREPMVYVHSKLMIVDDTYILIGSANINLWYEHTG--VRGLR

TIGEQMWEIYAYPIRS

>AtPLDBB1

YVSVSVAIGRTYVMSPVWMQHVGTIPVEQIYSGANGKANLSLSIQYTPMDKYHGTAHVPE

GMLPGIRLDNYEHGKCWHDMFDAIRQARRLIYITGWSVWHKVKLIRDKLTLGELLRSKSQ

EGVRVLLLIWDDPTSYKTDGVMATHDELLCPRNAGEVGTIYTHHQKNVIVDAAFVGGLDL

CDGRYDTPQHPLFRTLQTIHKDDFHNTFLSGCPREPWHDLHSKIDGPAAYDVLTNFEERW

LKAAALLRIPEAWHVQIFRSIDSNSVATCDMSIHTAYVKAIRAAQHFIYIENQYFIGSSK

DIGANNLIPMEIALKIAEKIRANERFAAYIVIPMWPEGVPTGAATQRILYWQHKTIQMMY

ETIKALVETSPQDYLNFFCREPMVYVHSKGMVVDDEYVVIGSANINLWAEHMAFTVRKVR

TMGERNWKQFKYPVFI

>AtPLDBB2

YVSISVAIGRTYVISPVWQQHVGTIPVEQIYSGANGKATLSLSIQYTSMNKYHGSAHVPE

GMLPGIKLGNYEHGKCWHDMFHAICQARRLIYITGWSVWHNVRLVRDKERLGELLRSKSQ

EGVRVLLLVWDDPTSYMTDGVMGTHDELLCPRNAGEVGTIYTHHQKNLIVDAAFVGGLDL

CDGRYDTPQHPLFRTLQTDHNGDYHNTFVSGCPREPWHDLHSKIDGPAAYDVLTNFEERW

LKAAALLRIPEAWHVQIFRSIDSNSVATSDMSIHTAYVKAIRAAQHFIYIENQYFIGSSK

DIGANNLIPMEIALKIADKIRAKERFAAYIVIPMWPEGVPTGAATQRILYWQHKTMQMMY

GTINALVEASPQDYLNFFCREPMIYVHSKGMVVDDEYVVIGSANINLWAEHMAFVVRKVR

TVAEENWEQFKYPVFL

>AtPLDRR1

YVTVSIAIGRTFVISPVWMQHIGGIPTEQLCSGNSGKAVLGLSIQYTPMERYQGRAHVDD

GTLPSVHLDGYRHGKCWEDMADAIRQARRLIYITGWSVFHPVRLVRRTNTLGELLKVKSQ

EGVRVLVLVWDDPTSFKTQGVMNTSDELLCPRSGGEVGTIYTHHQKTVIVDAAFVGGLDL

CNGRFDTPKHPLFRTLKTLHKDDFHNNFADDGPREPWHDLHSKIDGPAAYDVLANFEERW

MKASSLLRIPESWHVQVFRSIDSSSVATGDMSIHAAYVKAIRSAQHFIYIENQYFLGSSK

DLGANNLIPMEIALKIANKIRAREKFAAYIVIPMWPEGAPTSNPIQRILYWQHKTMQMMY

QTIKALVEVEPQDFLNFFCREPMIYVHSKGMVVDDEFVLIGSANINLWAEHLGFEVRRVR

QLSELNWRQYKYPVFL

>AtPLDRR2

YVTVSIAIGRTFVISPVWMQHIGGIPTKQLCSGNSGKAMLSLSIQYTPMERYQGRAHVDD

GTLPSVHLDGYRHGKCWEDMADAIRRARRLIYITGWSVFHPVRLVRRNNTLGELLKVKSQ

EGVRVLVLVWDDPTSFSTKGLMNTSDELLCPRYGGEVETIYTHHQKTMIVDAAFVGGLDL

CNGRFDTPKHSLFGTLKTLHKDDFHNNFEDVGPREPWHDLHSKIDGPAAYDVLANFEERW

M-ASSLLRIPESWHVQVFRSIDSTSVATGDMSIHAAYVKAIRSAQHFIYIENQYFLGSSK

DLGANNLIPMEIALKIANKIRARENFAAYIVIPMWPEGAPTSKPIQRILYWQHKTMQMMY

QTIKALLEVEPQDFLNFFCREPMIYVHSKGMVVDDEFVLIGSANINLWAEHLGFEVRRVR

QLSELNWGQYKYPVFL

>AtPLDRR3

YVTVSIAIGRTFVISPVWMQHIGEIPTEQLCSGNRGKAVLSLSIQYIPMERYQGRAHVDD

GTLPSVHLDGYRHGKCWEDMADAIRRARRLIYITGWSVFHPVRLVRRNNTLGELLKVKSQ

EGVRVLVLVWDDPTSFSTKGLMNTSDELLCPRYGGEVETIYTHHQKTMIVDAAFVGGLDL

CNGRFDTPKHPLFRTLKTIHKDDFHNNFADDGPREPWHDLHSKIDGPAAYDVLANFEERW

MKASSLLRIPESWHVQVFRSIDSSSVATGDMSIHAAYVKAIRSAQHFIYIENQYFLGSSK

NLGANNLIPMEIALKIANKIRAREKFAAYIVIPMWPEGAPTSNPIQRILYWQHKTMQMMY

QTIKALVEVEPQDFLNFFCREPMIYVHSKGMVVDDEFVLIGSANINLWAEHLGFEVRRVR

QLSELNWRQYKYPVFL

>AtPLDDD

YVTVVVALARTRVLKPLWDEKFGKIPVRDIASGESGKTAIFIDMKFTPFDQYRSQAHVMD

GTLPAIGLDNYEHGKCWEDICYAISEAHHMIYIVGWSIFHKIKLVRETKTLGELLKYKSQ

EGVRVLLLVWDDKTSIKTPGVMGTHDEVLSPRYASVVGTLFTHHQKCVLVDTAFIGGLDL

CDGRYDTPEHRILHDLDTVFKDDFHNTFGTKAPRQPWHDLHCRIDGPAAYDVLINFEQRW

RKATALIRISENWHVQIFRSIDSGSVAEADKSIQTAYIQTIRSAQHFIYIENQYFLGSSR

DAGADNLIPMELALKIVSKIRAKERFAVYVVIPLWPEGDPKSGPVQEILYWQSQTMQMMY

DVIKELKAVHPLDYLNFYCREPMIYVHAKGMIVDDEYVLMGSANINLWAEHLGFVLKKVN

TISEENWKRFKYPLHS

>OsPLDFF1

YLNHFLMIVNSRE--------LKQDPIFDVSPMDKEH-----------------------

-----------------------------EIFITDWWLCPELYLRRPFHRLDILLESRAK

QGVQIYILLYKEVSLLKKQRLLNIHENVKVLRYPDTGIYLWSHHEKIVIVDNCYIGGLDL

CFGRYDTPEHKVVDVPPSIWGKDYYNRERTKYPRMPWHDVQCALYGPACRDIARHFVQRW

RNKAMVIPIKEWWETQVVRSVGPWSATTQEGSIHNAYFSLIEKAEHFVYIENQFFIGLSG

DDTIKNRVLEALYRRILRAEKEKRCFRVIIVIPLLPGGIDDGGAVRAIMHWQYRTICRGP

NSIKNLYDVKAHDYISFYGHGPQIYVHSKLMIIDDRMTLIGSANINLWAEHLGLHPGEVS

QIMDNIWMGYSFPLES

>OsPLDFF2

YLNHFLLIVNSPEVCPKLKEDLKKDPIFDALPMDKERQTLKLRTRSSSKVKWVCYPHRFG

SFAPPRGLMPIDGEAAFQAIASSIEQAKSEIFITGWWLCPELFLRRPFQRLDALLEARAK

QGVQIYILLYKEVALLKKQKLLNIHENVKVLRYPDSGVYLWSHHEKIVIVDNCYLGGLDL

CFGRYDNSAHKLSDVPPVIWGKDYYNRERTKYPRMPWHDVQCALYGPPCRDVARHFVQRW

RNKAMVIPPVNWWEMQVIRSVGQWSATTQEGSIHNAYFSLIEKAEHFVYIENQFFIGLSG

DETIKNRVLEALYRRILRAEREKKRFKAIIIIPLLPGGIDDGGAVRAIMHWQYRTICRGP

NSIQNLYDVKAHDYISFYGHGPQIYVHSKLMIIDDRITLIGSANINLWAEHLGLHRGEVS

HIMDNIWMAYSFPLES

>OsPLDAA1

YSTIDLAVGRTRMITPRWYESIGYLPVQELLNGENRESKIHVKLQYFDVSKWACKAHVPD

NFIPKIPLADYEPHRCWEDIFDAISNAQHLIYITGWSVYTEITLVRDSNTLGELLKKKAS

EGVRVLMLVWDDRTSLKRDGLMATHDEVLCPRNPDSISTMFTHHQKIVVVDHSFVGGLDL

CDGRYDTQYHSLFRTLDSTHHDDFHQNFKKGGPREPWHDIHSRLEGPIAWDVLYNFEQRW

RKQGLLLQLSETWNVQLFRSIDGGAAAAKDRSIQDAYIHAIRRAKNFIYIENQYFLGSSE

DIGALHLIPKELALKVVSKIEAGERFTVYVVVPMWPEGVPESGSVQAILDWQRRTMEMMY

TDIEALQAKNPKDYLTFFCREPMIYVHTKMMIVDDEYIIIGSANINLWYEHLGFQVQKVN

RIAEKYWDMYSYPIKS

>OsPLDAA2

YATIGLAVGRTRTLAPRWYESIGYLPVRDVLAGDTRTGKVHVKLQYFDISKWGCKAHVPD

GFIPRIPLDGYEPHRCWEDIFDAINGARHFIYITGWSVYTEIALIRDADTLGELLKKKAG

EGVRVLMLVWDDRTSLKKDGLMATHDEVLCPRNPDQISTMFTHHQKIVVVDHSFVGGLDL

CDGRYDTPFHSLFRTLGTAHHDDFHQNFAKGGPREPWHDIHCRLEGPVAWDVLYNFEQRW

RKQGLLVQLAESWNVQLFRSIDGGAAAARDRSIQDAYIAAIRRARSFIYIENQYFLGSSE

DVGALHLIPKELSMKVVSKIEAGERFTVYVVVPMWPEGIPESGSVQAILDWQRRTMEMMY

TDIHAIQAKDPKDYLTFFCREAMIYVHTKMMIVDDEYIIVGSANINLWYEHLGFQVRKVN

AMADRCWDLYTYPVKS

>OsPLDAA3

YATIDLAVGRTRVVDPRWYEVIGYLPVRELLSGEGRRPTIHVRLQFRDVAGWGCRAHVPD

AFAPRIPLAGYRQGRCWEDVFDAISNAKHLIYLTGWSVYTEITLIRDGTTLGELLKRKAS

EGVRVLLLVWDDRTS--KWGFMSTHDAVLCPRNPDQIAYMITHHQKTVIVDHSFVGGLDL

CDGRYDTQFHSLFRTLDTAHHSDFHQNLTKGGPREPWHDIHSKIEGPAAWDVLYNFEQRW

RKQGLLLDMAEAWSVQLFRSIDGGACAARDRSIQDAYIHAIRRAKNFIYIENQYFLGSSE

DIEALHLIPREISLKIVNKIEAGERFAVYVVLPMWPEGPPASGSVQAILDWQRRTMEMMY

YDIVALEAKDPRDYLTFFCREPMIYVHSKMMIVDDEYIIVGSANINLWYEHLGFLVQRVN

KMADKYWDLYTYPVKG

>OsPLDAA4

YATVDLALGRTRVIDPRWDERIDYLPVGDLLSGEHKNPTIHVRLQFKDVAVWGCGAHVPD

TFAPTIPLAGYQQGRCWEDVFDAISNAKHLIYITGWSVFTDITLIRDPSTIGELLKRKAS

EGVRVLMLVWNDVTSLQKWGFSQTHDAVVCARHPDKVPFASTHHQKTVIVDHSFVGGLDL

CDGRYDTQSHSLFRTLDAAHHKDFHQSIAKGGPREPWHDIHSRLEGPVAWDVLYNFEQRW

RKQSLLVNLEEAWNVQVFRSIDGGACAARERSIQDAYIHAIRRARDFIYIENQYFIGSSE

DVEAVNLIPRELSLKIMSKIAAGERFTVYVVVPMWPEGHPDSQAMQAILDWQRRTMEMMY

ADIGALKAKDPRDYLTFFCREPMIYVHSKMMIVDDEYIIVGSANINLWYEHLGFLVRRVN

EMANKHWELYTYPIKA

>OsPLDAA5

YATVDLALGRTRVIDPRWDERVDYLPVRDLLSGEDKKPTIHVRLQFKDVAAWGCRAHAPD

TFAPRIPLAGYQQGRCWEDVFDAISNAKHLIYITGWSVFTDITLIRDPSTIGELLKRKAS

EGVRVLMLVWNDVSSLHKLSVAQTHDEVLCPRQADKVSLLATHHQKTVIVDHSFVGGLDL

CDGRYDTQSHSLFRTLDAAHHKDFHQSIAKGGPREPWHDIHSKLEGPIAWDVLYNFEQRW

RKQSLLVNLEDAWNVQLFRSIDGGACAARERSIQDAYIHAIRRARDFIYIENQYFIGSSE

DVEAVNLIPRELSLKIVSKIAAGERFAVYVVVPMWPEGHPGNEAMQAILDWQRRTMEMMY

YDIVALKANDPRDYLTFFCREPMIYVHSKMMIVDDEYIIVGSANINLWYEHLGFVVRRVN

AMADRHWQLYTYPVLA

>OsPLDAA6

YATVDIAVARTRTVEPRWKESVGYLPVDEGLAGA-GRDKIRVQLRFTGVAAWGCRAHIAD

GFAPRIQLAGYEPRRCWEDVFDAISSARRMVYVAGWSVNTDVVLVR---TLGELLKRKAE

QGVMVLLLVWNDRTSIRRDGLMATHDQVLCPRNPDETATMFTHHQKTVIVDGSFLGGIDL

CDGRYDTQEHPLFRTLDTTHRGDFHQNFAKGGPREPWHDIHCRVEGPAAWDVLDNFEQRW

RKQALLVT--EHWNVQVFRSIDGGAAAAAERSIQDAYIHAIRRARDFIYVENQYFLGSSE

GINALHLVPRELSLKIASKIAAGERFAVYVVVPMWPEGVPESDSVQAILDWQRRTMEMMY

RDVAAIQAKDPTDYLNFFCREPMIYVHAKTMIVDDEYIIVGSANINLWHEHLGLLVRLVN

QAARRHWDAFAYPVKS

>OsPLDAA7

YATIGMAVARTRATDPQWTEETAYLPADDVGGGKKRKDKVHVQLRFTDVMSWGCKAHVA-

---PP--LAG---SRCWEDVFDAVANARSLVYIAGWSVSTDVALVRDPRTLGHLLKSKAG

ERVAVLLLVWDDRAAARRDGRMGARGEVVCPRDAV-----FTHHQKAVVADGAFLGGIDL

CGGRYDTQEHPLFRTLATAHRDDFHQSFAKGGPREPWHDVHCRIEGPAAWDVLDNFEQRW

RGQGALLA--QEWHVQVFRSIDSRAVAARERSIQDGYIHAIRRAKYFIYIESQCFLGSSK

NAVAPHTIPKELSLKLASKIRSGDSFRVYVVLPMWPEGVPESATVQAVLDWQRRTMEMMY

KDVAALAARNPREYLSFFCREPKINVNANIMIVDDEYIIVGSANVNLWREHLGFQMSRVN

QAARQHWDMYAYPVSS

>OsPLDAA8

YVDVDVAVARTREVEPVWNQSVGRVPAARVATGEEGHPKLRVRLRFLGVESWDCRSHLSG

GFDPGVRLAGYRPARLWEDMYVAIRDARRFVYVAGWSVNADITLVRDASTLGELLRRKAD

EGVAVLVMPWQDKTSLGNGGLMRTHDEFLCPRNADEVAAEFTHHQKTVTLDASFIGGIDL

CDGRYDDENHTLFRDLDTTYRHDFMQNFRRGGPREPWHDVHCRLEGRAAWDVLANFEQRW

RKQACLLDAEEPWNVQVFRSIDDASVAAADRSIQAGYVEAIRRARRFIYVENQYFLGGCR

DAGCLNLVPVEIALKVAAKIRRGERFAAYVVTPMWPEGEPAGDSVQAILRWNRLTVEMMY

GIVKAIDDAHPCDYLNFFCREPPIYVHAKLMIVDDEYVMVGSANLNLWHEHLGFLVRAVR

RAAEATWDAYPFPIRS

>OsPLDBB1

YVTIQVAVARTYVVPPVWTQNFGSIPAEKLLFGENGKAVLRLSIQYIPVAQYHNRAHVPD

GCLPDFCLDHYQHGQCWRDIYDAICQARRLIYIVGWSVFHTIHLIREGVSLGELLKMKSQ

EGVRVLLLVWDDPTSIKTDGFMGTRDELLCPRSAGETGTIFTHHQKTVILDAAFVGGLDL

CGGRYDTPSHPLFRSLQTVHKEDYYNNFDARGPREPWHDLHSKIDGPAAYDVLQNFQERW

LKASALLSIPETWHVQVFRSIDSNSAATRDMSIHTAYVNAIRGAQHFIYIENQYFIGSSK

DIGANNLIPIEIALKIANKIKAKERFSAYIVIPMWPEGNPTGAPTQRILYWQHKTMQMMY

ETIRALKEEEPQDYLNFFCREPMVYVHSKGMIVDDEYVIIGSANINLWAEHIGFNMRRVR

QIGEQNWERFKYPVFR

>OsPLDBB2

YVTVQLAVARTYVVNPVWAQHFGVVPAEQLEAGEAGKAVLRLSVQYIPVARYHGRAHVPE

GSLPEIRLGNYRQGQCWHDVYDAISQARRLIYITGWSVFHTIQLVRDGGSLGDLLKRKSQ

EGVRVLLLVWDDPTSIQMEGYMGTRDELLCPRSAGETGTIFTHHQKTVILDAAFVGGLDL

CGGRYDTPTHPLFRSLQTLHKDDYYNNFDAQGPREPWHDLHSKIDGPAAYDVLTNFEERW

LKASTLLWIPERWDVQIFRSIDSNSVATSDMSVQTAYVNAIRGAQHFIYIENQYFLGSSK

DVGANNLIPIEIALKIANKIYANERFSAYIVIPMWPEGNPTGAPTQRILYWQKKTMQMMY

EVIKALKEVEPQDYLNFFCREPMVYVHSKGMIVDDEYVIIGSANINLWAEHIGFSTRQVR

HIGEQNWRQFKYPVFL

>OsPLDDD1

YVTLSVAVARTRVIPPVWDERFGTIPADRVASCQNGRTALRLRLRFNPAADYRGRAHYRE

GDLPEIELDEFDHNACWEDICHAILEAHHMIYIVGWSVYDKVRLVREPSNLGELLKFKSQ

EGVRVCLLVWDDKTSIKTGGVMATHDEVLSPRYASVVGTLFTHHQKCVLVDTAFIGGLDL

CDGRYDTPEHRLFKDLDTVFDNDYHNTFAKGGPRQPWHDLHCRIDGPAAYDVLKNFEQRW

RKATALIKISENWHVQVFRSIDSGSLASKDKSIHTAYVRAIRSAQHFIYIENQYFLGSSV

NSGADNLVPIELALKIASKIRAGERFAVYVVIPMWPEGVPTAASVQEILFF--QTMEMMY

RIIQELKAMHPQDYLNFYCREPMIYVHAKGMIVDDEYVILGSANINLWAEHLGFKVNYVN

EIAEENWRRFKYPVPT

>OsPLDDD2

YVSVCLAVAQTRVIPPRWEERFGSLPVDRILSGASSNPELRLSVQYRPIDDYRGGAHVAD

GGLPAIQIAGYEHGRCWEDICHSIVEAHHLVYMVGWSIYHPVKLVREPTTLGELLKKRAR

EGVRIVILLWDDKTSLKTDGVMHTHDEVLVPRYASVVGTLFTHHQKCVLVDTAFIGGLDL

CDGRYDTPEHRLFKDLDTAFNKDFHNTFNSYGPRQPWHDLHCKIEGPAAFDILTNFEQRW

RKATTLIKMSENWHVQVFRSIDSGSVAESDKSIHSAYVKAIRSAQHFIYIENQYFIGSSK

SAGADNLIPVELALKIASKIKANEQFAVYIVLPMWPEGIPTAAPMQQILFWQSQTMSMMY

KIIDALQMQHPQDYLNFYCRECMIYVHSKGMVVDDEYVIIGSANINLWAEHLGFGVRRVR

EMAEENWRAYCYPLQT

>OsPLDDD3

YVSVCLAVAQTRVIAPKWDEHFGSVPVENITPGDYSNPELHLSIQYKPIEQYKGMAHIPD

DFCPKIEIDGYEQNKCWEDICHAIAEAHHLIYIIGWSLYHPVKLVRESTTLGGLLKTKVQ

EGVRVIVLLWDDKTSLKTDGLMHTHDEVLAPRYASVVGTLFTHHQKCVIVDTAFIGGLDL

CDGRYDTPEHRLFKDLDTVFKDDFHNTFNKSGPRQPWHDLHCKIEGPAAYDILTNFEQRW

RKSATLVKMSENWHVQIFRSIDSGSVAESDKSIHNAYVKAIRSAQHYIYIENQYFIGSSR

SAGAENLIPIELAIKIARKIKARERFAAYIVIPMWPEGNPTTAAMQEILFWQGQTMSMMY

KIVEALQKEHPQDYLNFYCRESMIYVHSKGMIVDDEYVLIGSANINLWAEHLGFRVRQVN

EMAEENWARYRYPIHS

>OsPLDGG

RLLLLLLLAAGAPAA------VQSIPVPGVLSADQGNKSLDVLAQY-----WQ-------

---PKNPKSG--------DYGYSESEMVRFGADKGQRVY-------------KALEKAAD

RKIK-----------VQHSGFAPDFDK----------------HAKVWISDKVYIGSA--

---NND------------------------------WKSLSQVKEGIYFKTVEIYFQNLW

--NSVAWD--KQWQARLPPSVDVPYVFETFQADEQGWLDTIKSVKFGVRISTQYA-TQT-

---------------LSSAISEVKNATVRILVAYWTH-------TEKYL-----------

---------KSLLYSNILCTSPTRVNHGKYA-VSDVRANIGTSNL-IWDYYTGFGVSQLQ

DIFDADWFSYTVPVAS

>TpPLD1

YATIDFAVARTGVVSPVWNESVSRIPVSEILRGKDSEAQILVRLHFSHVTEWGCKAHLSE

QFSPRIELDGYSPPRLWEEMYECINGARKFIYITGWSVYAQITLVRDR-TIGELLKKKAD

QGVRVLVLIWEDRTALNKEGLMKTHCDVLCPRSPDEVGAQFTHHQKTVSLDFSFVGGIDL

CDGRYDTETHTLFGDLNSVFRDDFVQNFRHGGPREPWHDAHSKIQGPAAWDVITNFTERW

KKQA-LLDLPETWNVQILRSIDDGSVSPNDRSIHSAYVEAIRRAKNFIFIENQYFLGSSQ

DCGCLHLIPIELALKIVSKIKAKERFAVYVVTPMWPEGFPDGDTVQAILHWHRNTVEMMY

KLIEALTEQHPTDYLNFFCREPMIYVHAKLMIVDDEYLIVGSANLNLWYEH-RYLVRTVR

EIAKKAWDAFTYPVTS

>TpPLD2

YATIDFAVARSRVVKPTWNETVSRIPISEILSGKDSEAQIQVRLQFSHVTEWRCMTHLSQ

HFLPRIELQGYNPPRLWEEMYEYINGAQIFIYIAGWSVYTEFTLVRDRETLGELLKKKAD

QGVRVLVLIWEDRTALKNEGLMRTHCDVLSPRDPDEVEVQFTHHQKTVSLDASFVGGIDL

TNGRYDTEKHTLFGDLDTIYKDDFLQNFRHGGPREPWHDAHSKIEGPAALDILANFTQRW

TKKT-LLDMTETWNVQIFRSIDDGSVSALDQSIHCAYVHAIRRAKHFIYIENQYFFGSCR

DCGCLHLVPIEIALKIVSKIRAKERFAVYVVTPMWPEGIPEGDTVQAILHWNRNTIEMMY

AMIEALREEAPTDYLNFFCREPLIYVHAKLMIVDDEYIIMGSANLNLWYEHFGYLVRLVR

GIAKRAWDVFPFPVKS

>TpPLD3

YVSVQVAVAQTRIISPAWKEHFGAISAVDVLSGAYGKSALRLSIQFFPVQTYKGMAHVRD

HLHLPIRLDGFERGRCWEDICHAILEAHHLIYITGWSIYHKIKLVREPTNLGELLKYKSQ

EGVRVLLLVWDDKTSIKTDGVMQTHDEVLSPRYGSVVGSLYTHHQKTVIVDSAFIGGLDL

CDGRYDTPEHRLFRDLQTDKKEDYHNTFDAGGPRQPWHDLHSKIDGPAAYDILTNFEQRW

RKTTSLIKISDNWHVQVFRSIDSGSVAENDKSIHTAYVKAIRSAQHFIYIENQYFLGSSR

NAGANNLIPMELALKTADKIRKNERFAVYVVIPMWPEGVPNSAAVQEILFWQTQTMEMMY

AIIDAIKDAHPQDYLNFFCREAMIYVHAKGMIVDDEYVIIGSANINLWEEHLGFDVQKVK

KMAENHWLQFKYPVPG

>TpPLD4

YVSVQLAVAQTRVINPIWDEHMGAISAKRVLRGENGLASLRIAMKFSPAQNYGGMAHGED

SSFPNIELEGFERKNCWEDMCHAILEAHHLVYIAGWSIYTKIKLIREHTNLGQLLKFKSQ

EGLRVLLLVWDDKTSIKTEGIMQTHDEVLSPRYASVVGTLYTHHQKLLIVDASFVGGLDM

CDGRYDTPRHPLFKTLKTSHKDDYHNTFDTGGPREPWHDMHCKIEGPAAYDVLTNFEQRW

RKATALINMSETWHVQVFRSIDSGSVAEIDMSIHTAYVKAIRSAQHFIYIENQYFIGSSK

NAGANNMIPIELALKVASKIRKNEKFAVYIVIPMWPEGVPTGSVVQEILFWQGQTMQMMY

DIIKALQDAHPQDYLNFYCREPMIYVHAKGMIVDDEYVMLGSANINLWAEHLGFLVKRVN

SLAEQYWNQFKYPVPA

>TpPLD5

YVTISLAVARTRVISPVWNQHVGAIPAEKLMSHQNRKAALKLSMQFFPIENYRGKAHMPD

NLLPPVQLDHYENGKCWQDICEAILQARRFIYITGWSVYHKIKLVHGSGTLGDLLKFKSQ

EGLRVLLLLWDDPTSIKTEGLMGTHDELLCPRIAGEVGTIFTHHQKSVLLDSAFLGGLDL

CDGRYDTPEHHLFRTMQTVHKDDYHNTFDFGGPREPWHDLHCKIDGPAAYDVLTNFEQRW

TKAASLLKLPETWHVQIFRSIDSSSVAEADMSIHTAYVEAIRSAQHFIYIENQYFIGSSN

ALGANNMIPMELSLKIVEKIRADERFAVYILVPMWPEGNPTSLAMQRILYWQGQTMQMMY

ETIKALKEAHPQDYLNFFCREPMIYVHSKGMIVDDEYVILGSANINLWAEHLGFHVRRVR

DISESYWKQFKYPVLG

>TpPLD6

YMWVLLLLCS---FS------VQSIPVPGVLSGDRGNGKLDIIAQY-----WE-------

---PNNPNSG--------DYGYSAADLERFRAPVGLSVF-------------KSLQRAAD

RNVS-----------VQHSGVAPEYDE----------------HAKVWISDEIYIGSA--

---NND------------------------------WKSLTQVKEGIYIEKVEVYFNNLW

--NSTLWD--KEWQVKLPSRVETPRVLDTYQTDEQGWVDTIKSAQFKVRISTQYV-KEP-

---------------LSSAISQVKHATVKLLVAYWAH-------TEQYL-----------

---------QSLLYTNVLCSTATRVNHGKYA-VSDVRAHIGTSNL-IWDYYTGFGVAQLT

NIFDADWNSYAFPVNT

>TpPLD7

----------------------------------K-------------------------

-FAFP-------------------------------------------------------

----FYIL-------------------------------------------------LDF

SY-----------------W-----KCE--------------------------------

---------------QVLRSVGQWSATSQETSIHAAFLSLIEKAEHFIYIENQFFIGLEG

DDIIQNRISQALYDRIIRAHTEKKCFRVIVLIPLLPGGVDDSGAVRAIMHWQYRTICRGK

NSLERLGNEKVPNYVSFYGYGPQIYVHSKLMIVDDRTVLIGSANINLWSEHLGLQGPEIN

KIKDFLWREYSFPLES

>TpPLD8

YATIDLAVGRTRILQPRWYESIGHVPVKDLLNGEGGEGRIHVKLRYFDVTNWSCKAHMPD

KFMPRIPLSGYQPHRCWEDVFDAITNAKHLIYITGWSVYSHIRLIRDSRILGELLKRKAG

EGVRVLMLVWDDRTSMKKDGLMATHDEVLCPRNPDQIATMFTHHQKIVVVDSSFVGGIDL

CDGRYDTQFHSLFKTLNDVHHDDFHQNFEKGGPREPWHDIHSKLEGSIAWDVLFNFEQRW

RKQGLLLDIDETWHVQLFRSIDGGAAAAADRSIQDAYINAIRRAKDFIYIENQYFLGSSE

DINALHLIPKELSLKIVSKIEAGEPFRVYVVVPMWPEGEPESGSVQAILDWQRRTLEMMY

TDIQALRAKSPKDYLTFFCREPMIYVHSKMMI----------------FQH---------

----------------
